# The shape of educational inequality

**DOI:** 10.1126/sciadv.aaz5954

**Published:** 2020-07-15

**Authors:** Christopher L. Quarles, Ceren Budak, Paul Resnick

**Affiliations:** School of Information, University of Michigan, Ann Arbor, MI 48109, USA.

## Abstract

Hundreds of thousands of students drop out of school each year in the United States, despite billions of dollars of funding and myriad educational reforms. Existing research tends to look at the effect of easily measurable student characteristics. However, a vast number of harder-to-measure student traits, skills, and resources affect educational success. We present a conceptual framework for the cumulative effect of all factors, which we call student capital. We develop a method for estimating student capital in groups of students and find that student capital is distributed exponentially in each of 140 cohorts of community college students. Students’ ability to be successful does not behave like standard tests of intelligence. Instead, it acts like a limited resource, distributed unequally. The results suggest that rather than removing barriers related to easily measured characteristics, interventions should be focused on building up the skills and resources needed to be successful in school.

## Teaser

The ability of community college students to be successful is conceptualized, measured, and shown to be exponentially distributed.

## INTRODUCTION

Over 500,000 high school students and over 600,000 college students drop out of school every year (*1*, *2*). Practitioners, researchers, and pundits have proposed a variety of explanations for why so many students are unable to achieve their goals. However, despite a variety of different education policies and billions of dollars spent ensuring no children are left behind, millions of children and adults are unable to achieve their academic goals. Unfortunately, there is no simple explanation that can point to simple interventions. The process of becoming successful in school can be complicated and difficult, requiring the right combination of social, personal, academic, and financial traits and skills. Researchers and policy makers have not yet found the secret to consistently cultivating success in students. So, it is no surprise that so many students are unable to successfully navigate the educational system.

### Student capital

Here, we present a conceptual framework for studying students’ capacity to be successful in school, which we call student capital. We also demonstrate an analytical method for measuring this quantity in community college students. Broadly speaking, we define student capital as the cumulative amount of resources a student can bring to bear to be successful in a particular school context. These resources might come in many forms, such as economic resources, social, cognitive, noncognitive, and academic skills. Like other forms of capital, these resources both help drive students toward their goals and insulate them against the random shocks that affect all of us. For example, consider the unlucky situation of a commuter college student whose car has broken down and therefore may have to miss class. If the student has a supportive social network, then she might be able to catch a ride with a friend. Strong academic skills might ensure that missing a class does not affect her learning. Cultural capital and self-confidence might give her the ability to communicate clearly with her instructor to minimize any effects on grades. Economic resources might have allowed her to live in a campus dormitory, possibly at a more elite college, thus avoiding the situation in the first place. And of course, the more resources she has, the better off she will probably be.

There is a rich body of literature examining the factors that affect student persistence in college. An incomplete list of personal factors related to college success includes academic preparation (*3*, *4*), students’ self-discipline, self-confidence, commitment to college, amount of social activity, race, age, full-time/part-time enrollment, degree expectations, distance between home and school, number of hours worked at a job, parents’ income, parents’ education level (*5*), perceptions of faculty, peer groups, campus engagement (*6*), familial responsibilities, interest in school, lack of money (*7*), unreliable housing, and food insecurity (*8*). In addition, the skills required to be successful in a classroom can be confusing and vary from class to class (*9*, *10*), with some researchers calling classrooms a “black box” (*11*, *12*). So, college success may be partly attributable to a student’s ability to learn new classroom expectations. Many researchers have attempted to disentangle this web of causal relationships (*13*). They face substantial challenges from selection bias and hidden variables. Our goal is not to wade into that discussion but to consider the cumulative effects of all factors as a single variable.

We call this quantity student capital to fit with prior literature on other forms of capital, which can be used for both a source of investment income and a reserve of resources to insulate against shocks. Social scientists have examined a variety of forms of capital, including economic, social, and human capital. Social capital is some form of social relationship that can be used to benefit individuals or groups, such as membership in neighborhood groups (*14*), networks of parents (*15*), or professional connections (*16*). Human capital is the set of skills embodied in a given workforce, typically measured in terms of economic benefit. For example, researchers have examined the wage benefits of specific skills, on-the-job training, and a variety of college degrees (*17*–*19*). Different forms of capital can manifest in multiple ways, and there have been many debates about how to measure them (*14*, *15*, *17*, *20*, *21*). Economic and social capital are most often defined in terms of what they consist of: money and social relationships, respectively. In contrast, human capital is usually defined in terms of economic benefit and can consist of many types of embodied skills and traits. Student capital is more like human capital, since it is defined by the educational success it can provide.

### Operationalizing student capital

We consider student capital as the amount of success that a student is able to achieve. In our study, this is the number of credits they could earn if that many credits were required for their goals. To compare with more financial forms of capital, in an economic system without a standardized currency, an individual’s wealth can only be determined by what it can be traded for. Depending on the circumstances, a bag of gold might be worth more or less than a bag of rice. Similarly, a supportive family or good social skills may have a varying effect on a student’s outcome, depending on a variety of situational factors. Furthermore, those factors might interact in a way that helps or hinders the student. We operationalize student capital as the total amount of educational success (credits completed) that can be “bought” by a student, in their particular context, using their skills, traits, and resources. This runs the risk of conflating student capital with the returns on student capital. However, given the notable universalities in our results, we think that this metric measures something meaningful.

It is important to distinguish student capital from student outcomes, which might include whether a student graduated or transferred to a 4-year school. Outcomes are measurable representations of whether a student reached a certain goal, rather than giving a measure of how well they could have done. Student capital is harder to measure in individuals. The student capital of students who have dropped out of school can be directly observed as the number of credits they earned. However, students who graduated or transferred may not have run out of student capital. We only know that their capacity to earn credits is greater than or equal to the number they earned. This is good for those students but makes data analysis more challenging. It makes it impossible to measure the student capital of every student. Instead, we can estimate the distribution of student capital in a group using right-censored maximum likelihood estimation. This is still useful, since statistics like this are commonly used to describe groups of students.

### The shape of inequality

Despite humankind’s best efforts, inequality has always been with us (*22*). However, the amount of inequality has varied, depending on the era and location (*20*, *22*). This suggests that, beyond the idiosyncratic forces unique to specific groups, there are systemic forces that keep resources allocated unequally. To better understand those forces, we look at the shape of educational inequality. In a more economic context, studying the shape of inequality might involve examining income or wealth distributions (*23*, *24*). In the context of education, we look at the shape of student capital distributions. If we find that these distributions have the same universal shape across colleges, then this will give us insight into the underlying macroscale processes that create educational inequality.

To analyze the shape of educational inequality, we used data from 156,712 students from 28 Washington community colleges. We grouped students into 140 cohorts, each containing the students who started at the same college during the same academic year. We focus on degree-seeking community college students who aim to transfer to a 4-year college. This group has the benefit of being fairly diverse (*25*) while sharing the same goals and educational context. This allows us to measure their student capital on the same credit-based scale. For each student, we calculated the total number of community college credits they had earned within 5 years of enrolling and whether the student dropped out of school without earning a degree or transferring to a 4-year college.

### Models for student capital distribution

**Fig. 1:**
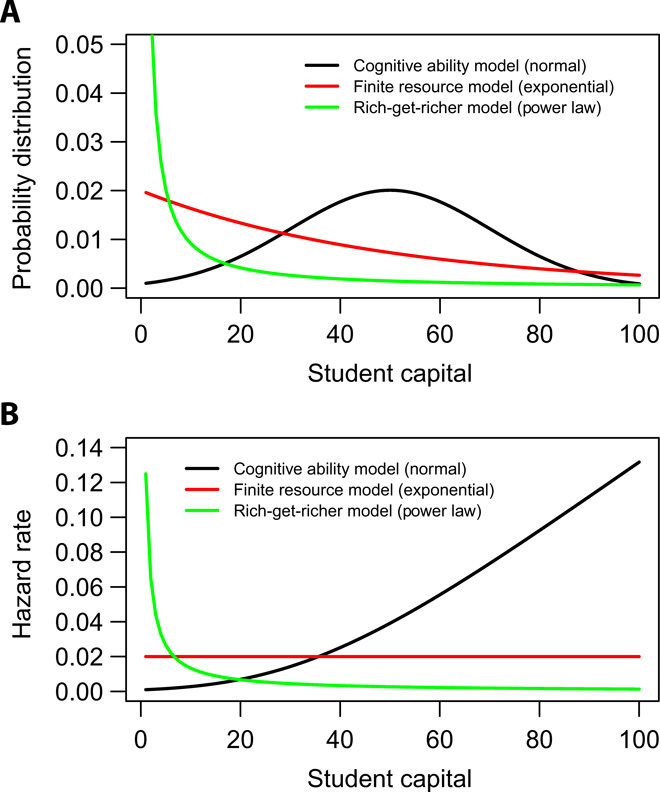
Potential models of student capital distributions. (**A**) Probability distribution function for each model. (**B**) Hazard rate of dropping out specified by each model. Specifically, the vertical axis gives the probability that a student who has *k* units of student capital will stop their education before earning *k* + 1 units. In both cases, the trends suggested are qualitative, designed to show the shape of the distribution rather than any specific numbers.

To explore the shape of educational inequality, we consider a number of different models for how student capital might be distributed. Each model represents a universe with plausible educational behavior that leads to a particular distribution of student capital. Graphical comparison of the models is shown in Fig. 1. In the next section, we test whether these models fit the data.

The cognitive ability model comes from the claim that educational outcomes are largely determined by, or equivalent to, cognitive ability as measured by achievement tests such as intelligence quotient (IQ) (*26*). This model is consistent with the common practice in the education literature of using standardized tests such as the SAT as measures of ability or achievement (*27*, *28*). These tests measure specific cognitive abilities or knowledge at the time the student takes the test. The cognitive ability model assumes that student capital, which is nominally a student’s ability to navigate successfully through the complex social, personal, and academic demands of a school system, is mostly dependent on IQ or cognitive abilities. Since these cognitive abilities, as measured, tend to be normally distributed, this model suggests that student capital might also be shaped like a bell curve.

It could also be possible that, like forms of financial capital, a given community has a limited amount of student capital that can be generated in their college-going population. Collectively, parents, family, and friends may have a finite amount of experience, noncognitive skills, social stability, and financial resources to share with children and college-bound adults. Some communities, particularly wealthier and more educated ones, have more of this resource than others. This is consistent with the well-established fact that children of wealthier and more educated parents tend to have more of the skills necessary for academic success (*5*, *27*, *29*). The finite resource model assumes that the only thing constraining students’ capacity to complete college is the limited nature of this resource in a population. For this model, we assume that resources are at least partially substitutable so that we can treat these resources as coming from a single pool. For instance, a student whose home is too unstable for studying may be able to spend money to work at a coffee shop. If society distributes this finite student capital in the least informative way, we would expect to see an exponential distribution of student capital for a given community. Put another way: If, in a given population, the only major limitation is that student capital is finite, then there are many ways that it could be distributed to individuals. However, in this case, the vastly most probable distribution is exponential—or something very close. More details and examples of the principle of maximum entropy, which underlies this model, can be found in (*30*–*33*). A similar model was proposed in (*34*) to explain why income distributions between the 10th and 90th percentiles are distributed exponentially (*24*).

The rich-get-richer model assumes that the student capital gained from an additional resource is roughly proportional to the student capital they already have. For instance, a student with good study skills might be able to benefit more from increased wealth, because they might be able to use the time not working at a job to study more efficiently. The rich-get-richer phenomenon has been well studied in a variety of other areas (*35*–*37*) and leads to a heavy-tailed distribution such as a power law or log-normal distribution. Since these distributions often have similar behavior and can be difficult to distinguish from each other, we focus on whether the distribution of student capital fits a power law.

Of course another mental model, often implicitly assumed among those who do educational interventions, is that (i) interventions and college policies can have a substantial effect on student progress at various points in the college process and (ii) the policies and supports in different colleges vary enough to see this effect. If this context-specific model were true, we would expect distributions of student capital to have varying, idiosyncratic shapes depending on the school itself and perhaps even the year. For example, a college with a strong student onboarding program might have a mode at 15 or 30 credits, while other colleges with regular enrollment cycles might have periodic distributions of student capital. In this case, institutional structures would be more important for student success than the resources, skills, and traits student brought with them. Student capital would be a relatively unimportant consideration in educational success. This model is not shown in Fig. 1, because the context-specific model would imply that each cohort of students has its own distinctive curve.

## RESULTS

**Fig. 2:**
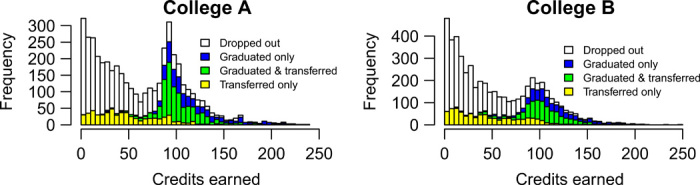
Distribution of credits earned. Each graph corresponds to the distribution of students in one college in the dataset within 5 years of enrolling. White bars represent students who dropped out.

Unfortunately, we cannot directly measure the number of credits that every student could have earned. Instead, we only have data for the number of credits students actually earned and whether they dropped out, graduated, or transferred. Figure 2 shows the distribution of credits, graduation, and transfer for two colleges. Figure S1 has similar graphs for all colleges in the dataset. White bars represent students who dropped out so that their observed number of credits is equal to their student capital. Blue, green, and yellow students represent censored data points. These individuals’ student capital is greater than or equal to the observed number of credits shown on the graph. The number of successful students peaks around 90 to 100 credits, because associate’s degrees in Washington require at least 90 credits. Note that Fig. 2 and fig. S1 do not show student capital, just the observed number of earned credits. Most graduating/transferred students will have student capital values larger than the number of credits they earned. So, we can imagine what the student capital of successful students might look like by flattening the colored bars to the right. The distribution of student capital might look like Fig. 2, but with a smaller bump. Or it could be continually decreasing so that the number of students who have *k* credits of student capital decreases as *k* increases.

### Testing the models

The cognitive ability, finite resource, and rich-get-richer models each assumed that the distribution of student capital follows a given parametric model: normal, exponential, or power law. So, we explored them all using the same approach. We assumed that student capital is distributed according to the specified model, with a censoring process corresponding to graduation/transfer, which is estimated individually at each credit level. We used right-censored maximum likelihood estimation to estimate the parameters for each model. We then examined goodness of fit for each model using both the Akaike information criterion (AIC) and quantile-quantile (QQ) plots.

AIC is a standard information-theoretic method for comparing distribution fit. If a model has *K* parameters and log-likelihood ℒ, then *AIC* = 2*K* − 2ℒ. We used AIC to compare the fit of the three parametric models on each of the 140 cohorts. The finite resource/exponential model gave the best fit to the data on every cohort (table S2). So, the inferred distribution of student capital fits an exponential distribution better than a normal or power law distribution.

**Fig. 3:**
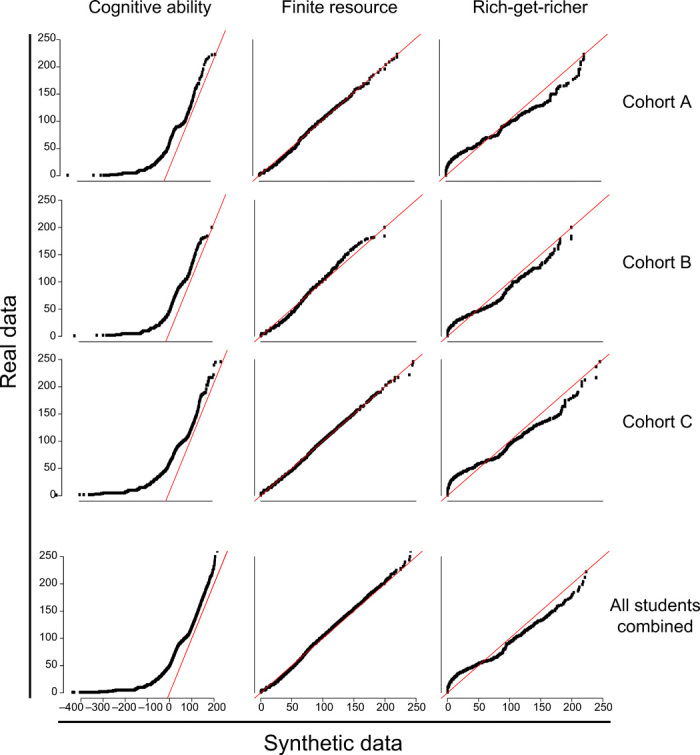
QQ plots for the three parametric models. Each plot compares a real dataset to a simulated population generated using a fitted model. The columns correspond to discrete normal, geometric, and zeta distributions, respectively. The top three rows each correspond to a single college-year cohort. The bottom row infers the distributions for the complete set of 156,712 students from all 28 colleges. Points close to the red line indicate that the quantiles of the simulated data are very close to the quantiles of the actual data, signifying that the model fits the data better.

While the AIC analysis shows which of the chosen distributions is better, it does not show if that fit is good. To qualitatively examine goodness of fit, we used QQ plots. To generate a QQ plot for a given cohort of students, we fit the parameters for each of the three models and then used those parameters to generate a set of simulated students. We then compared the distribution of simulated students’ credits earned to the actual distribution of credits earned. Figure 3 shows QQ plots using this process for three representative cohorts and also for the combined set of all students. QQ plots and AICs for all cohorts can be found in fig. S2 and table S1.

The finite resource model fit the data well. Using both QQ plots and AIC, this model fit best across colleges and across years. After accounting for the censoring effect of students graduating and transferring, student capital seems to follow an exponential distribution.

The rich-get-richer model fit the data very poorly. The power law seems to expect more students dropping out early and more students with very high student capital than is found in the real data. The heavy-tailed behavior found in the power law distribution is inconsistent with our data, suggesting that other heavy-tailed distributions such as log-normal would be poor fits as well.

Because students in our dataset only earned positive integer numbers of credits, we used a truncated discrete normal distribution for the cognitive ability model. At first glance, the cognitive ability model seems to fit the data almost as well as the finite resource model. The QQ plots for the normal distribution are reasonably close to the diagonal. However, the results were not consistent with what we would consider a normally distributed population. In such a population, the mean will be between the minimum value and the maximum value. However, for every cohort in our dataset, the inferred mean was $\hat{\mu }=1$. This was the minimum possible number of credits earned and also the minimum allowed $\hat{\mu }$ using our algorithm. Inferred parameter values, $\hat{\sigma }$, were distributed between 90.5 and 169.0 credits [mean($\hat{\sigma }$) = 120.1, SD($\hat{\sigma }$) = 12.7]. These values of $\hat{\sigma }$ tend to be larger than the number of credits earned by most degree-receiving students. These pathological results are consistent with a continually decreasing probability distribution of student capital. The best way to fit a normal distribution to a decreasing distribution is to just fit the right tail. The resulting simulated data are missing the characteristic bell curve shape of the normal distribution. Therefore, we cannot say that the cognitive ability model is supported by our results.

The context-specific model assumes that the shape of the distribution of student capital is highly dependent on the college. Evidence for this model would involve very different distributions of student capital, with some colleges having high dropout rates for students with low numbers of credits and others having high dropout rates at higher credit levels. However, our previous analysis shows that distributions of student capital, across years and across colleges, all fit an exponential model very well. It seems that colleges do not have a substantial impact on the shape of educational inequality. At every college, there are more low-resourced students than high-resourced students.

### Student capital as a finite resource

**Fig. 4:**
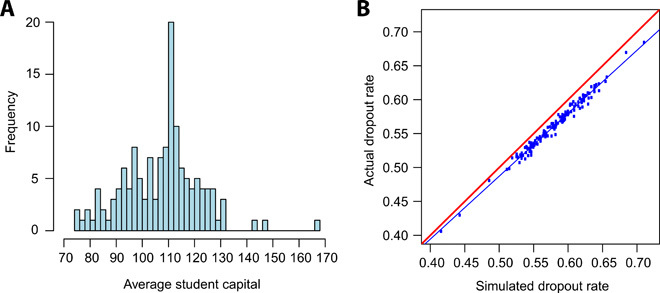
Further analysis of the finite resource model. (**A**) Histogram of average student capital. Each data value is the average student capital of a single college-year cohort. (**B**) Comparison of actual dropout rates and the dropout rates estimated by the finite resource model (*n* = 140). Each point corresponds to one college-year cohort. The red line corresponds to both values being equal. The blue line is the line of best fit.

We now explore the finite resource/exponential model in more depth. Colleges often want to compare the experiences of different groups of students. Because the exponential distribution can be uniquely characterized by a single parameter, we can use our model to assign a number to any group of students. This number can be used like any other statistic, such as graduation rate. One possible such parameter is the per-credit retention rate *q*, which is one minus the traditional exponential decay rate. For example, one college might find that 95% of their students, at any credit level, will take one more five-credit class. This corresponds to *q*^5^ = 0.95, or *q* = 0.9898. Another such parameter is the mean of the distribution $\mu_{S}=\frac{1}{1-q}$. This has the units of credits and is reasonably easy to interpret as the average student capital in the student population. Equivalently, *n*μ*_S_* is the total amount of student capital collectively possessed by a group of students. Both *q* and μ*_S_* can be easily inferred with the algorithm we used. Figure 4A shows the distribution of average student capital μ*_S_* for the 140 cohorts in the dataset. Student populations in most of the colleges we studied have an average student capital between 90 and 130 credits, with a peak around 110 credits. It may seem surprising that most students drop out of school, given that the average student capital in most cohorts is larger than the typical 90 to 100 credits required for an associate’s degree in Washington. The high dropout rate comes from the fact that the exponential distribution is right-skewed. Some students would be able to achieve very high levels of education, which pulls up the average student capital but only increases the number of graduates by one. Note that the values in Fig. 4A are specific to Washington state community colleges. Schools that measure credits differently, such as those on a semester system, will not be able to compare their average student capital with Washington’s quarter system.

Typically, education regression models include *R*^2^ values to show how well the model explains variation in a set of data. So, we calculated the amount of variance in college dropout rates explained by our model. Again, our process is as follows: (i) select a cohort of students; (ii) fit the finite resource model, which involves inferring the decay rate for the exponential model and inferring the full distribution of success points; (iii) generate 10,000 simulated students using this new fitted model; and (iv) compare the percentage of simulated students who dropped out with the percentage of actual students who dropped out. Figure 4B is a plot of these percentages, with one point for each college-year cohort. The figure shows that the estimated dropout rate is close to the actual dropout rate. However, the estimates are systematically biased so that the model estimates are systematically higher than the true values. The relationship is very strongly linear (*R*^2^ = 0.982, *F*_1,138_ = 7731, *P* < 0.001), which means that the actual dropout rate could be reconstructed with high accuracy from this biased estimate. This reconstructability means that the combination of the exponential parameter and the distribution of success points contain effectively all of the information contained in college dropout rates.

This approach assumes the full distribution of success points, which involves estimating the percentage of graduating/transferring students at every credit level. In pursuit of simplicity, we repeated the process without such a strong assumption. For each cohort, we took the mean of the success point distribution, effectively assuming that all students would graduate or transfer at the same credit value. This simplified two-parameter model still explained 92.4% of the variation in dropout rates by cohort (*F*_1,138_ = 1680, *P* < 0.001).

## DISCUSSION

This paper has presented a conceptualization of student capital as a many-faceted resource, operationalized it, and shown that there is a universal shape to educational inequality. This shape suggests that, in a given population, the amount of student success is finite. The results have ramifications for how colleges think about student success and interventions. In addition, the informationally equivalent parameters mentioned here—the per-credit retention rate *q* and the average student capital μ*_S_*—might be used to compare groups of students. For instance, they could be used to compare demographic groups.

We defined student capital using an input-based approach: as the resources that students can marshal toward achieving their academic goals. In contrast, the more common practice of measuring student outcomes is an output-based approach. The cohorts in this study started in different years and came from colleges with different policies, geographies, and populations. However, in all cases, the shape of educational inequality was the same.

Student capital distributions across colleges and years were surprisingly all exponential distributions. This model explained 98% of the variation in graduation rates of cohorts in our dataset.

Our explanation for this systemic inequality is that student capital is a finite resource in a given population. Society has a limited amount of student capital to distribute to the community college-going population and distributes that capital in the least informative way possible. Student capital as a finite resource makes sense if ability to be successful in school is truly a form of capital that one gathers from parents, mentors, and friends. Throughout their life, people gain things like social skills (*29*), academic skills (*38*), emotional regulation (*39*), and economic resources (*40*) from their environment. Geographic areas that are less educated and poorer have fewer resources like this. So, they have less ability to share that student capital with their college-bound population.

We have also discarded a number of hypotheses that are common in scholarly and popular conceptions of academic achievement. Many of the colleges were running interventions focused on student success (*41*, *42*), which we might expect would change the shape of the student capital distribution. Despite their attempts, the general shape of the student capital distribution was similar across cohorts. It seems that small-scale interventions do not have a substantial effect without affecting students throughout the college-going process.

Students’ ability to earn college credits has a fundamentally different distribution than that of intelligence and academic achievement tests. This is consistent with previous research showing that tests of knowledge have limited relationship with more comprehensive measures of ability to be successful in school, like grade point average (GPA) (*3*, *43*). Even students who are academically knowledgeable are subject to different types of knowledge tests and to instructors with wildly varying grading practices (*44*–*46*) and pedagogical practices (*9*, *10*). Successfully navigating school at least partially amounts to learning and adapting to the particular expectations of teachers and school bureaucracy. The results also caution researchers against cavalier use of the word ability to describe test scores. An individual’s ability to do well on standardized tests, which might more aptly be called cognitive ability, is not the same as student capital, the ability to complete schooling.

Nor does student capital follow the power law behavior of a rich-get-richer model. In some sense, this is unsurprising. Many of the examples we have of power law behavior, such as social media follower networks (*47*) and academic citation (*48*), require a negligible cost for each additional unit of capital. Given that each additional college credit has, at minimum, a financial cost, we would not expect to see power law behavior in this regime. However, wealth and income distributions do have heavy tails at the high end (*24*, *49*). So, it would not be surprising if there was an unobserved tail of students who had nearly unlimited ability to be successful in school.

We think that these results will be useful for the design of student success interventions. These interventions often focus on finding and reducing barriers in the college-going process. However, students face a great many barriers, most of which are outside of the college’s influence (*7*). This paper suggests that successful educational interventions should be focused on building up resources and skills in students rather than minimizing barriers. Interventions that focus on resource building are also likely to improve life outcomes in the broader sense. Results from comprehensive, resource-building interventions show significant returns (*50*). Although these interventions are more costly, the benefit to society is lower than the cost (*51*).

A common concept in community colleges is student momentum. Our results suggest that we might instead think of student capital as a form of energy. The exponential distribution of student capital is very similar to the Boltzmann-Gibbs distribution in physics, which has been used to study economic capital (*49*). In this formulation, the average student capital μ*_S_* is a state variable corresponding to the average energy of the students in the system. Colleges might conceptualize interventions that focus on increasing the energy of their student body.

A few notes of caution are warranted to readers trying to generalize or extend our work. For our analytical technique to work, there needs to be a sufficient number of uncensored data points to infer the distribution. These are dropouts that, sadly, community colleges have in plenty. High schools and more selective colleges likely have too few dropouts to accurately make an inference.

It is also worth emphasizing that randomness can play a role in a student’s ability to be successful. An inspirational teacher or an unexpected financial challenge may have a huge effect on a student’s outcomes. This randomness creates error in the use of credits to measure individuals’ student capital. When looking at groups of students, this error should average out. Some people will have the inspirational teachers and some will not.

The institutional context also plays a role in student persistence and completion. For example, the skills necessary to thrive in a low-income high school may be very different from those required in an elite university. So, a student who has a lot of student capital in one school may have less in another. Most differences in student persistence by college are associated with the differences between 2- and 4-year institutions and college selectivity. After controlling for the student populations, other factors seem to have a relatively small effect (*6*, *13*, *52*).

## MATERIALS AND METHODS

### Data

We use deidentified data provided by the Washington State Board for Community and Technical Colleges (SBCTC), which included all students who started at 30 of the 31 community/technical colleges in Washington within the 5-year period between summer 2006 and spring 2011. One college declined to participate. The original dataset contained 303,390 students. To create a group of people with nominally similar goals, we only included degree-seeking students who self-identified as academic transfer students during their first quarter. We excluded reverse transfer students, dual-enrollment high school students enrolled through Washington’s Running Start program, and anyone who enrolled but earned zero credits. We also excluded two colleges that had less than 100 transfer students. The remaining colleges each had over 1000 transfer students. This reduced the dataset to 156,712 students, split into 140 college-year cohorts. Descriptive statistics on students and cohorts can be found in figs. S3 and S4 and table S2. Data exploration was initially performed on 4 of the 28 colleges. These four were chosen to have different general shapes and to have a sufficient sample size. Once the statistical methods were designed and written, we then examined the remaining colleges.

There were two main observable variables of interest. The first was *x_i_*, the number of community college credits each student earned within 5 years of enrolling in the Washington community college system. We did not differentiate between credits based on when they were earned. We assume that students are putting resources into being successful in college at the rate that is optimal for them. Our other observable variable is ${\overset{ˇ}{y}}_{i}$, a binary variable that describes whether a student dropped out. We say a student graduated if they earned an associate’s or bachelor’s degree in the SBCTC system within 5 years of initial enrollment. All degrees required at least 90 credits, although some students brought credits into the SBCTC system and graduated with fewer than 90 credits in our data. We say that a student transferred if they enrolled at a 4-year college within 5 years of initial community college enrollment. Transfer data were obtained by SBCTC from the National Student Clearinghouse. A student dropped out if they did not transfer or graduate. Analysis was performed using R version 3.5.1 (*53*) using the VGAM package (*54*).

### Statistical analysis

The parametric models assume that each student has two independent latent variables: their student capital *y_i_*, which is the number of credits they can earn before they have to dropout, and their success point *g_i_*, the credit level where they achieve their academic goals by transferring or graduating with an associate’s degree. The observed number of credits is then *x_i_* = min (*y_i_*, *g_i_*). Students who have dropped out (${\hat{y}}_{i}=1$) correspond to the case where *y_i_* < *g_i_*. Otherwise, ${\hat{y}}_{i}=0$.

To test the parametric models, we assume that *y_i_* and *g_i_* are drawn from theoretical probability distributions, infer the parameters of those distributions, and then compare the inferred distributions with the real data. Let *Y_k_* be the probability that a randomly drawn student will have a student capital of exactly *k* credits. Let *G_k_* be the probability that a randomly drawn student has a success point of exactly *k* credits. This gives the likelihood function

$$ℒ=\underset{i}{\Pi }{\left[Y_{x_{i}}\;\sum_{k=x_{i}+1}^{\infty }G_{k}\right]}^{{\overset{ˇ}{y}}_{i}}{\left[G_{x_{i}}\sum_{k=x_{i}}^{\infty }\;Y_{k}\right]}^{1-{\overset{ˇ}{y}}_{i}}$$ (1)

Taking logs and simplifying gives the log-likelihood function

$$\begin{array}{c} \text{log}ℒ=[\sum_{i}\;{\overset{ˇ}{y}}_{i}\text{log}Y_{x_{i}}+(1-{\overset{ˇ}{y}}_{i})\text{log}(\sum_{k=x_{i}}^{\infty }\;Y_{k})\;]+\\ \left[\sum_{i}(1-{\overset{ˇ}{y}}_{i})\text{log}G_{x_{i}}+{\overset{ˇ}{y}}_{i}\text{log}(\sum_{k=x_{i}+1}^{\infty }G_{k})\right] \end{array}$$ (2)

Notice that the only distribution in the left sum is *Y_k_*, while the right sum only includes *G_k_*. So, we can maximize the log-likelihood by maximizing each term separately. The distribution {*Y_k_*} that best fits student capital does so regardless of distribution of success point {*G_k_*}.

We tested the three different parametric models for {*Y_k_*}: the discrete normal distribution $Y_{k}(\mu ,\sigma )=\frac{1}{A(\mu ,\sigma )}e^{\frac{-{\left(k-\mu \right)}^{2}}{2\sigma^{2}}}$ where *A*(μ, σ) is a normalizing constant calculated numerically, the geometric distribution *Y_k_*(*q*) = (1 − *q*)*q*^*k* − 1^, and the zeta distribution $Y_{k}(\alpha )=\frac{1}{\zeta (\alpha )}k^{-\alpha }$ where ζ(α) is the Riemann zeta function. To validate the model, we had to create simulated students, which meant inferring {*G_k_*} as well. Unlike {*Y_k_*}, where we were trying to find a simple parametric form with few parameters, we were only interested in {*G_k_*} as a validation tool. Since {*G_k_*} definitely is dependent on college policies and transfer options, we did not expect a parametric form for it. So, for each value of *k*, we inferred *G_k_* as its own parameter.

## Supplementary Material

aaz5954_SM.pdf

## SUPPLEMENTARY MATERIALS

Supplementary material for this article is available at http://advances.sciencemag.org/cgi/content/full/6/29/eaaz5954/DC1
