# Supplementary material for: The shape of educational inequality
Source: Sci Adv. 2020 Jul 15;6(29):eaaz5954. doi: 10.1126/sciadv.aaz5954 (PMC7363455; doi:10.1126/sciadv.aaz5954)
Supplement: aaz5954_SM.pdf [file aaz5954_SM.pdf]

Science Advances
AAAS

advances.sciencemag.org/cgi/content/full/6/29/eaaz5954/DC1

Supplementary Materials for

The shape of educational inequality

Christopher L. Quarles*, Ceren Budak, Paul Resnick

*Corresponding author. Email: chrisquarles@gmail.com

Published 15 July 2020, *Sci. Adv.* **6**, eaaz5954 (2020)
DOI: 10.1126/sciadv.aaz5954

This PDF file includes:

Sections S1 to S5
Figs. S1 to S7
Tables S1 and S2
References

Section S1. Measurement of Student Capital

There are conceptual reasons for why "credits that a student could earn if they had to" is a good way to measure student capital. For this, it useful to look at the process of earning a degree through a student's eyes. Attending college is a large commitment. It is reasonable to assume that students who have decided to earn a degree are marshalling all the resources they reasonably can towards making progress. Furthermore, if we think about a student's own judgment of what progress toward a degree means, it likely consists of taking classes and earning credits until they have earned enough to graduate. So it is reasonable that a student would put as many of their own resources as reasonably possible towards earning credits. Therefore, the number of credits they could earn makes sense as a measure of their student capital.

Note that our metric doesn't depend on the *speed* at which students earned credits. Unlike popular conceptions of full-time college students at highly selective universities, community college students have varied and chaotic enrollment patterns. Most are enrolled part-time at some point in their college career, and many take at least one term off before returning to school (*55*). We assume that students marshal the resources they need to be successful at the best speed that they can. This just takes longer for some students than for others. Students with limited resources may need to take time off to, for instance, work to earn more money or address the challenges of life.

Section S2. Modeling Distributions & Inequality

We can think of inequality as the unequal distribution of a certain resource, such as income, wealth, social capital, educational success, high-speed internet access or health insurance. It is worth looking at how inequality is distributed in other areas where quantitative, fine-grained data is available. Income is probably the most heavily studied type of inequality, because tax data is readily available in many countries. A wide variety of parametric models have been used to describe income distributions including the Weibull, Dagum, and Singh-Maddala distributions (*56*). Generally speaking, models with more parameters will tend to fit data better at the cost of interpretability. Given von Neumann's statement that he could fit an elephant using four parameters (*57*), a practical approach to understanding distributions might be to find the simplest model that one can meaningfully interpret and use (*58*). In studying Lorenz curves of income, (*59*) found a one parameter family of Lamé curves and used it to build a "trickle-up" explanation of income growth. Because different forces might dominate different ranges of a distribution of inequality, another method is to focus on certain segments of the distribution. Probability distributions of income seem to be exponential between the 10th and 90th percentile, and look like a power law for high earners (*24, 49*). Nirei & Souma (*24*) explained this two-tier structure using a model which combined linear wage growth with

exponential asset growth. In a more information-theoretic approach, Dragulescu & Yakovenko (*34*) derive the exponential portion of the distribution by maximizing entropy subject to the conservation of money.

Wealth distributions have also been studied, though less deeply because of the lack of records on wealth. It's clear that the high end of the wealth distribution follows a power law (*49*, *60*, *61*), consistent with a rich-get-richer mechanism. There is evidence that some portion of the middle wealth levels behave exponentially. However, this varies widely by country. In many countries, the net wealth owned by the bottom 50% of the population is near zero, or even negative (*62*). This likely relates to the fact that survival without wealth is easier than survival with no income.

Another type of inequality involves social capital. Most people are familiar with the incredible popularity of certain social media stars. At the higher end, social media follower distributions tend to follow heavy-tailed behavior found in power law and log-normal distributions (*47*, *63*), consistent with a rich-get-richer effect (*64*). Though some distributions of social network degree follow a power law with exponential cutoff (*65*). In this context, social media networks are informational networks, where the marginal cost of an additional follower is effectively zero, and the social capital might be considered as one's ability to be heard. In cases where individuals need to expend time or resources to maintain friendships, there are limits on the number of friends a single individual has (*66*, *67*).

Section S3. More on Data and Methods

Fractional Credit Values To ensure that we could use discrete distribution functions in R, we rounded all fractional credit values to the nearest integer (<0.4% of data points).

Estimate of G We could estimate the best fit parameter for each possible distribution of student capital without estimating distributions of transfer and graduation. However, to validate the data, we needed to estimate the distribution of the points where students would graduate or transfer. Because of the non-regular nature of student graduation patterns, we fit this distribution at each credit level individually. Specifically, if G_k is the probability that a randomly drawn student in a given group would graduate at exactly k credits, we estimated each G_k as its own parameter. To do this, we numerically maximized the following term in the log-likelihood function. This was the term that only involved G_k.

$$\log \mathcal{L}_G = \left[\sum_i (1 - \check{y}_i) \log G_{x_i} + \check{y}_i \log\left(\sum_{k=x_i+1}^{\infty} G_k \right) \right]$$

Because most classes in the Washington community college system were 5 credit classes, the inferred probability tends to be larger when k is a multiple of 5. This distribution was done for

each cohort individually. The inferred values for the combined set of students from all 140 cohorts is shown in Figure S5. To aid interpretation, the same values smoothed by a spline are shown in Figure S6.

Parametric Validation using QQ Plots We checked each parametric model – normal, exponential, and power law models – using the following procedure:

1. Fit the model to a given dataset, including finding the parameters for both the distribution of student capital Y_k and the distribution of success points, G_k.
2. Generate a synthetic dataset using the fitted parameters.
3. Compare the synthetic dataset to the real dataset using QQ plots. If the model is a good fit, then the two models should compare well.

A visual description of this process is shown in Figure S7. The QQ plots show that the exponential models fit very well, and that the power laws don't fit at all. A full set of QQ plots, one for each college-year cohort, is shown at the end of the appendix. Some cohorts fit the exponential model worse than others. Future work might consider exploring the source of this variation.

Section S4. A Note on the Normal Distribution / Cognitive Ability Model

In our inference of the cognitive ability model, we found that the inferred mean of the truncated normal distributions were all $\hat{\mu} = 1$. This was also the minimum value that our algorithm would allow. To be thorough, we explored the case where the mean was zero or negative. We consider a couple cases.

First, assume that the mean is taken over all degree-seeking transfer students who started a given community college during the same year, and that the left tail of the distribution is just those students who enrolled but didn't earn credits. We would then expect that at least half of students who enrolled did not earn any credits. In our initial data cleaning, we had excluded all students who had enrolled for a positive number of credits, but earned none. We did this because, anecdotally, we had been told that many of those students mistakenly claimed to be degree-seeking transfer students on their application. So we went back and re-included those students in the cohort. Students who enrolled and earned 0 credits made up 7.2% of this larger population of students. This is far less than half, which is not consistent with $\hat{\mu} \leq 0$.

One might also claim that the entire not-necessarily-college-going population is normally distributed, and that the students who actually earn credits in community college are in the right tail of this distribution. Despite the fact that the open-access nature of community college admissions and the incredible diversity of community college students calls this claim into strong doubt, we explore this hypothesis numerically. Unfortunately, our numerical algorithm for optimizing log-likelihood was not designed for this range. So, for two cohorts we graphically

found the maximum of the log-likelihood function using this model. For College 20 Cohort 1, we found a maximum likelihood at $\hat{\mu} = -2148$, $\hat{\sigma} = 441$. For College 111, Cohort 4, we found a maximum likelihood at $\hat{\mu} = -3061$, $\hat{\sigma} = 629$. Both of these statistics would suggest that community college students who earned any credits were 4.8 standard deviations above the mean and in the top 0.00006% of the population. Since the population of Washington is roughly 7.5 million people, this would imply that only 4 people in the state could earn college credits.

This exploration shows us that the log-likelihood function of this model is really pathological in this case, and does not lead to interpretable results. The cognitive ability model just does not make sense when it comes to earning credits in community college.

Section S5. The Exponential Distribution as it Arises from Bernoulli Trials

Entropy maximization is not the only way that exponential distributions arise. Another method for generating exponential distributions comes from repeated Bernoulli trials. This is similar to the mechanism involved in radioactive decay. We explain this idea here, and then explain why we think it doesn't approximate student capital. Consider a large group of students with identical coins. The coins have probability q of coming up tails, and $1 - q$ of coming up heads. If a coin comes up tails, they add one credit to their record and flip again. If the coin comes up heads, they stop flipping and leave school. Then the probability of any student earning exactly k credits will be the exponential distribution $P(k) = (1 - q)q^k$.

One might imagine a group of students working hard at school with random life events causing them to drop out. If students were homogeneous, so that every student's probability of having a catastrophic life event was the same, then this model would be a good one. Each coin flip would be equivalent to an opportunity to have a catastrophic event happen to a student. However, we know that students are nowhere near homogeneous. Community college students are quite diverse in their backgrounds and preparation. Importantly, we can predict, with some accuracy, who will be successful in school (*68*). So this model for generating our observed distributions of student capital just doesn't work.

Fig. S1. Distributions of credits earned by college. (College 30 through College 300)

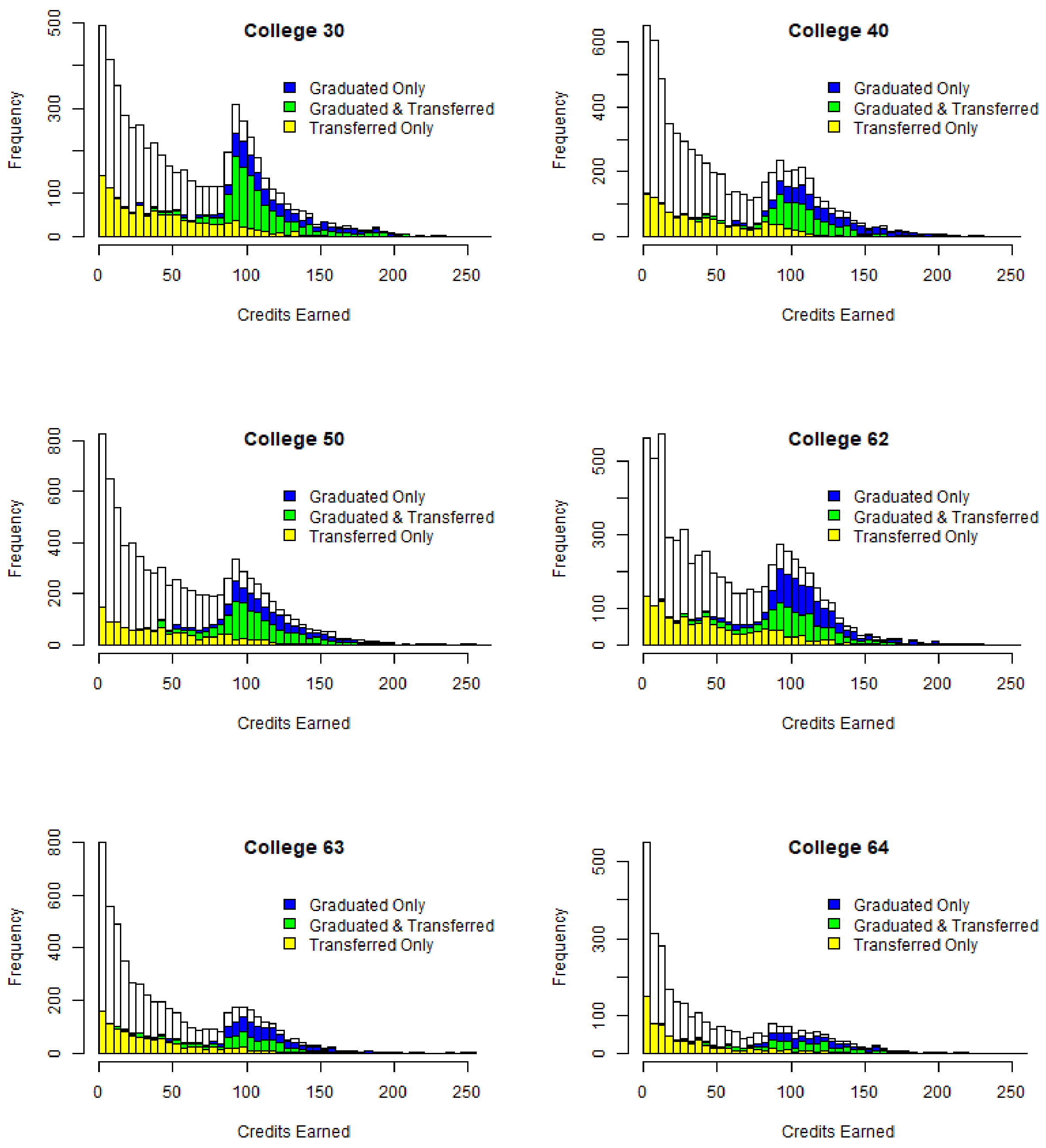

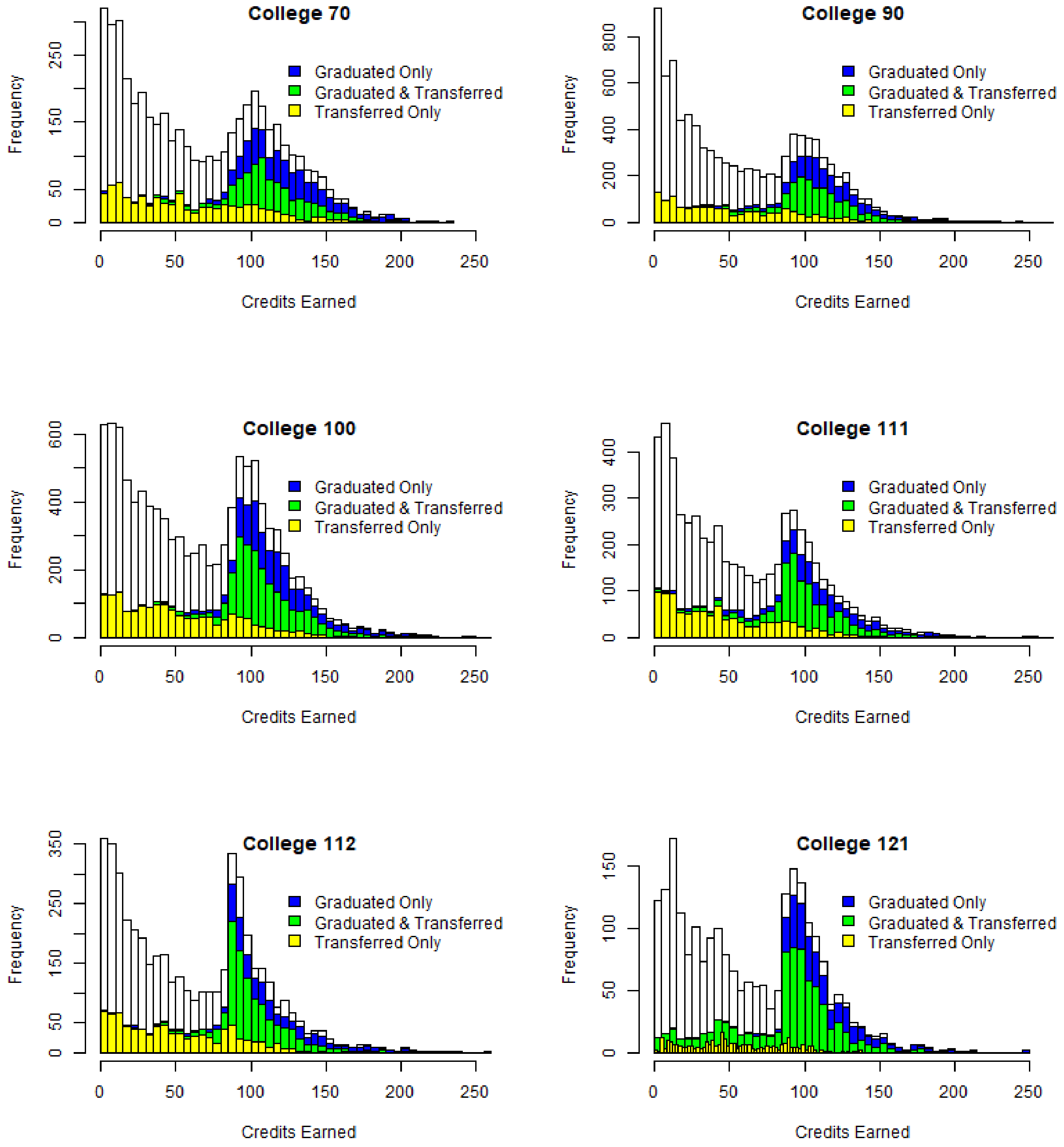
College 70
Graduated Only
Graduated & Transferred
Transferred Only
Frequency
0 50 150 250
0 50 100 150 200 250
Credits Earned
College 90
Graduated Only
Graduated & Transferred
Transferred Only
Frequency
0 200 400 600 800
0 50 100 150 200 250
Credits Earned
College 100
Graduated Only
Graduated & Transferred
Transferred Only
Frequency
0 200 400 600
0 50 100 150 200 250
Credits Earned
College 111
Graduated Only
Graduated & Transferred
Transferred Only
Frequency
0 100 200 300 400
0 50 100 150 200 250
Credits Earned
College 112
Graduated Only
Graduated & Transferred
Transferred Only
Frequency
0 50 150 250 350
0 50 100 150 200 250
Credits Earned
College 121
Graduated Only
Graduated & Transferred
Transferred Only
Frequency
0 50 100 150
0 50 100 150 200 250
Credits Earned

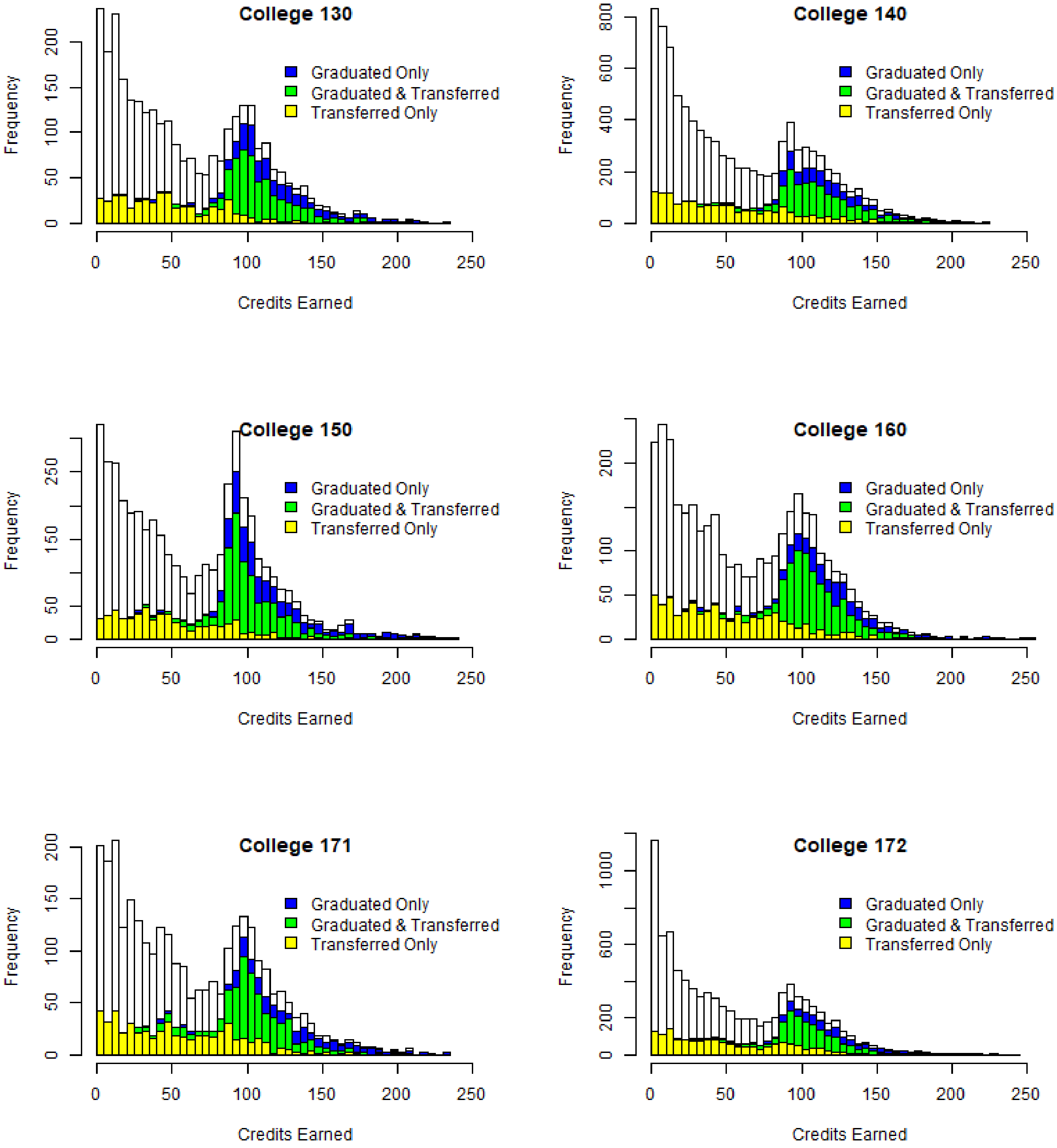
College 130
College 140
College 150
College 160
College 171
College 172
Graduated Only
Graduated & Transferred
Transferred Only
Frequency
Credits Earned
0
50
100
150
200
250

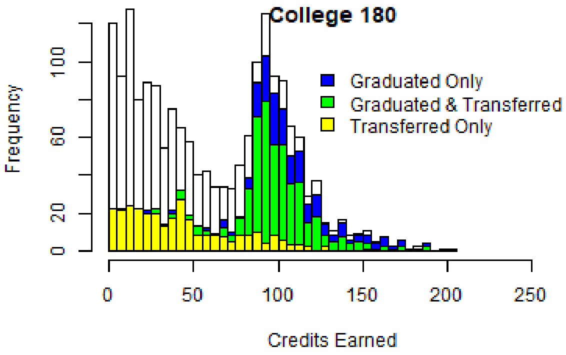
College 180
Graduated Only
Graduated & Transferred
Transferred Only
Frequency
0
20
60
100
0
50
100
150
200
250
Credits Earned

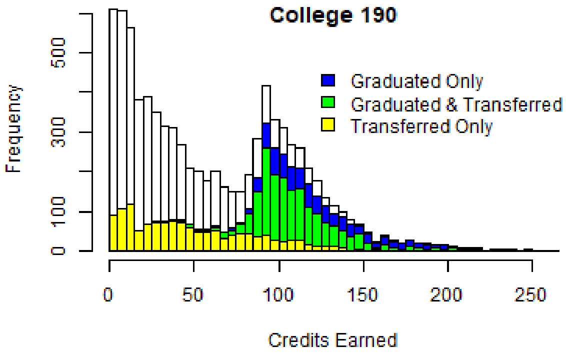
College 190
Graduated Only
Graduated & Transferred
Transferred Only
Frequency
0
100
300
500
0
50
100
150
200
250
Credits Earned

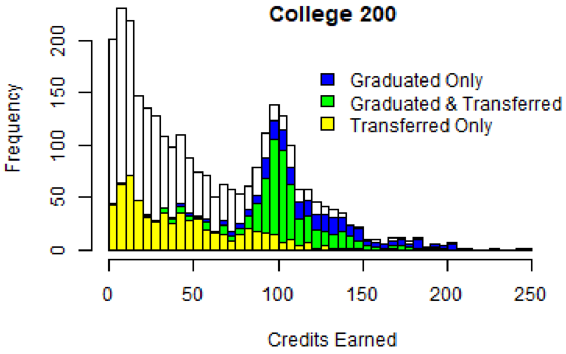
College 200
Graduated Only
Graduated & Transferred
Transferred Only
Frequency
0
50
100
150
200
0
50
100
150
200
250
Credits Earned

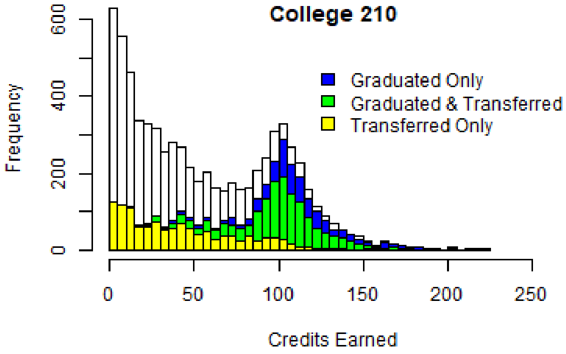
College 210
Graduated Only
Graduated & Transferred
Transferred Only
Frequency
0
200
400
600
0
50
100
150
200
250
Credits Earned

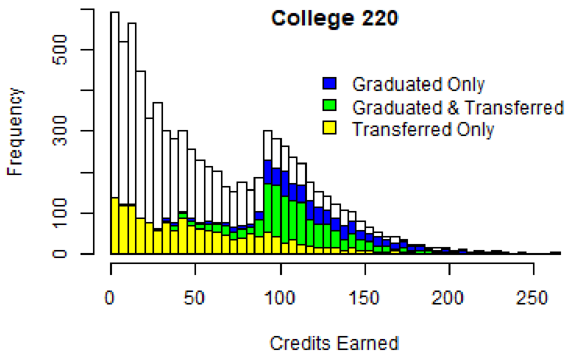
College 220
Graduated Only
Graduated & Transferred
Transferred Only
Frequency
0
100
300
500
0
50
100
150
200
250
Credits Earned

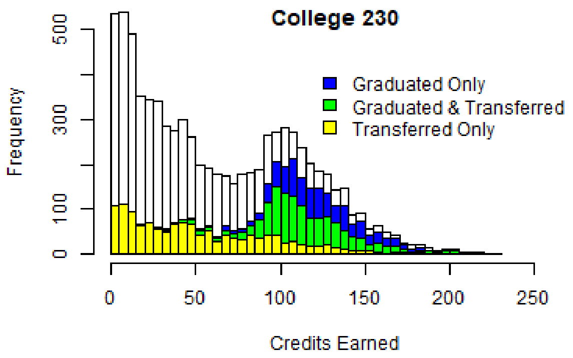
College 230
Graduated Only
Graduated & Transferred
Transferred Only
Frequency
0
100
300
500
0
50
100
150
200
250
Credits Earned

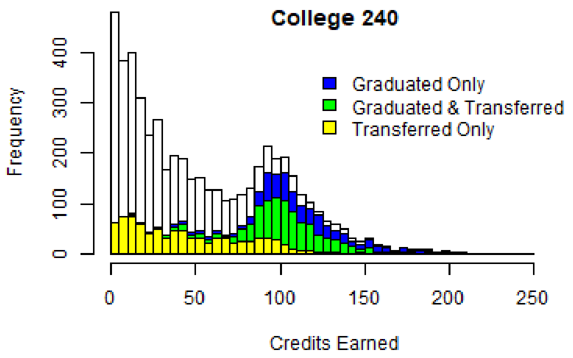
College 240
Graduated Only
Graduated & Transferred
Transferred Only
Frequency
0
100
200
300
400
0
50
100
150
200
250
Credits Earned

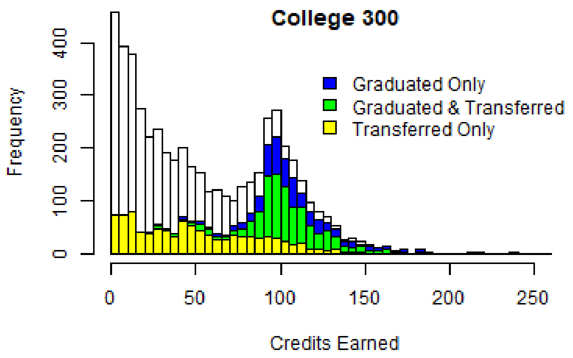
College 300
Graduated Only
Graduated & Transferred
Transferred Only
Frequency
0
100
200
300
400
0
50
100
150
200
250
Credits Earned

Fig. S2. QQ plots for each cohort and model in the analysis. Each cohort consists of all students who started at one college in a single year. (College 10 Cohort 1 through College 300 Cohort 5)

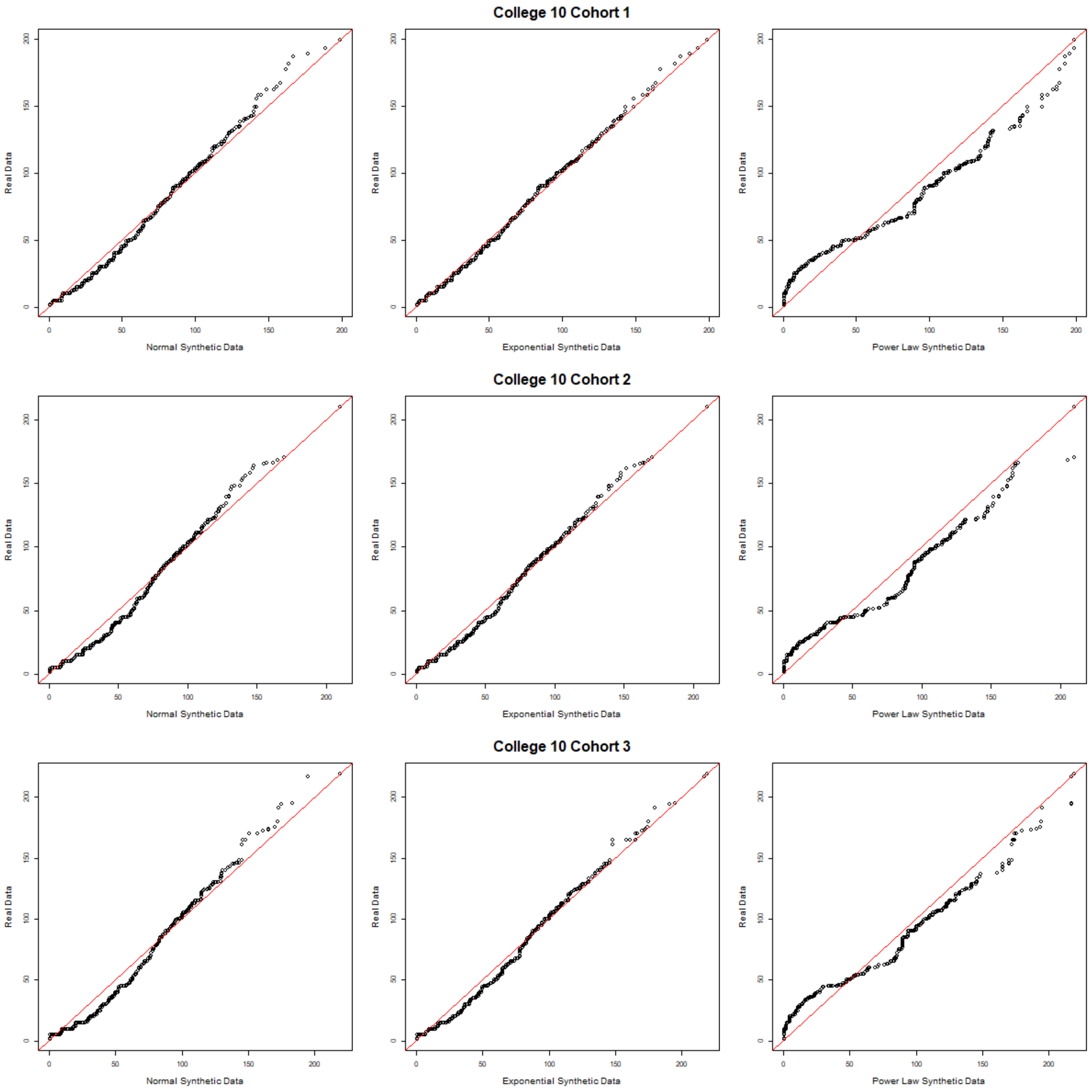

College 10 Cohort 4

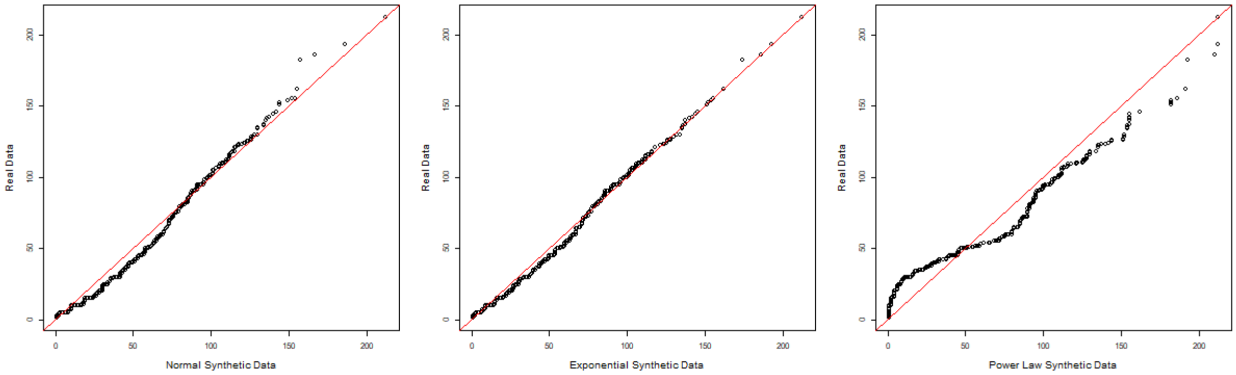

College 10 Cohort 5

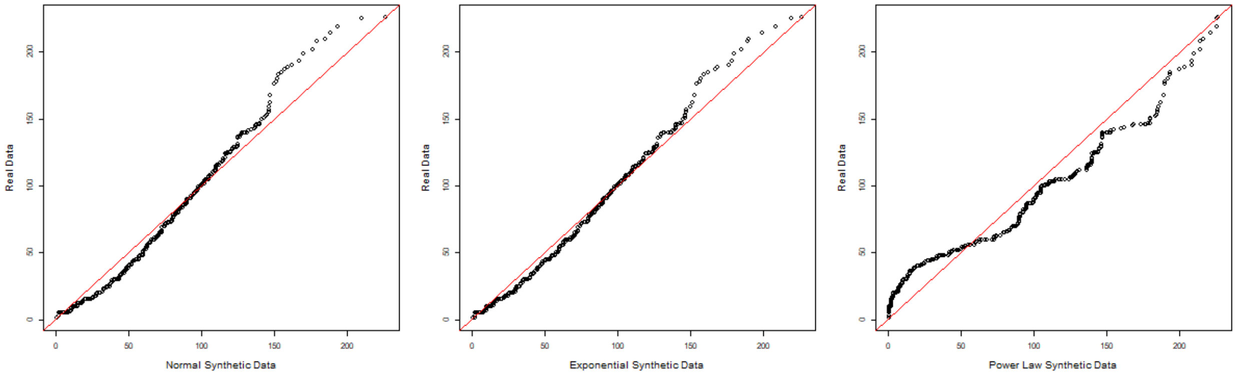

College 20 Cohort 1

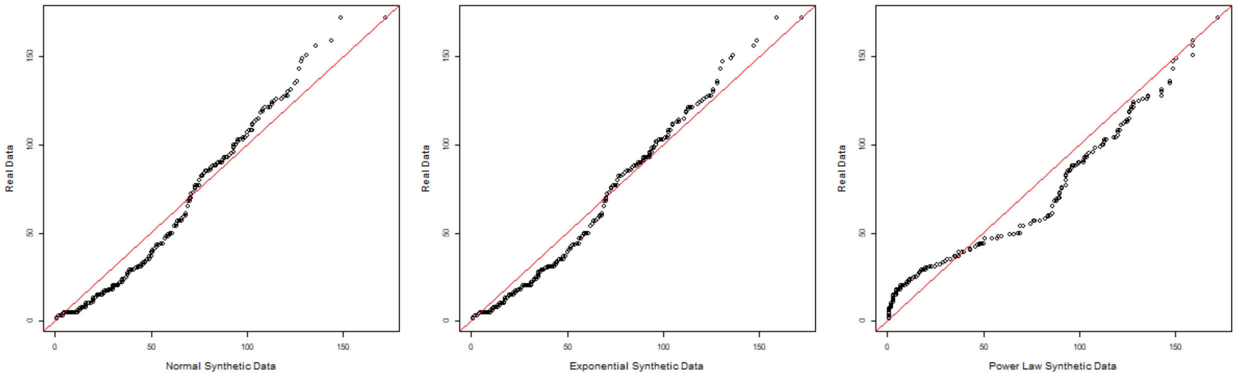

College 20 Cohort 2

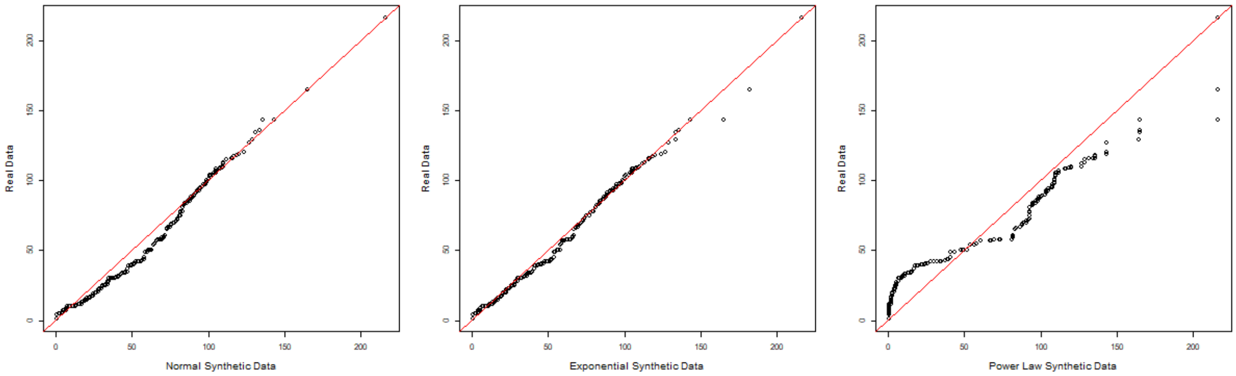

College 20 Cohort 3

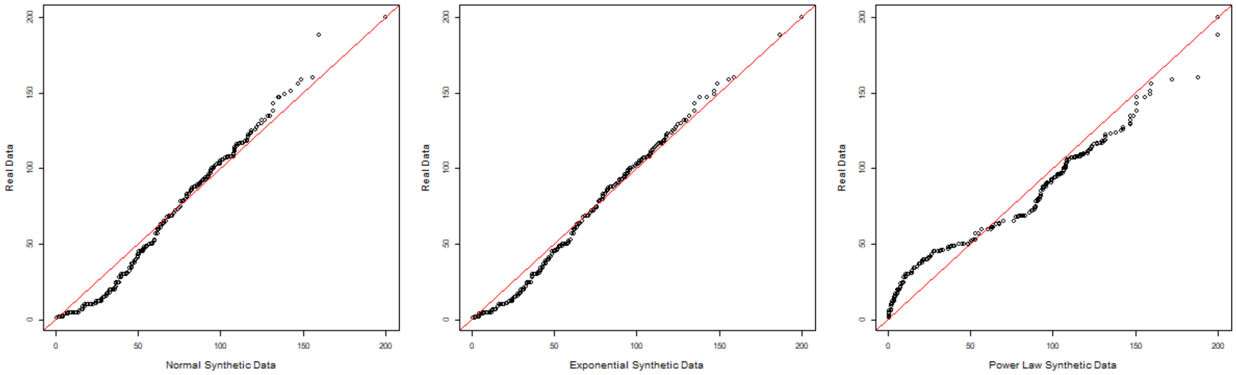

College 20 Cohort 4

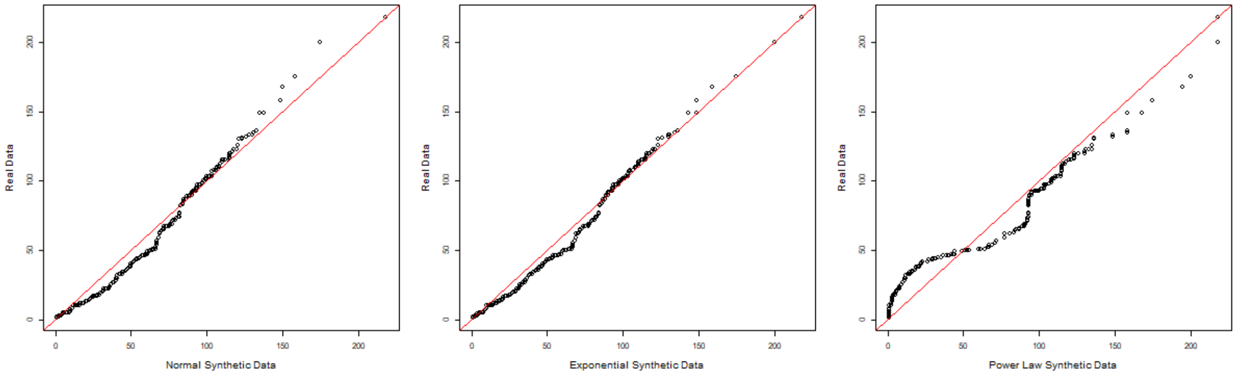

College 20 Cohort 5

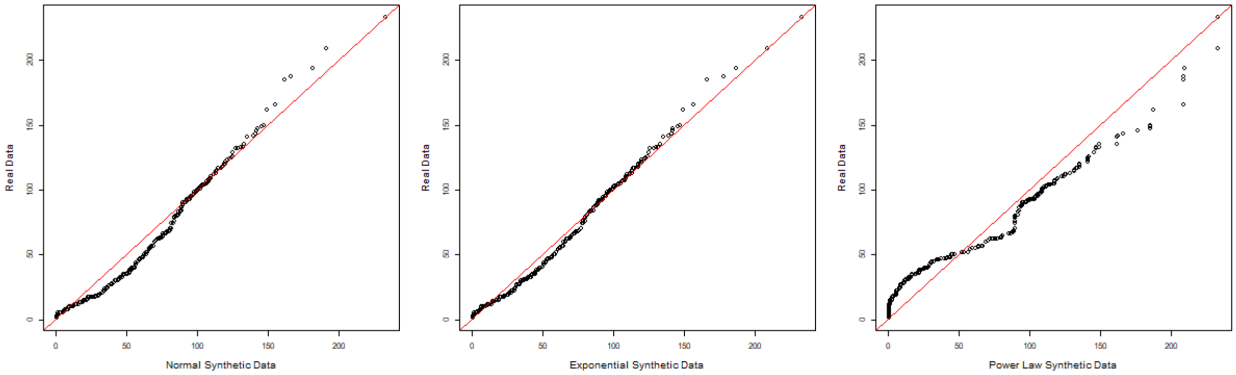

College 30 Cohort 1

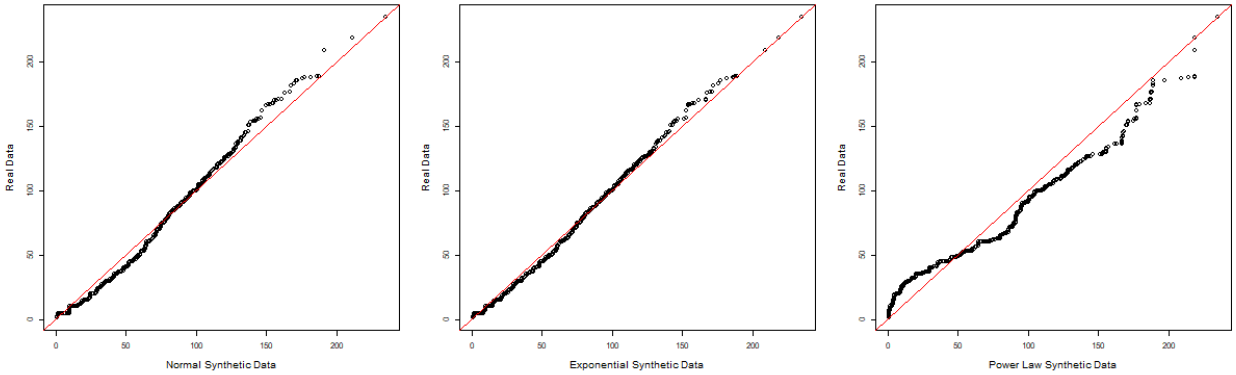

College 30 Cohort 2

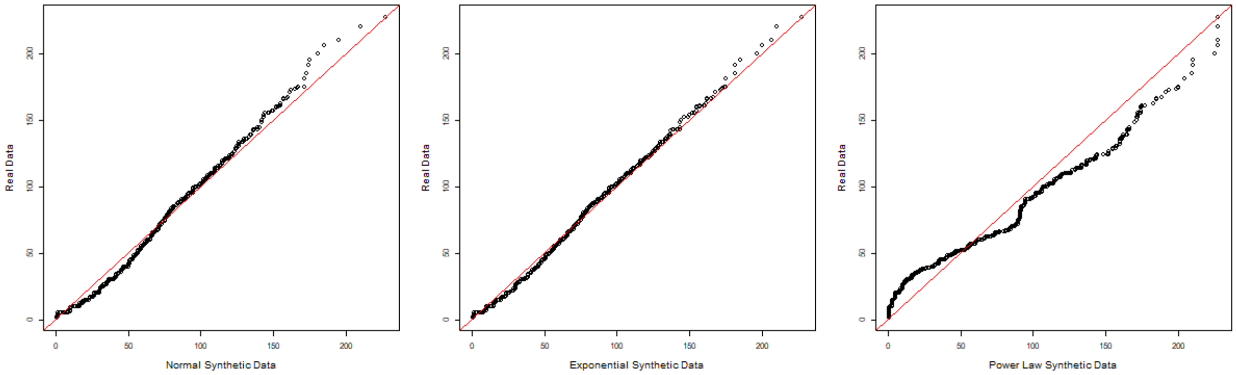

College 30 Cohort 3

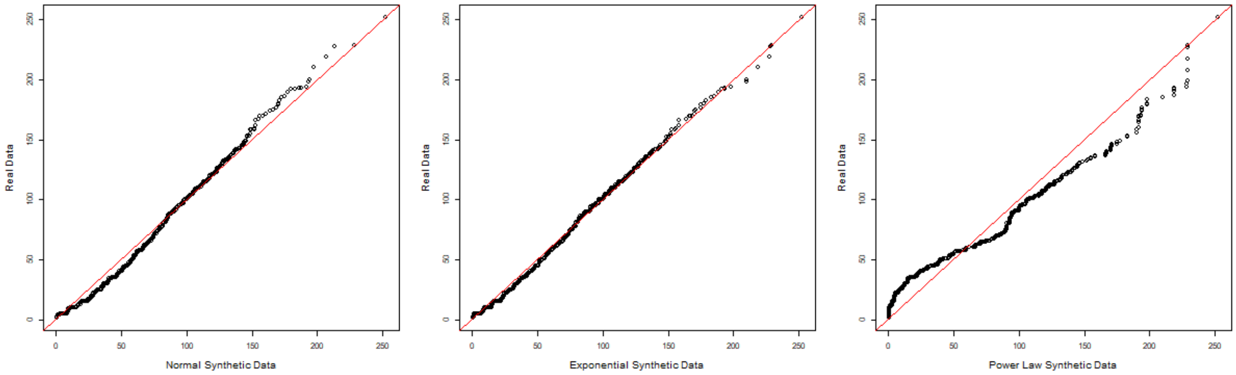

College 30 Cohort 4

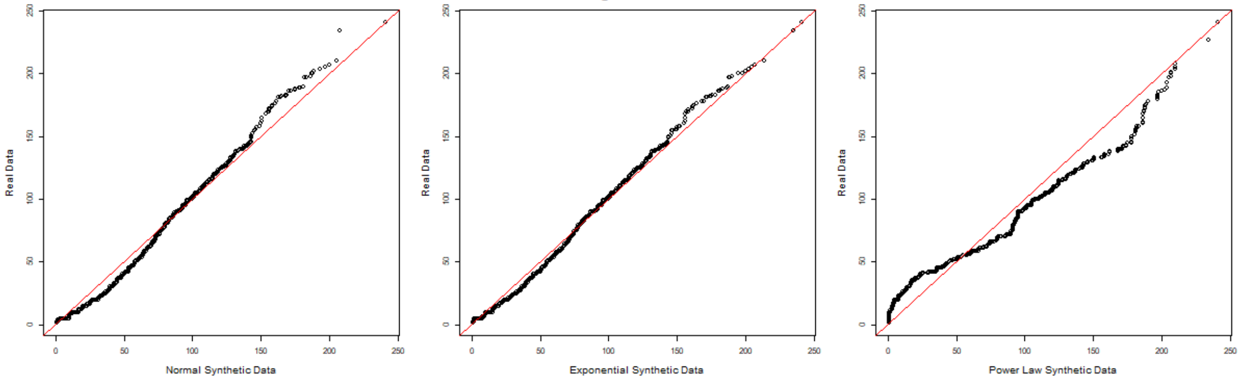

College 30 Cohort 5

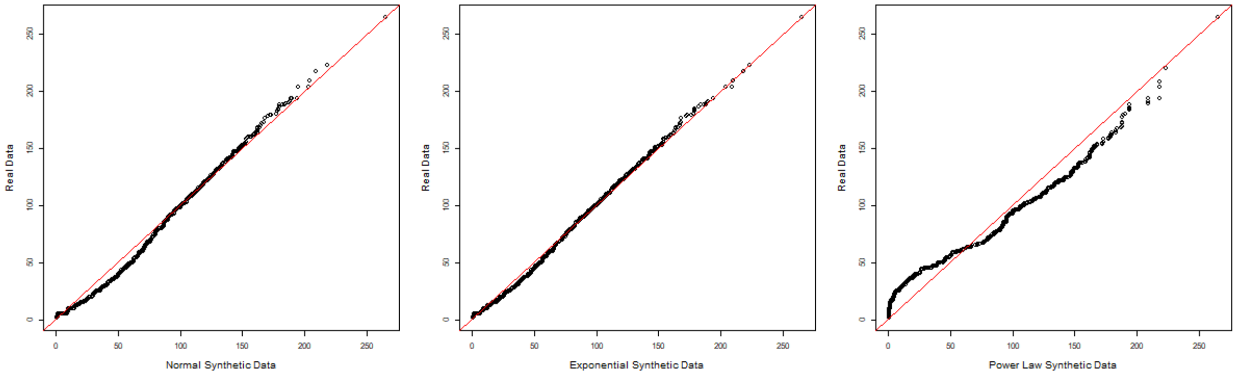

College 40 Cohort 1

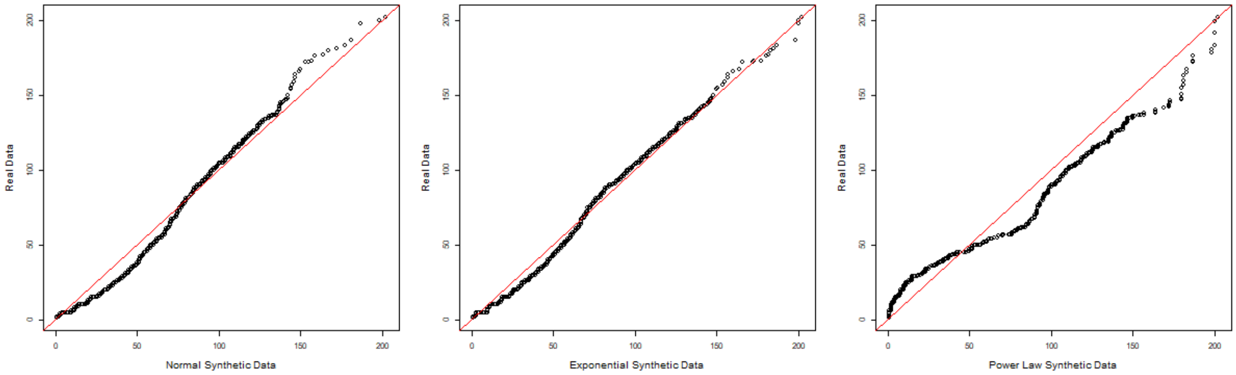

College 40 Cohort 2

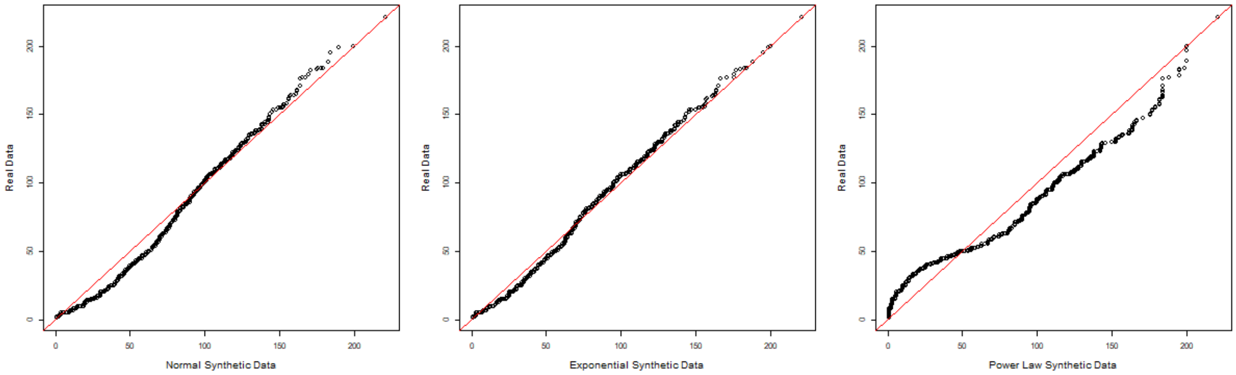

College 40 Cohort 3

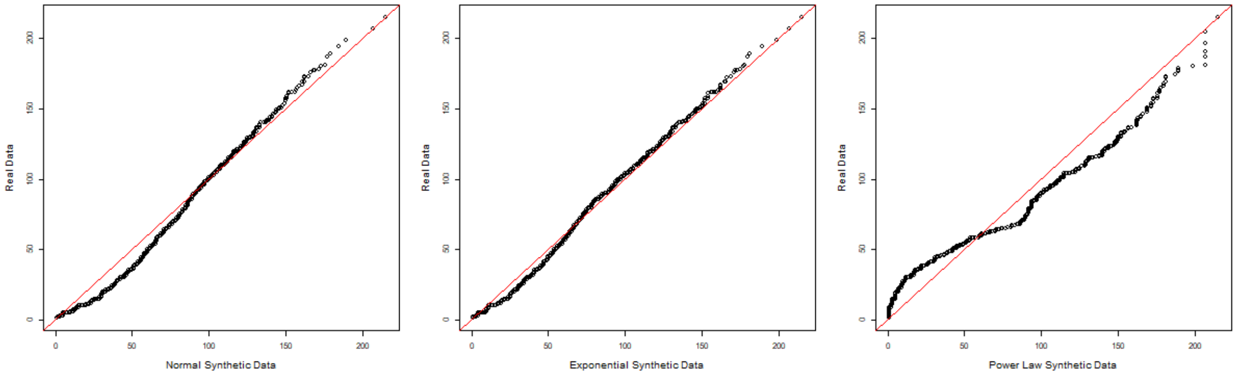

College 40 Cohort 4

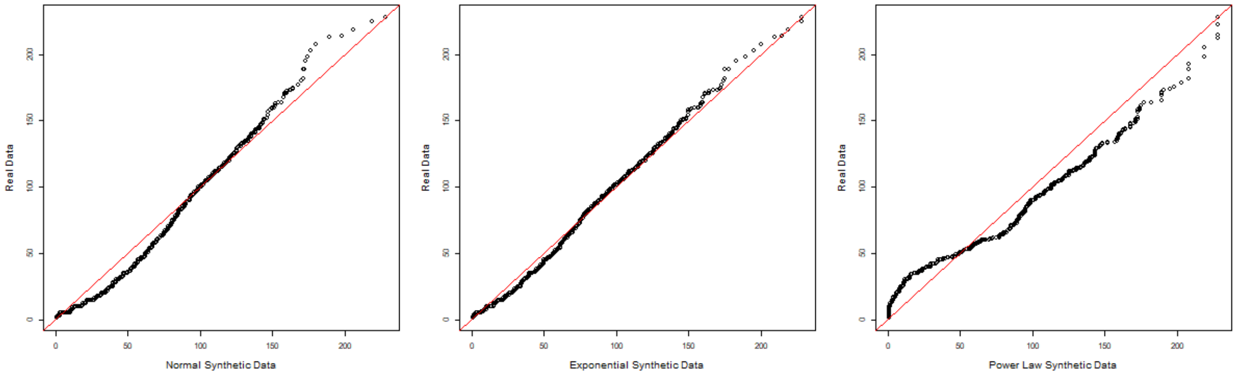

College 40 Cohort 5

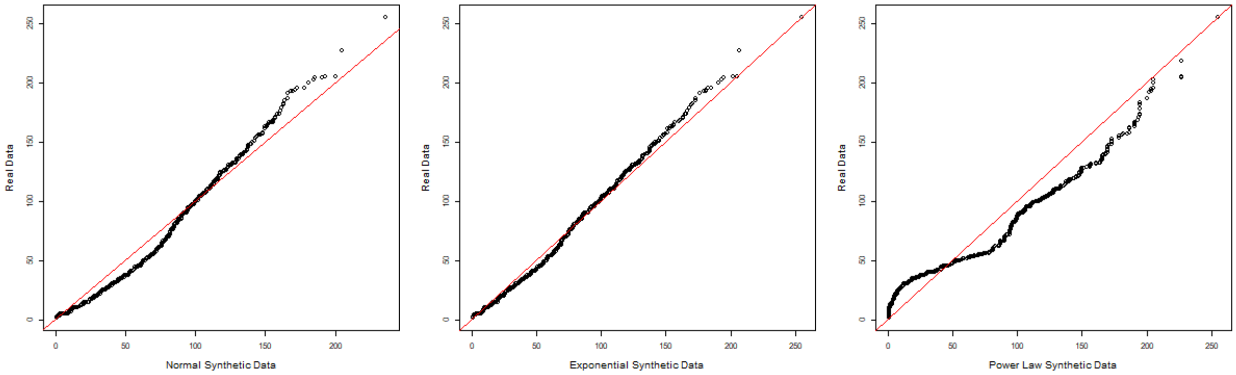

College 50 Cohort 1

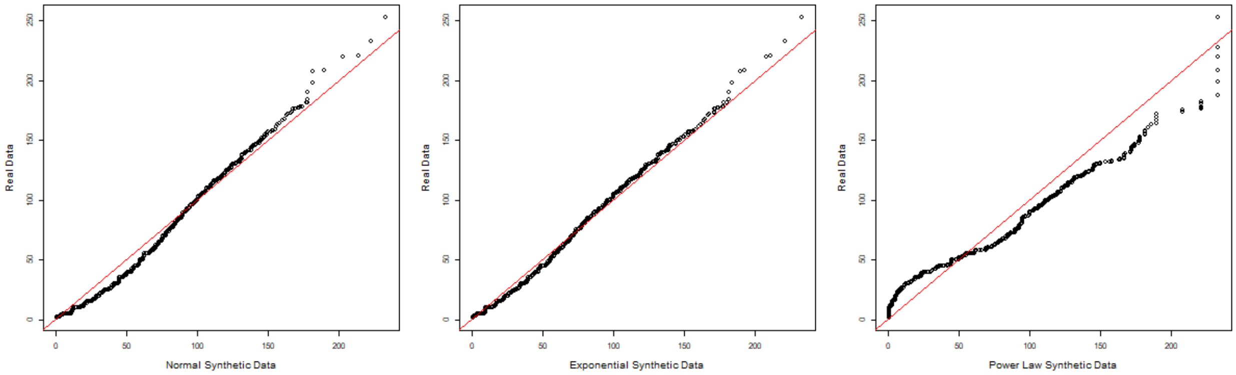

College 50 Cohort 2

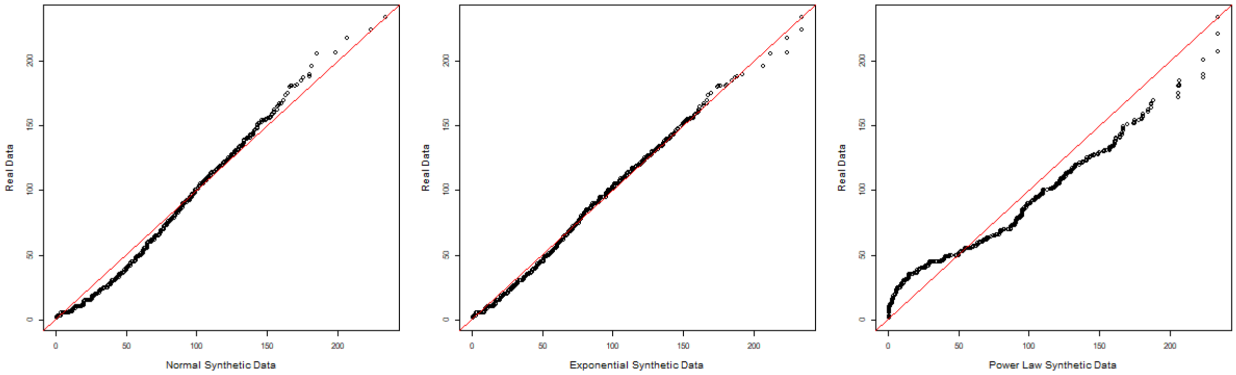

College 50 Cohort 3

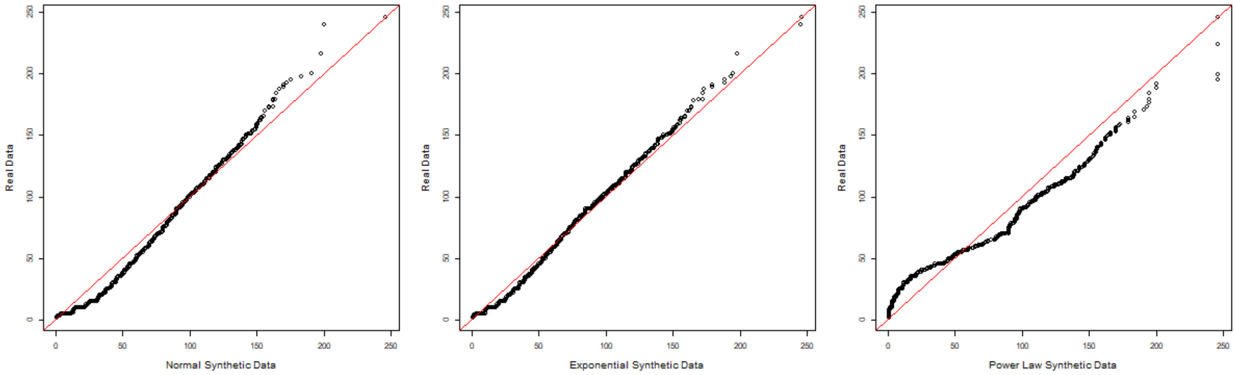

College 50 Cohort 4

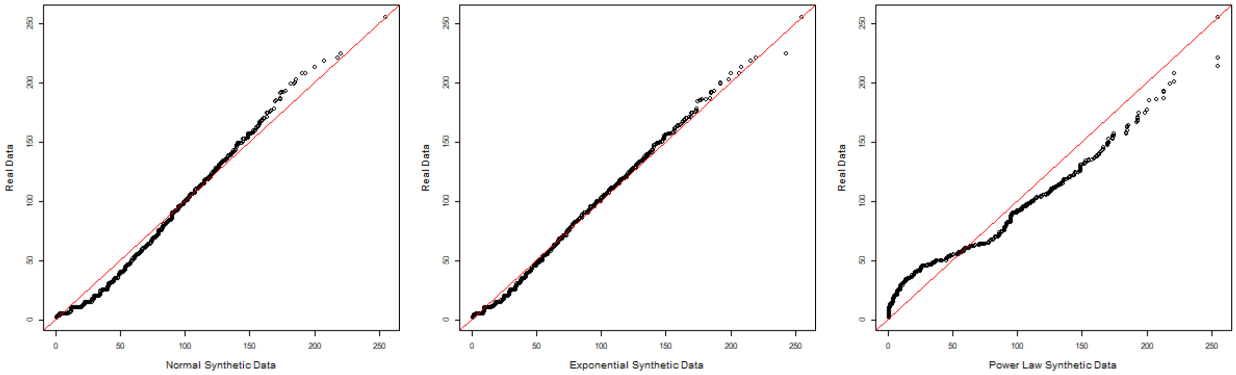

College 50 Cohort 5

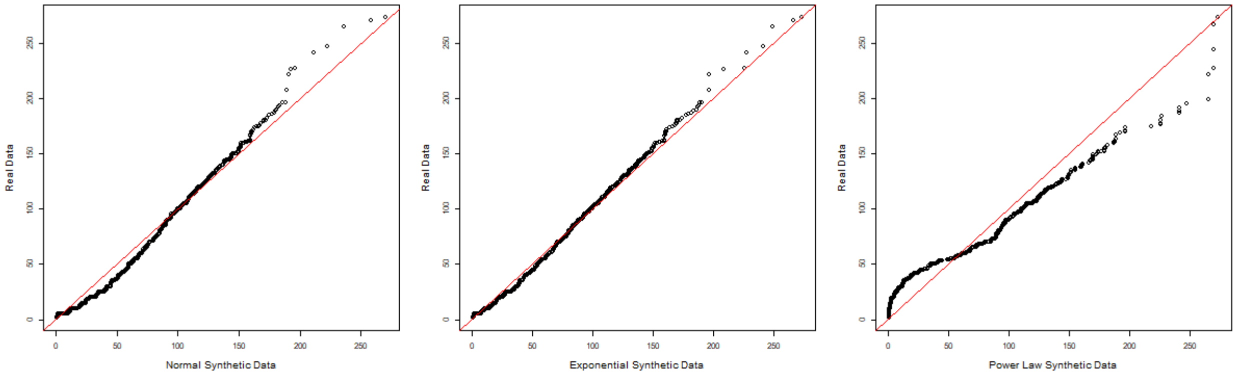

College 62 Cohort 1

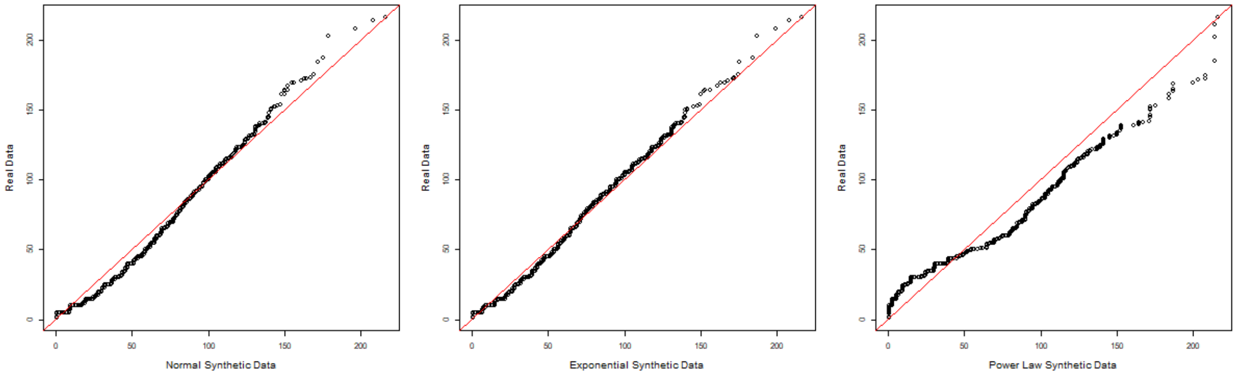

College 62 Cohort 2

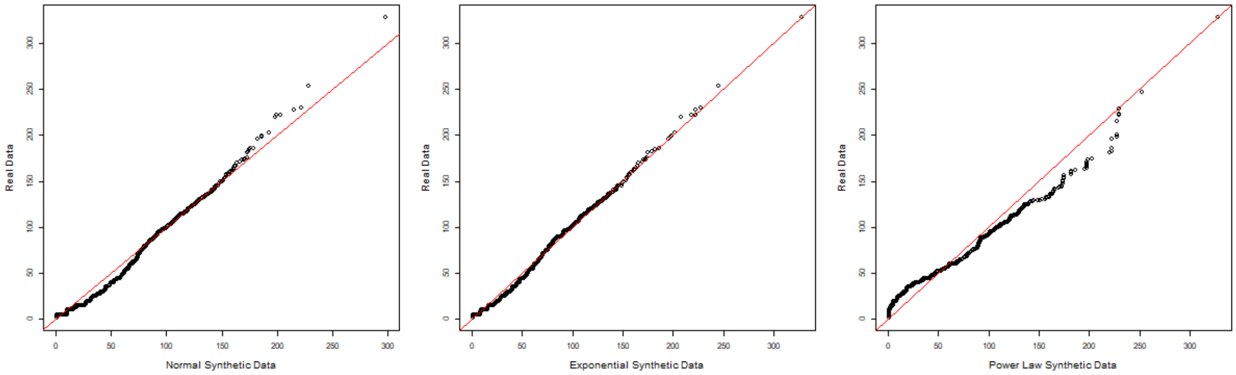

College 62 Cohort 3

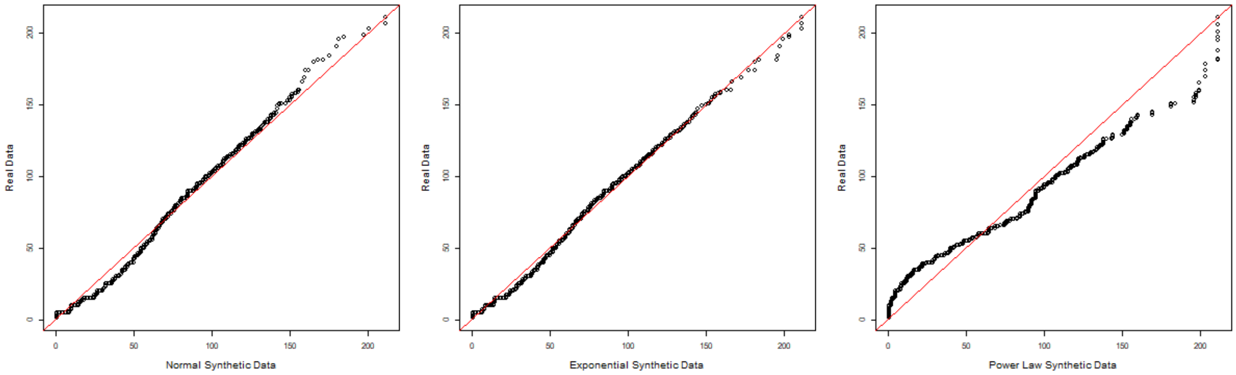

College 62 Cohort 4

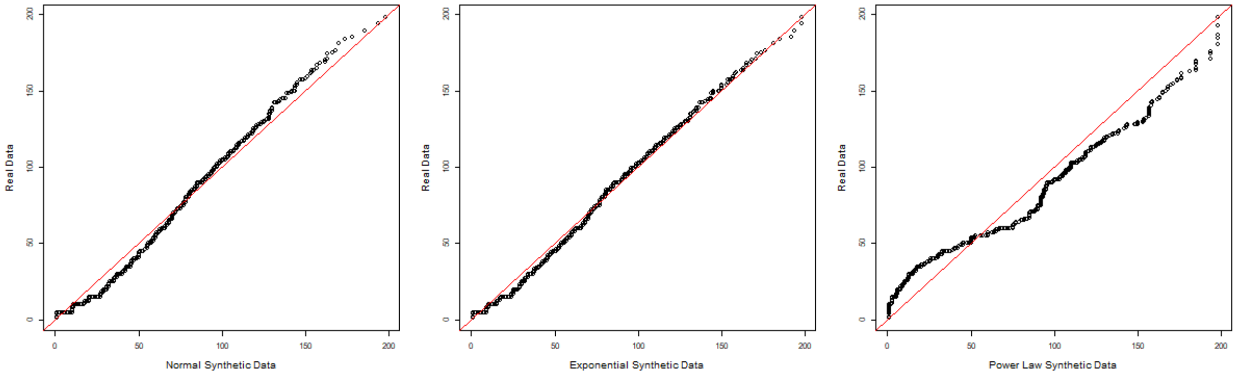

College 62 Cohort 5

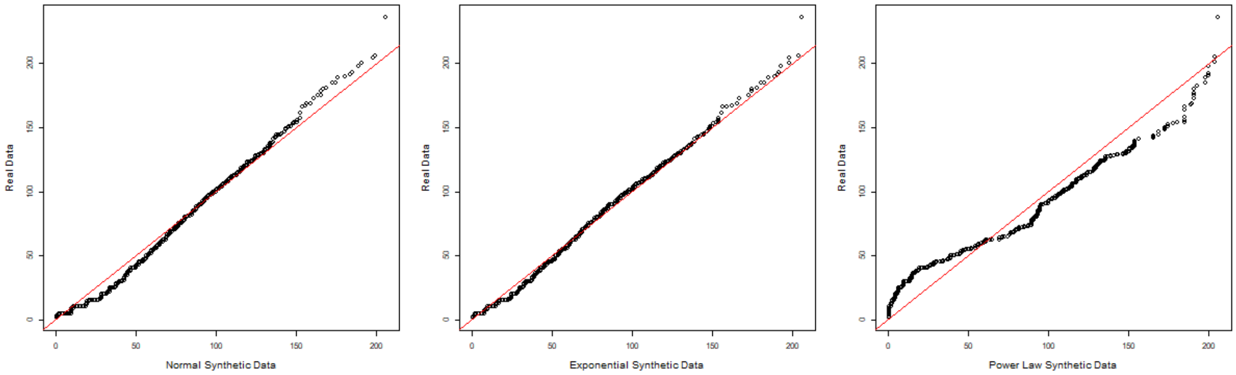

College 63 Cohort 1

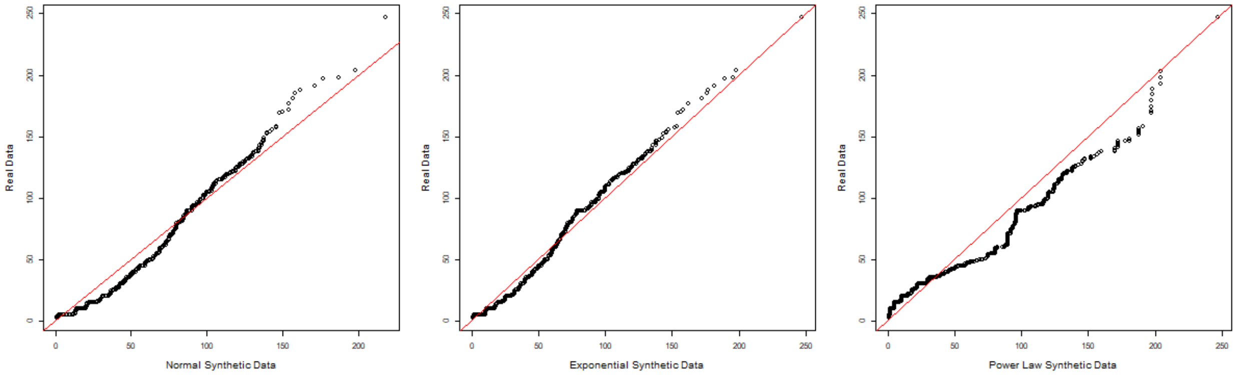

College 63 Cohort 2

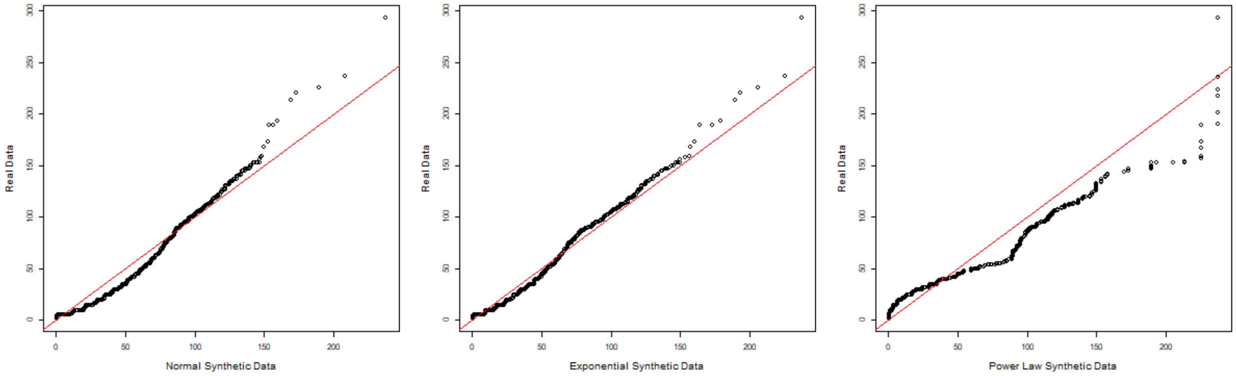

College 63 Cohort 3

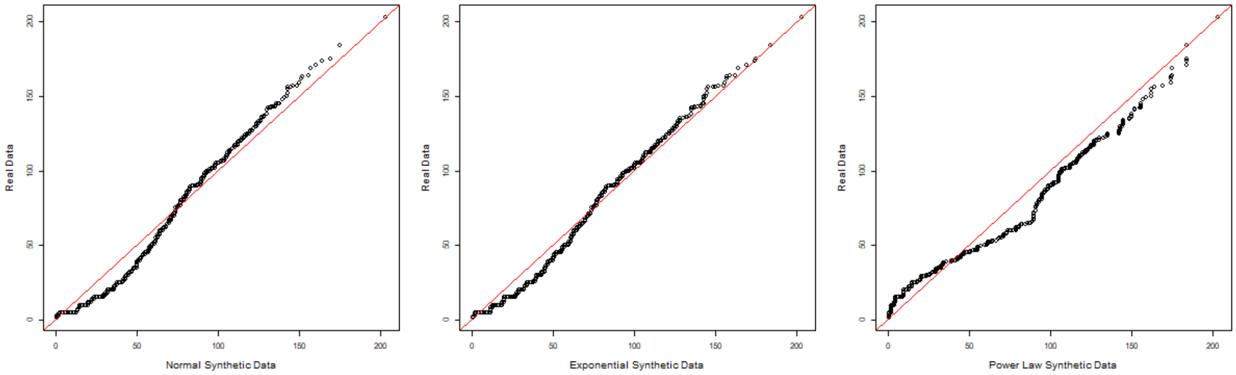

College 63 Cohort 4

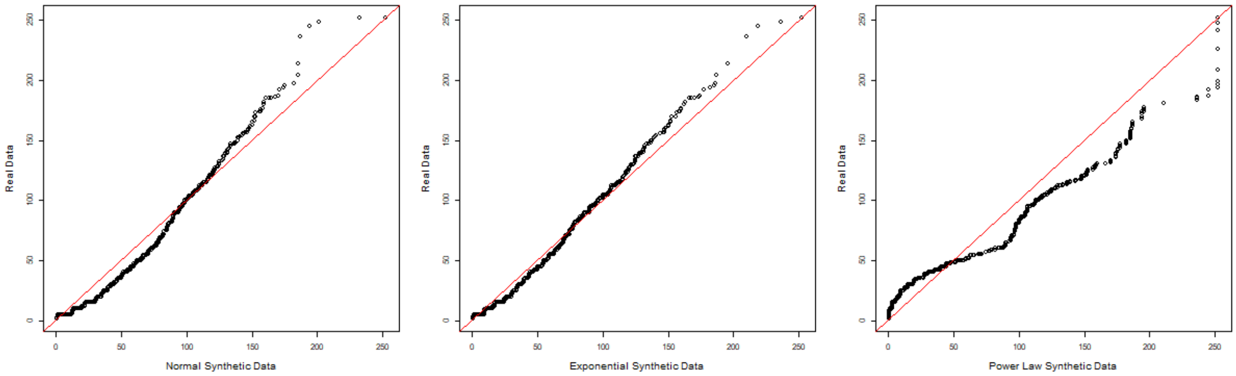

College 63 Cohort 5

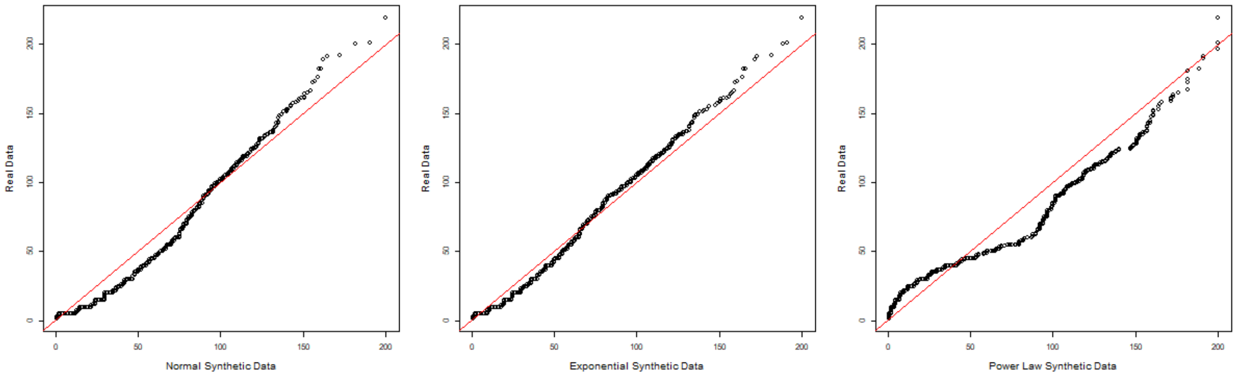

College 64 Cohort 1

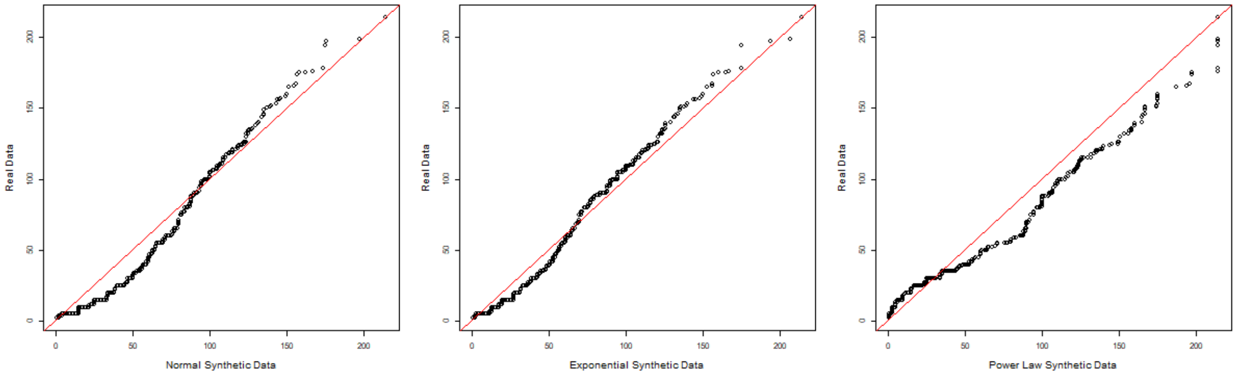

College 64 Cohort 2

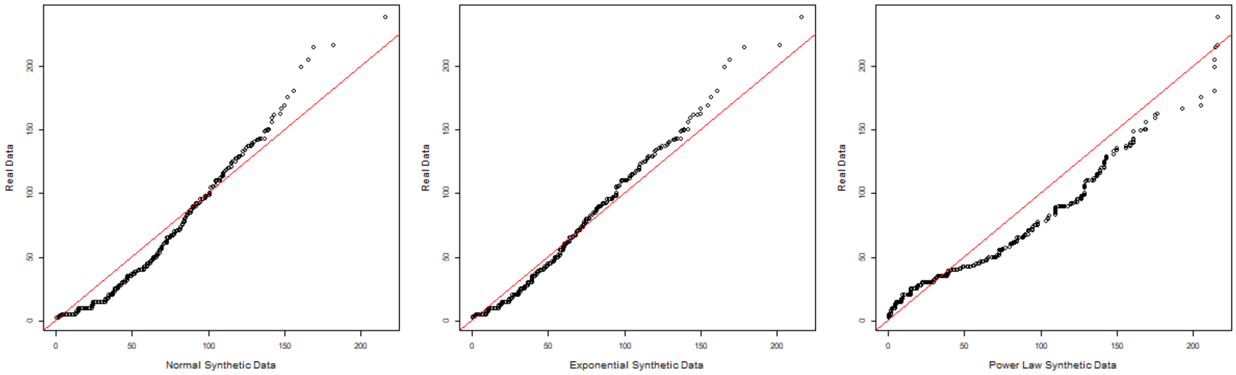

College 64 Cohort 3

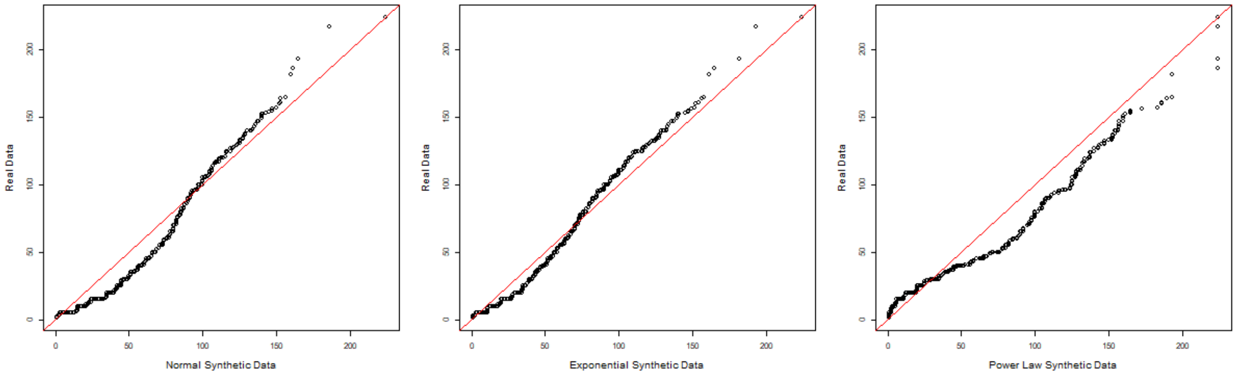

College 64 Cohort 4

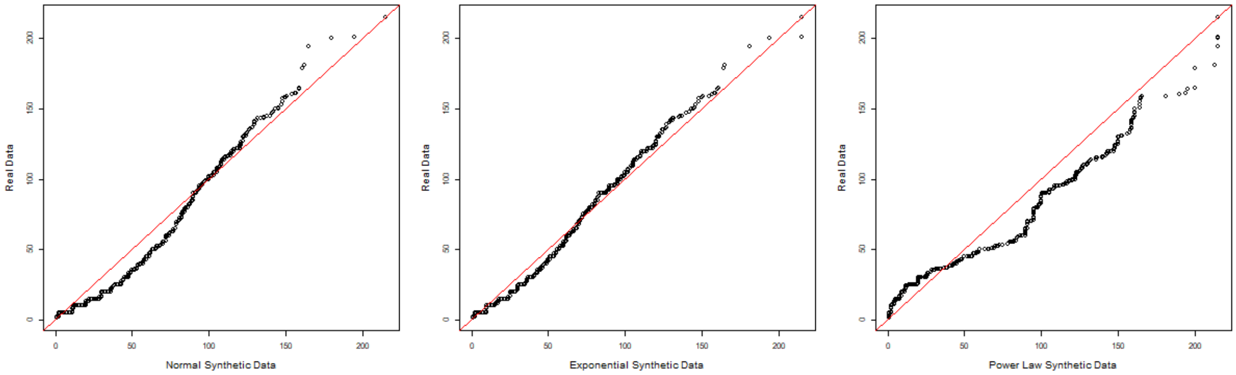

College 64 Cohort 5

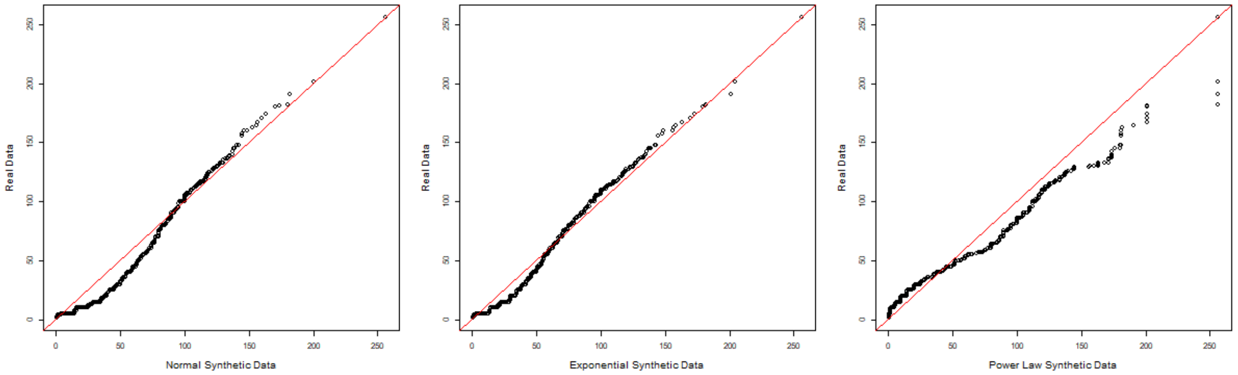

College 70 Cohort 1

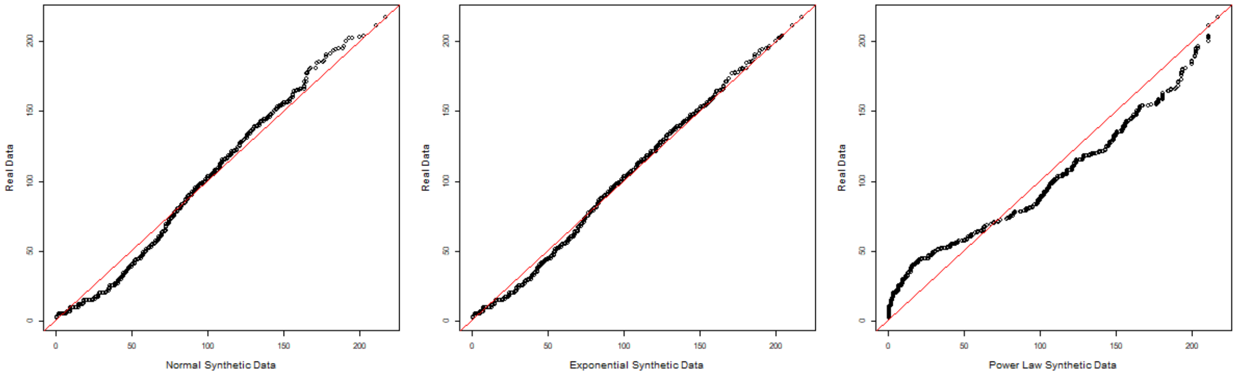

College 70 Cohort 2

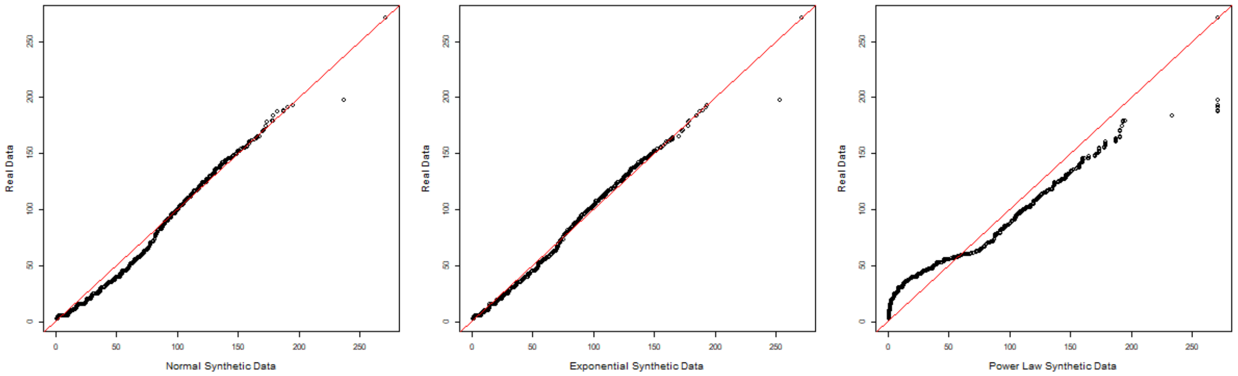

College 70 Cohort 3

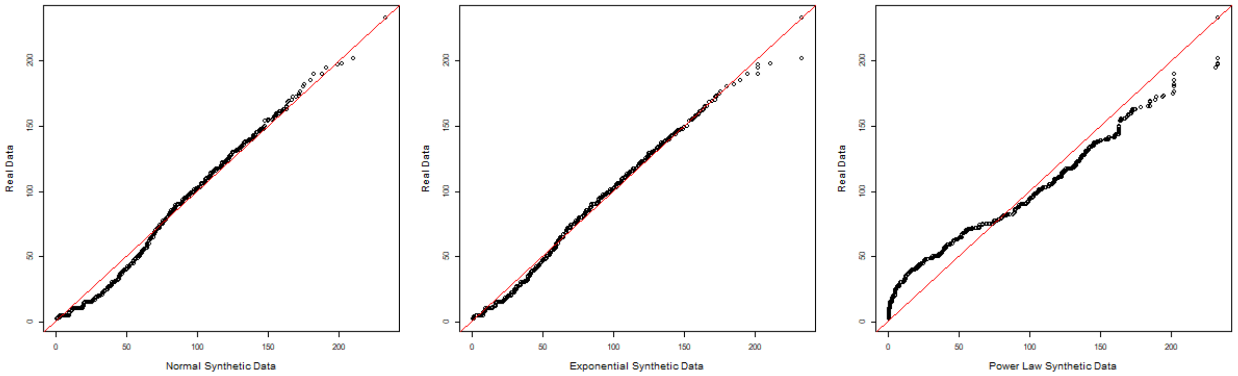

College 70 Cohort 4

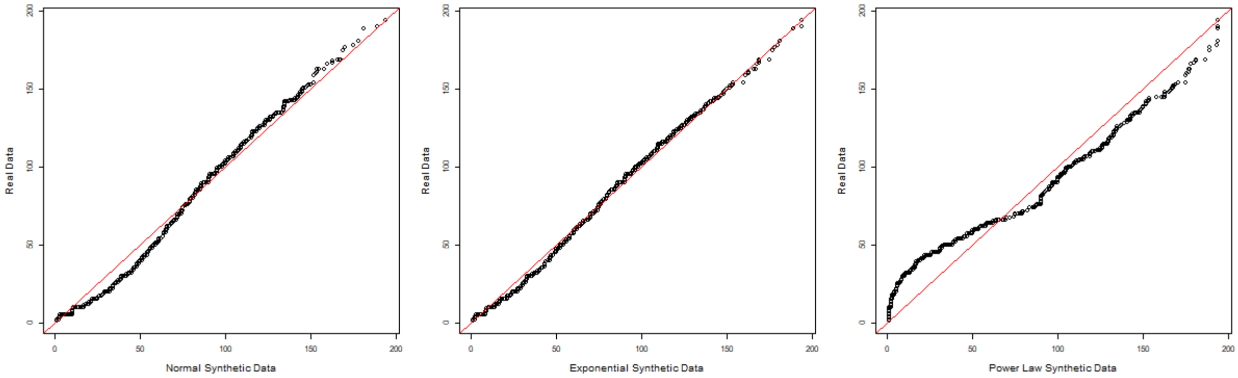

College 70 Cohort 5

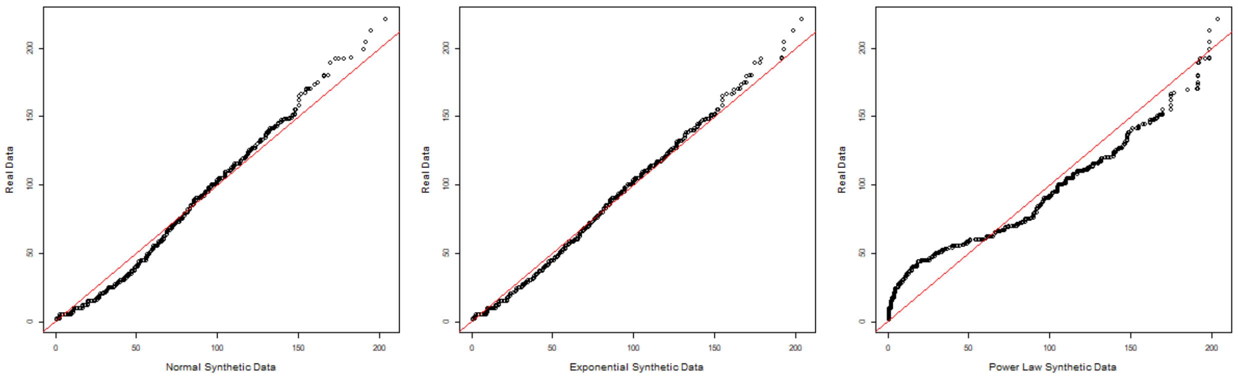

College 90 Cohort 1

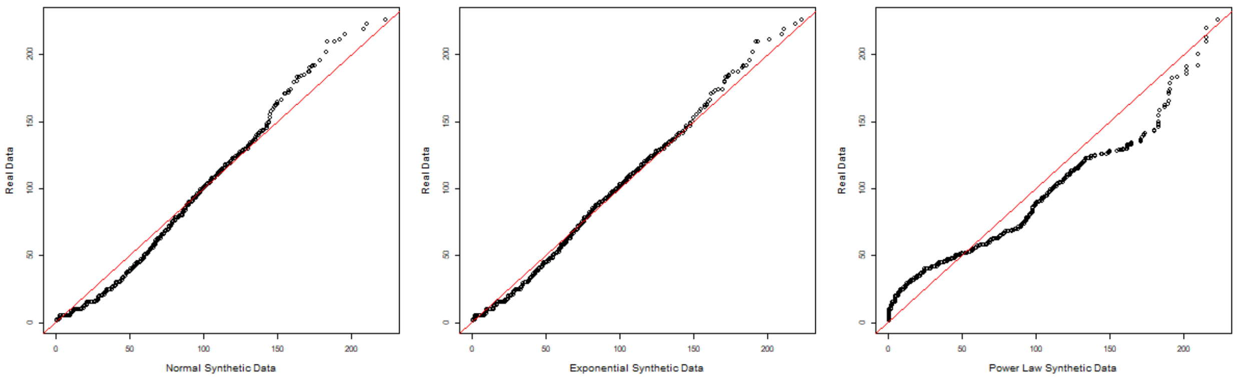

College 90 Cohort 2

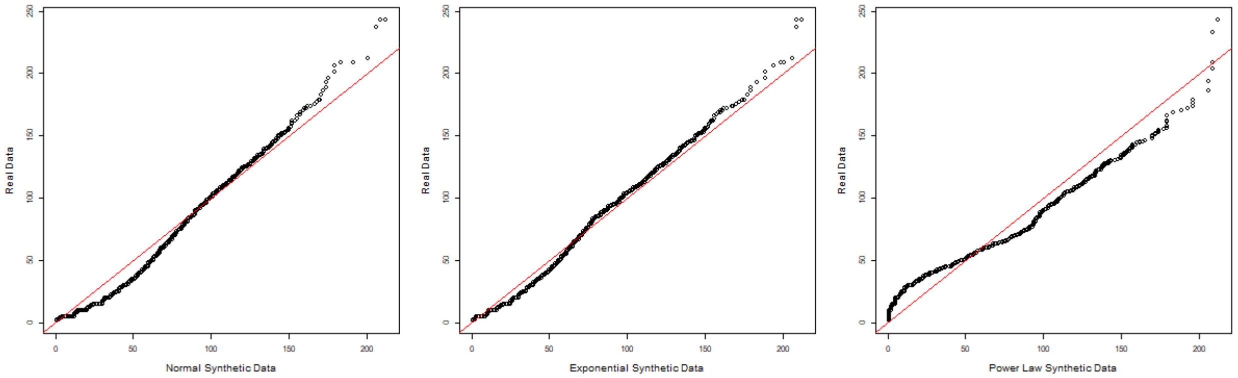

College 90 Cohort 3

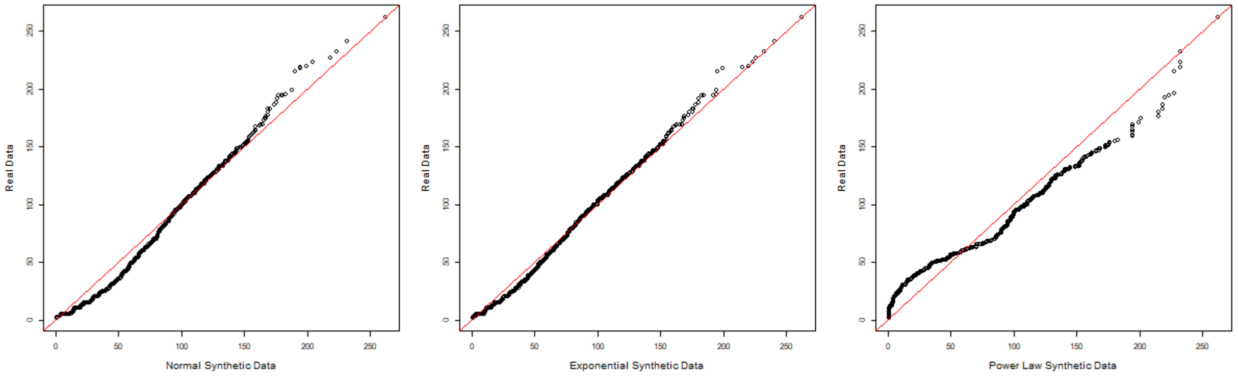

College 90 Cohort 4

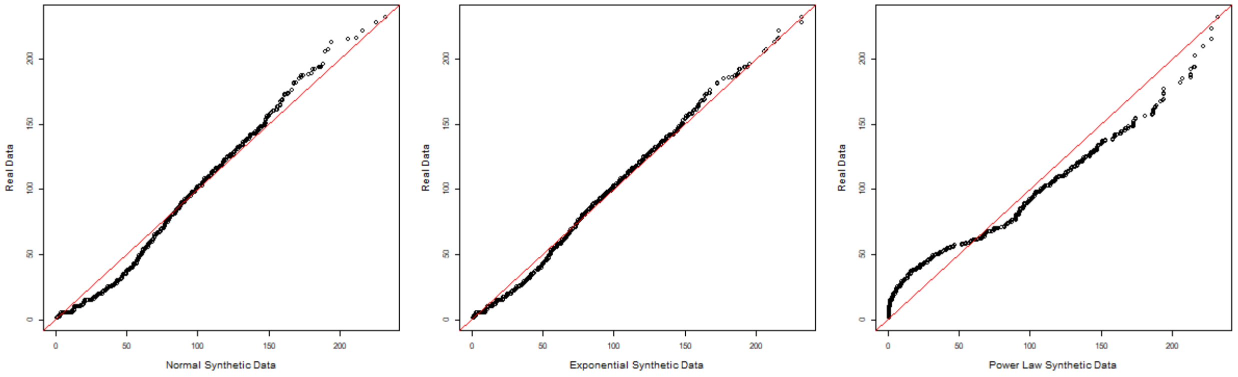

College 90 Cohort 5

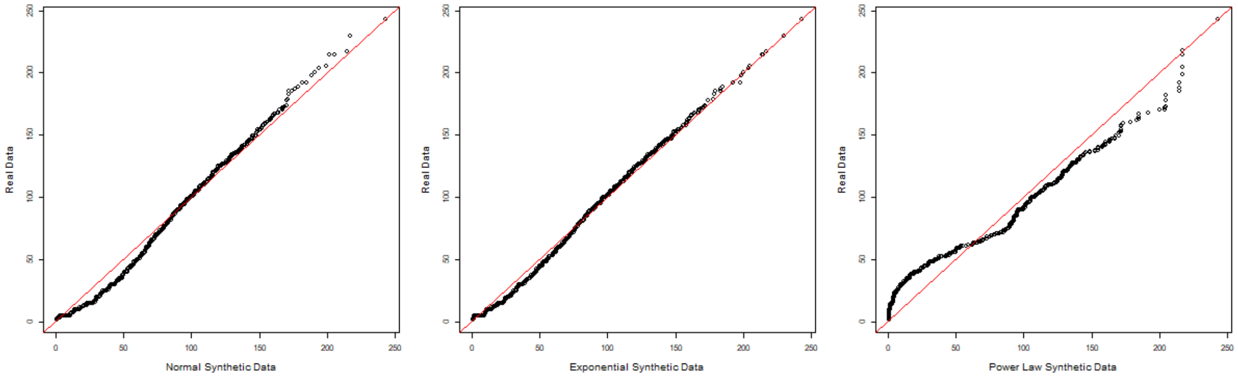

College 100 Cohort 1

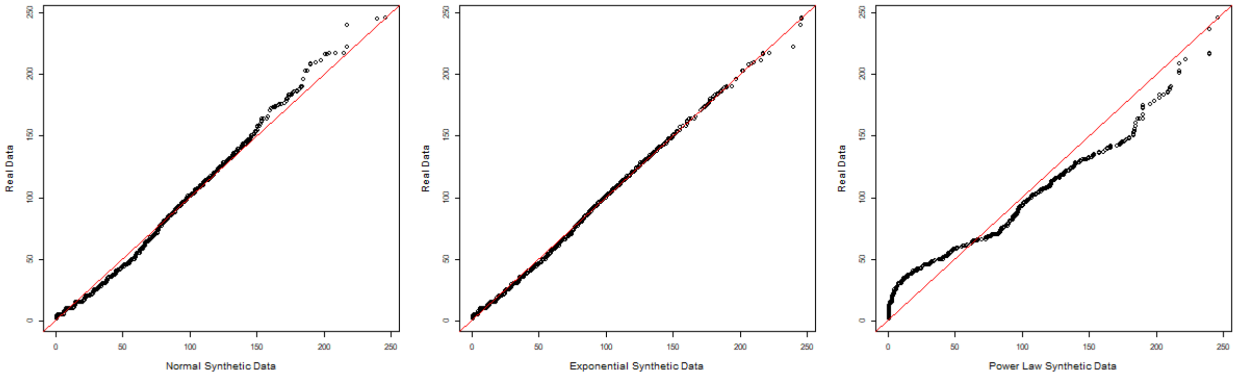

College 100 Cohort 2

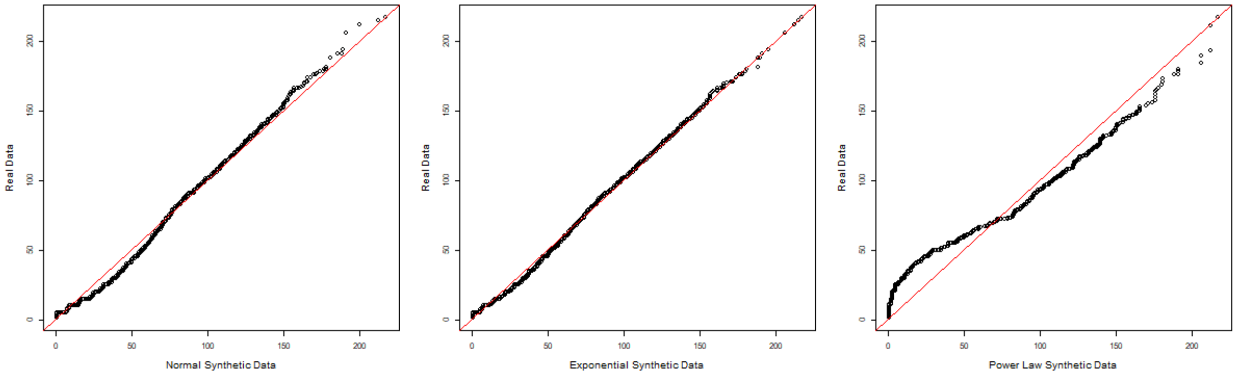

College 100 Cohort 3

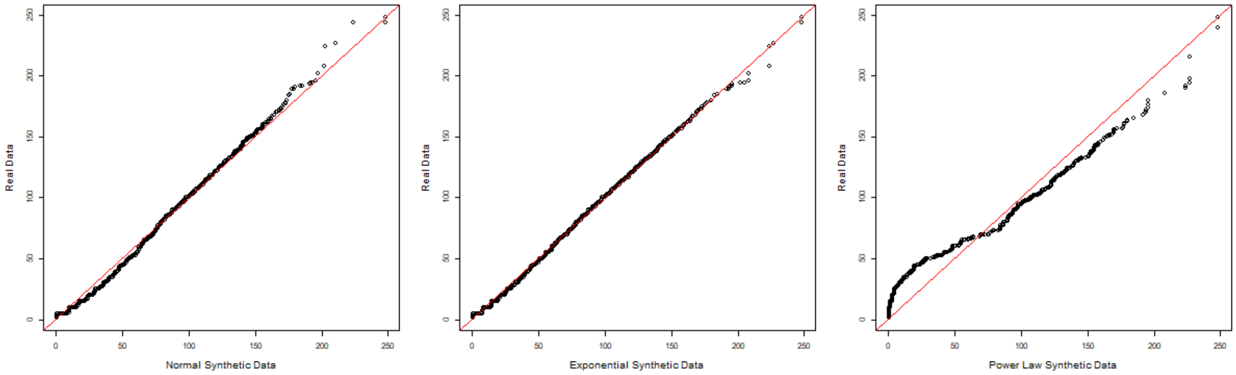

College 100 Cohort 4

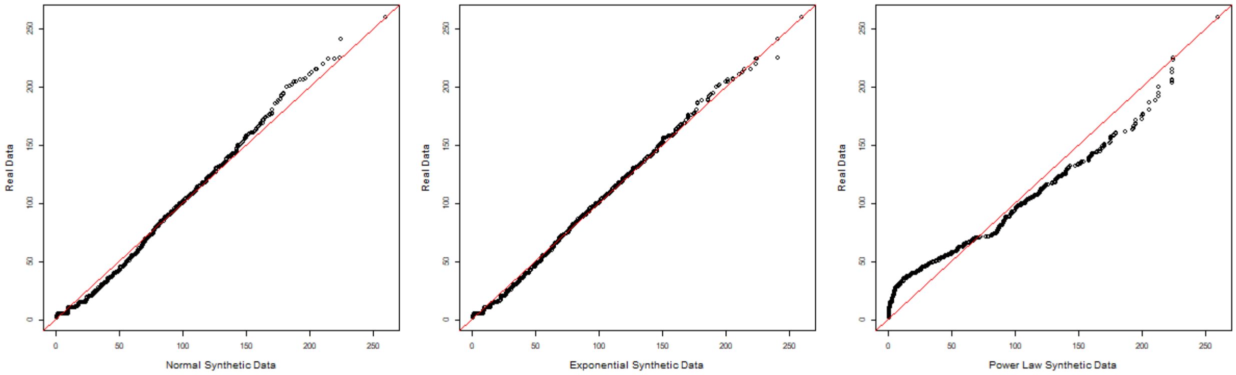

College 100 Cohort 5

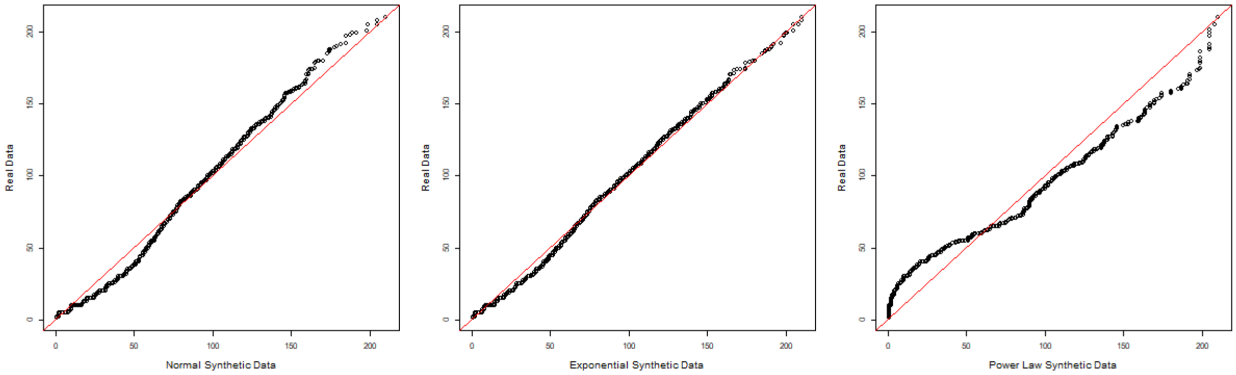

College 111 Cohort 1

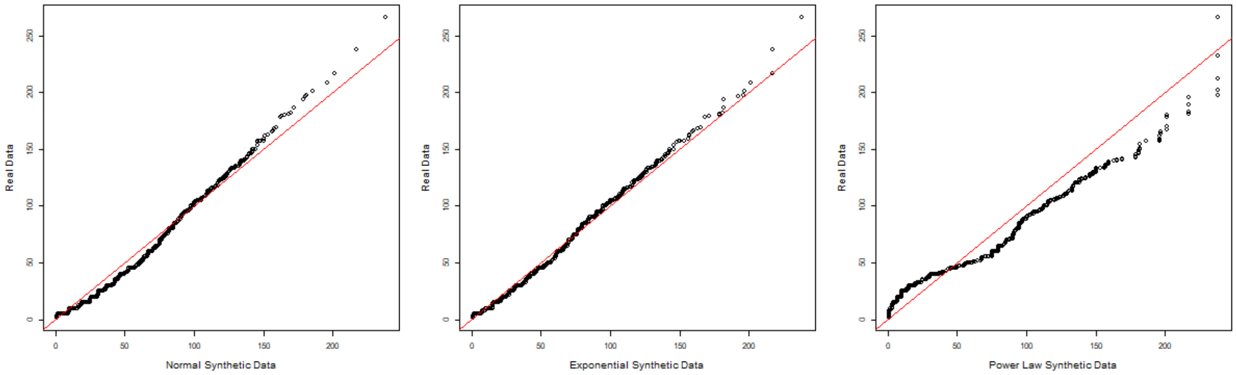

College 111 Cohort 2

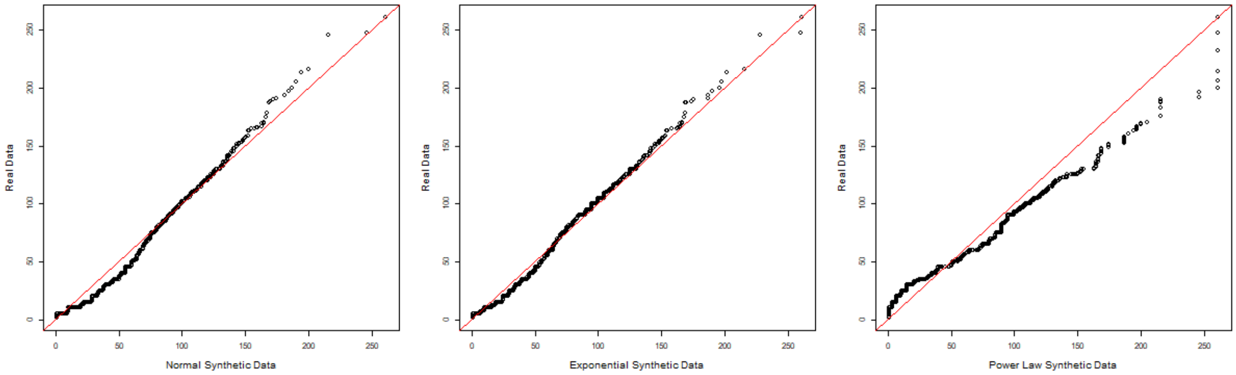

College 111 Cohort 3

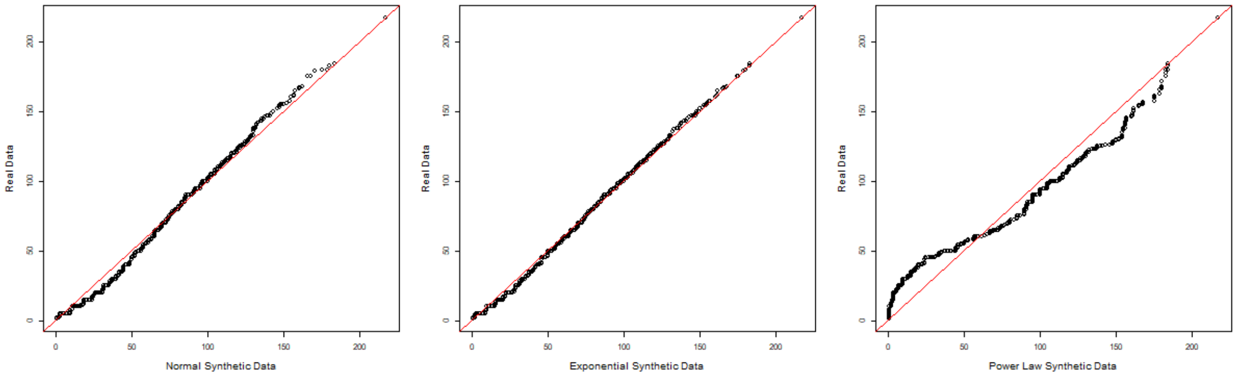

College 111 Cohort 4

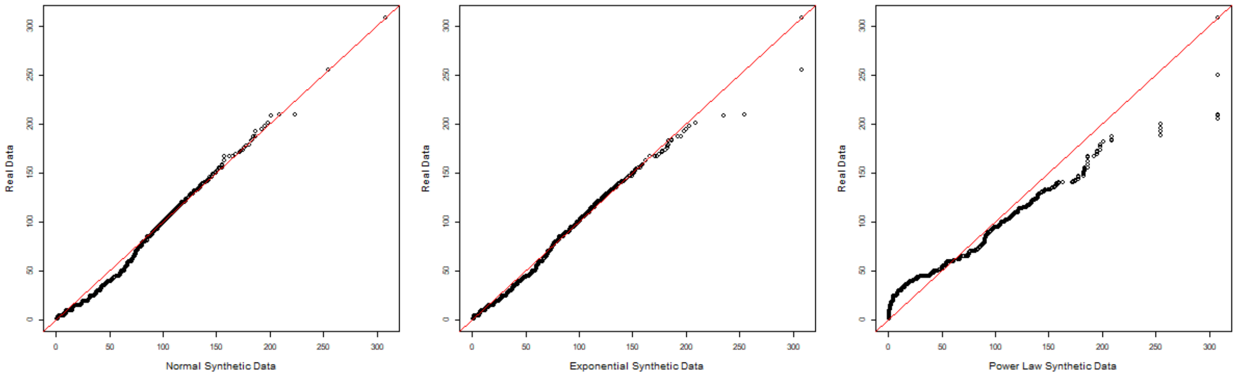

College 111 Cohort 5

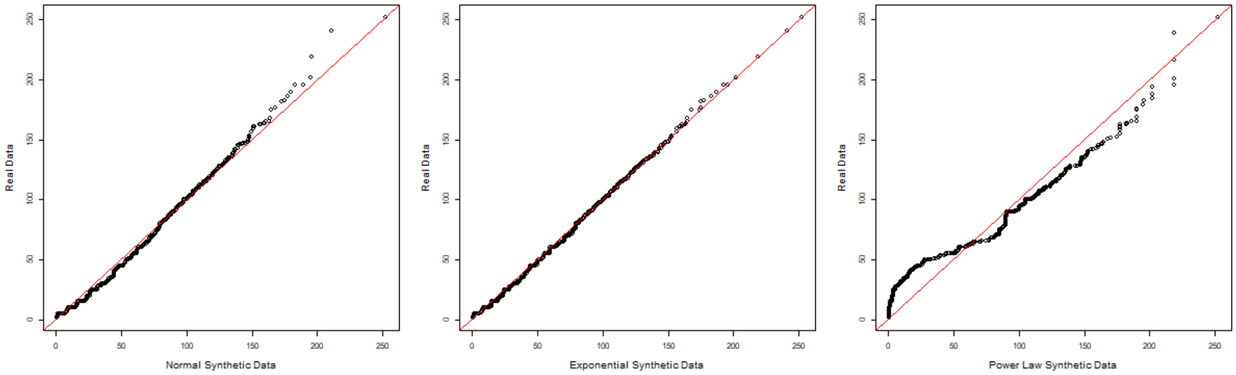

College 112 Cohort 1

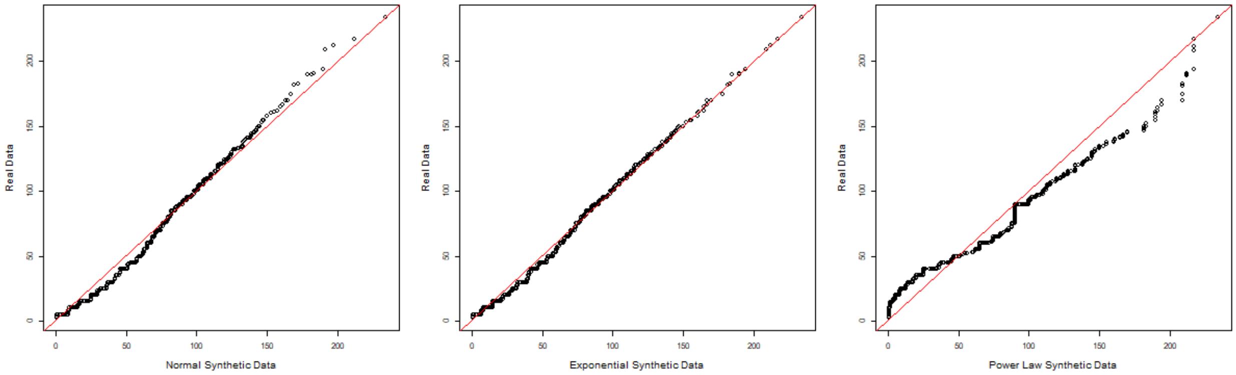

College 112 Cohort 2

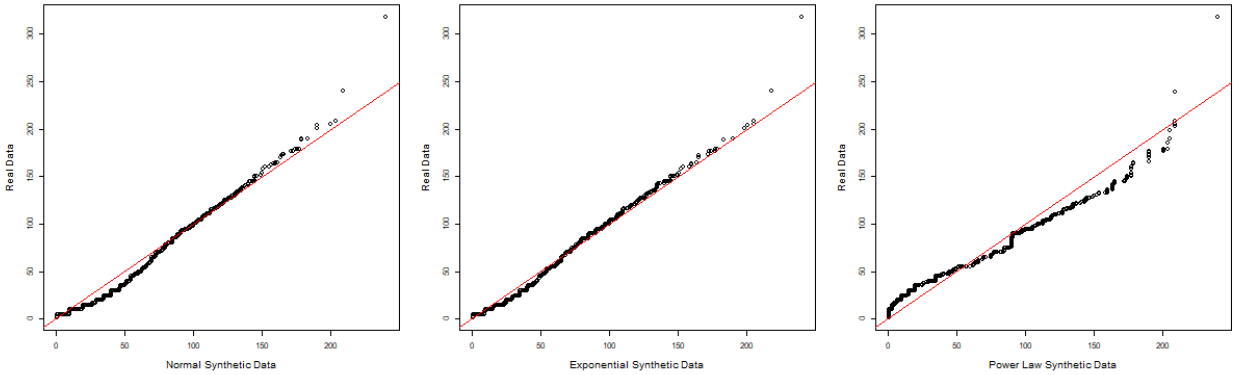

College 112 Cohort 3

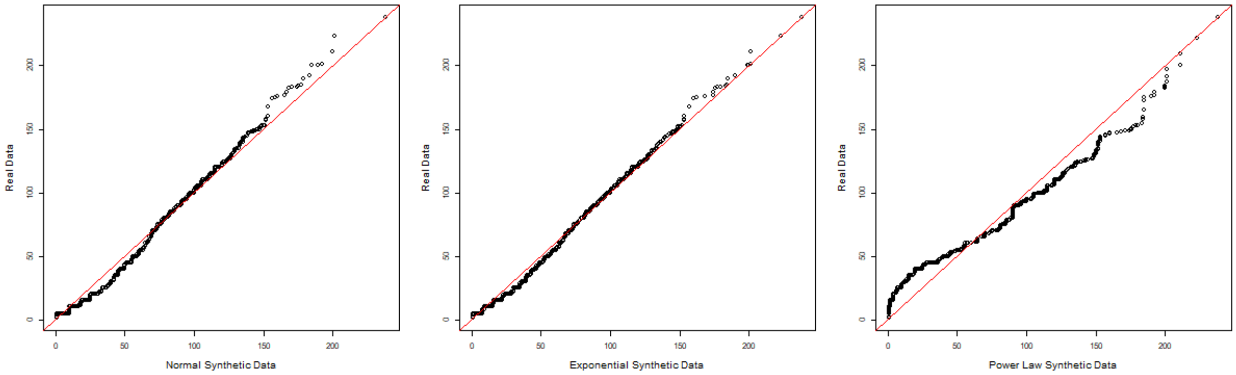

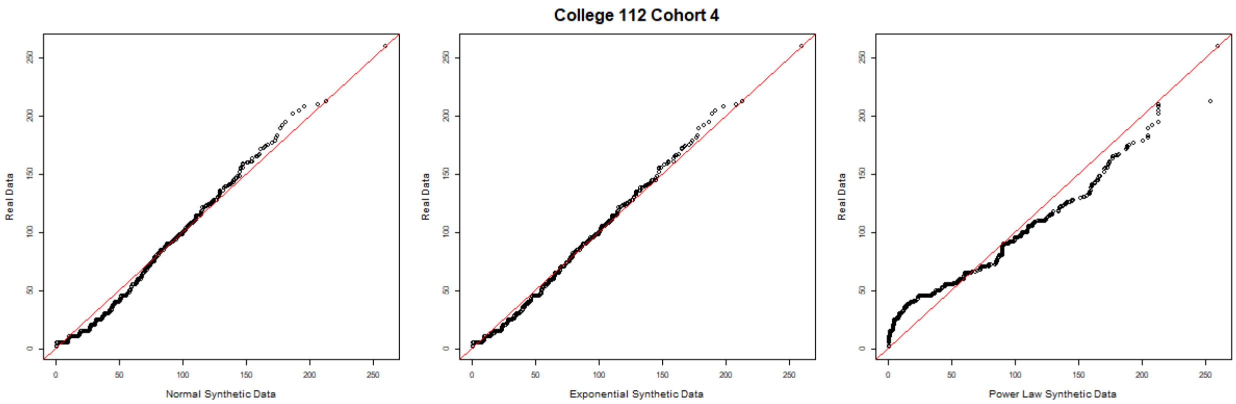
College 112 Cohort 4
Real Data
Normal Synthetic Data
Exponential Synthetic Data
Power Law Synthetic Data

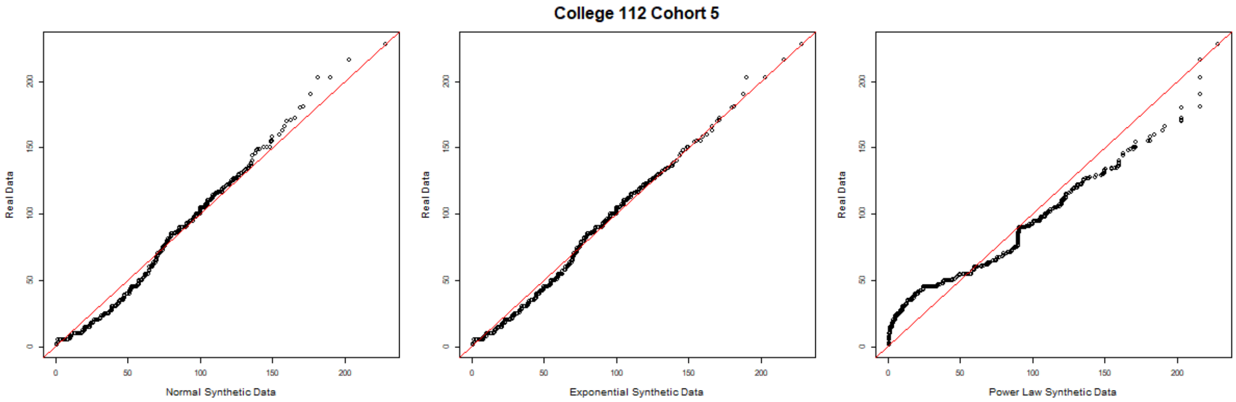
College 112 Cohort 5
Real Data
Normal Synthetic Data
Exponential Synthetic Data
Power Law Synthetic Data

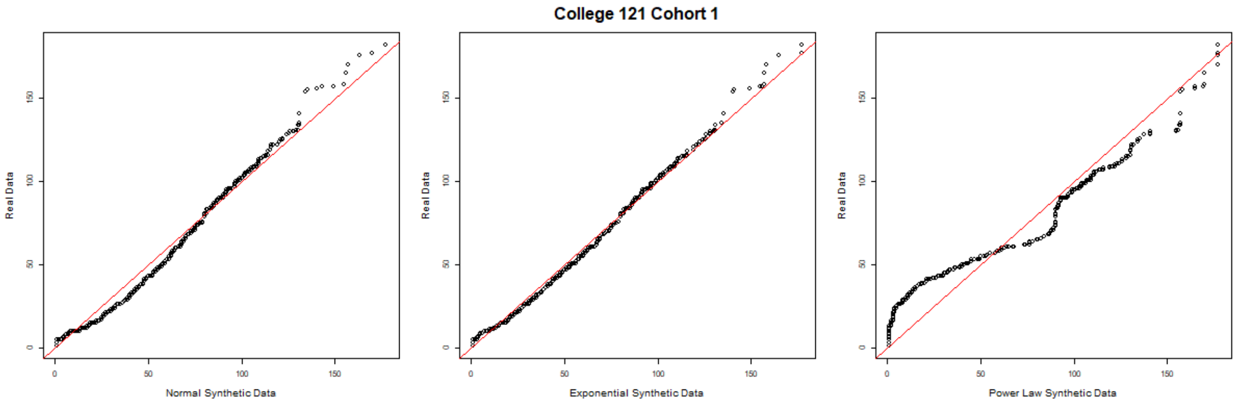
College 121 Cohort 1
Real Data
Normal Synthetic Data
Exponential Synthetic Data
Power Law Synthetic Data

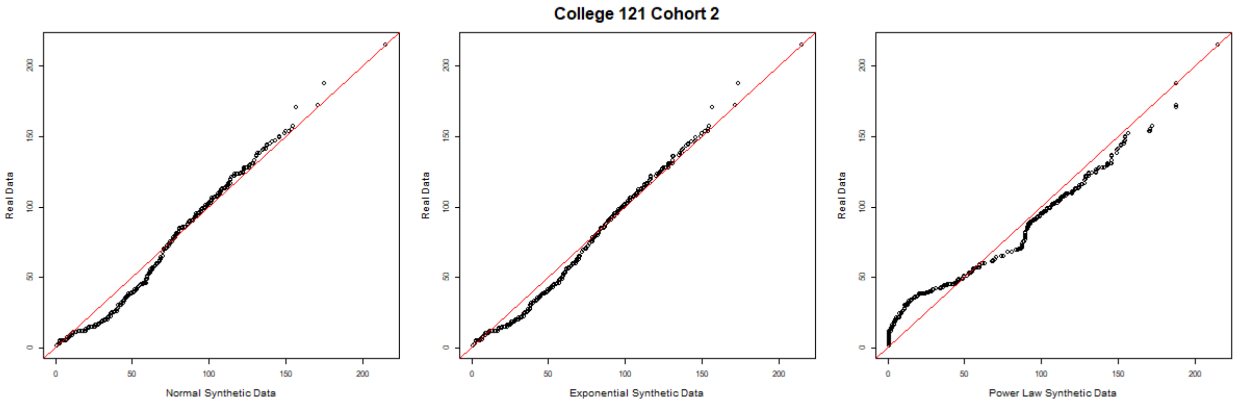
College 121 Cohort 2
Real Data
Normal Synthetic Data
Exponential Synthetic Data
Power Law Synthetic Data

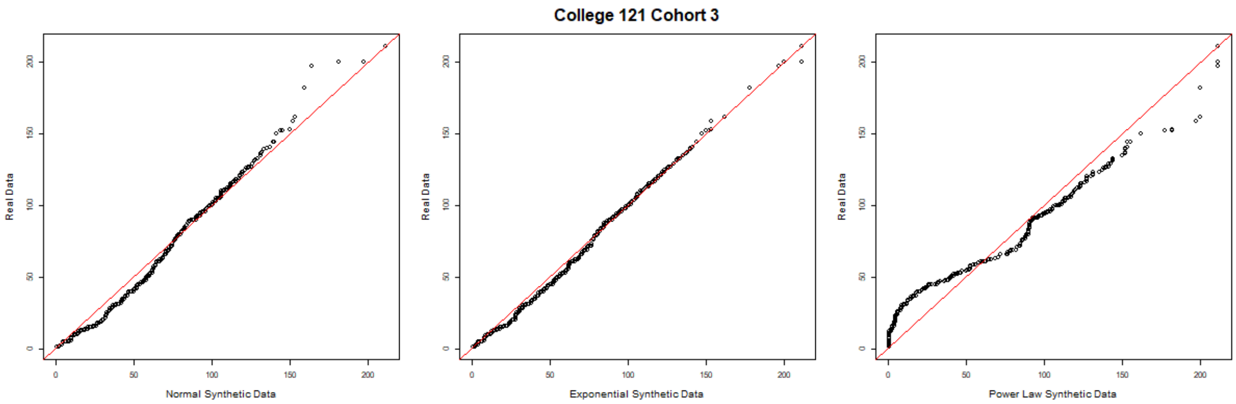
College 121 Cohort 3
Real Data
Normal Synthetic Data
Exponential Synthetic Data
Power Law Synthetic Data

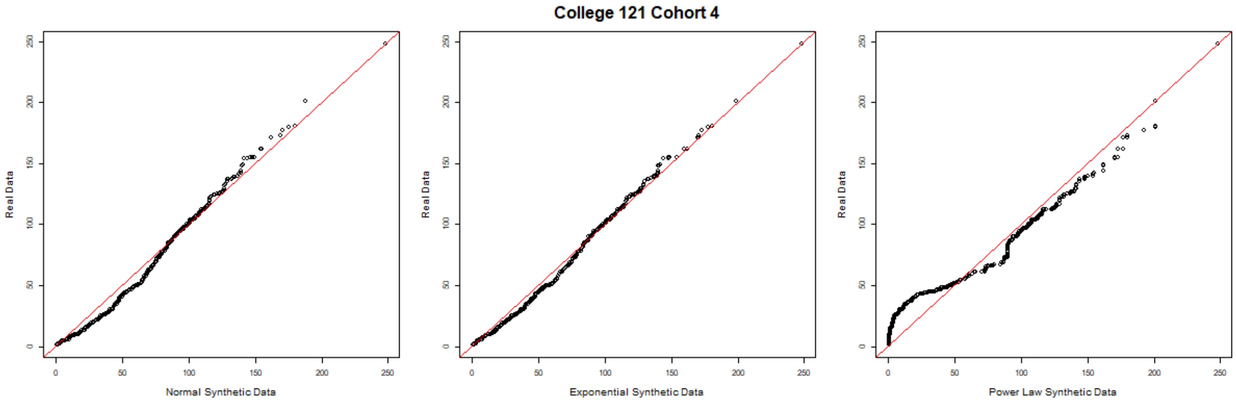
College 121 Cohort 4
Real Data
Normal Synthetic Data
Exponential Synthetic Data
Power Law Synthetic Data

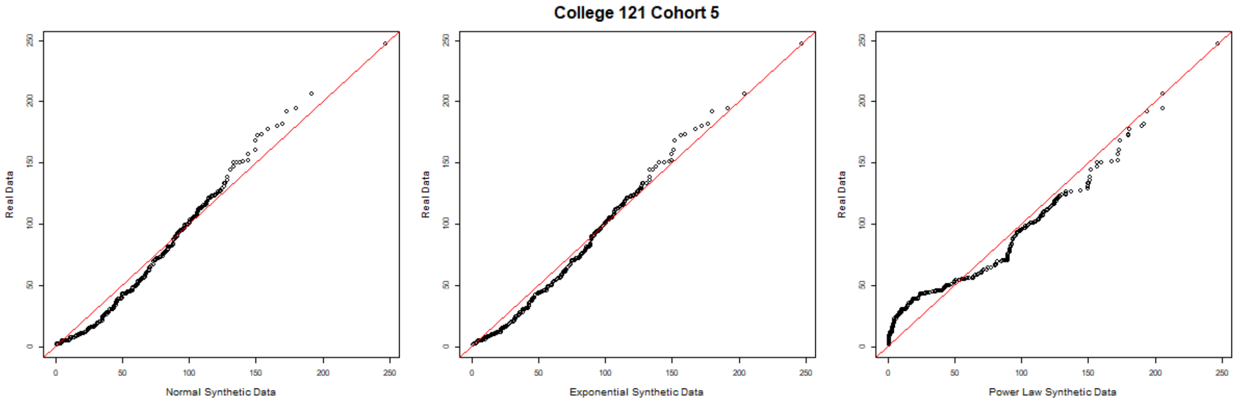
College 121 Cohort 5
Real Data
Normal Synthetic Data
Exponential Synthetic Data
Power Law Synthetic Data

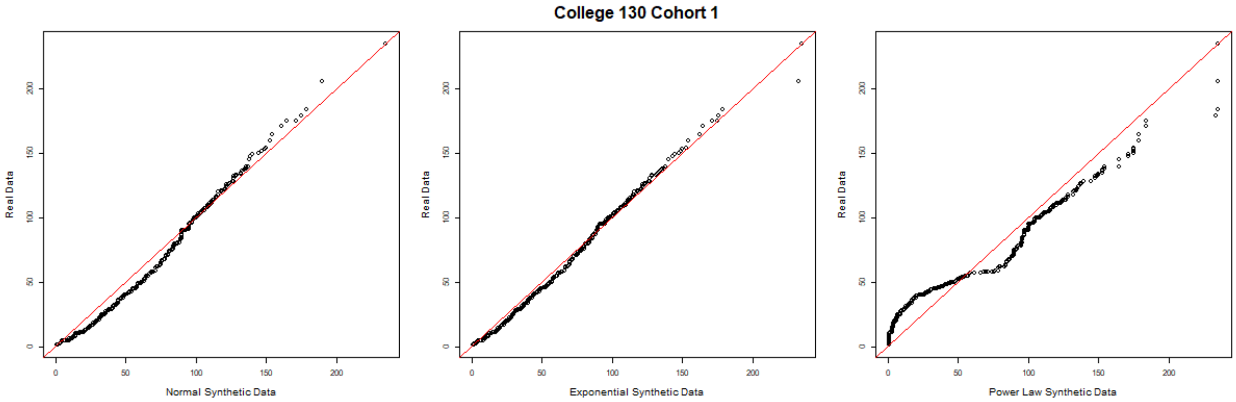
College 130 Cohort 1
Real Data
Normal Synthetic Data
Exponential Synthetic Data
Power Law Synthetic Data

College 130 Cohort 2

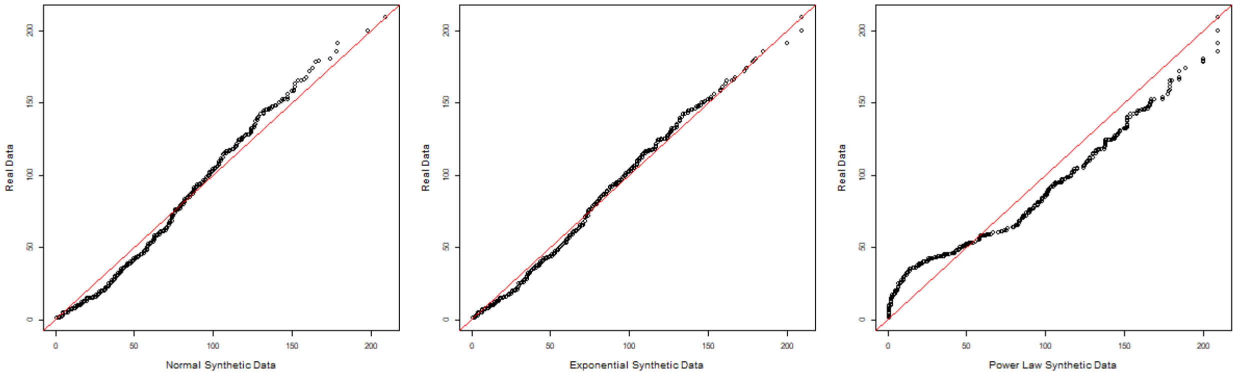

College 130 Cohort 3

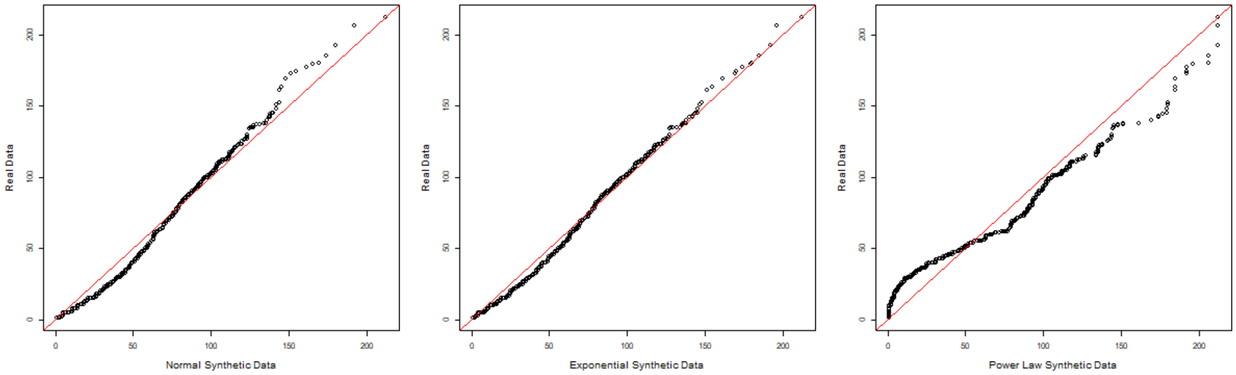

College 130 Cohort 4

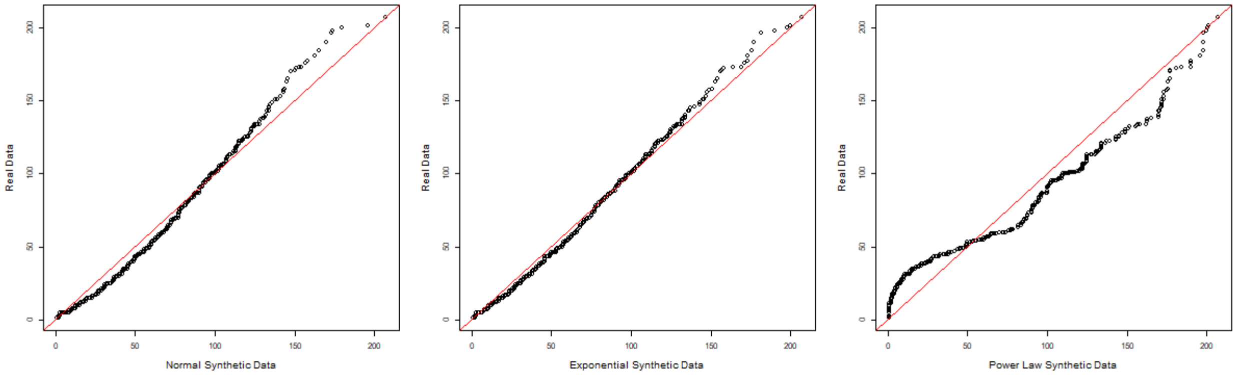

College 130 Cohort 5

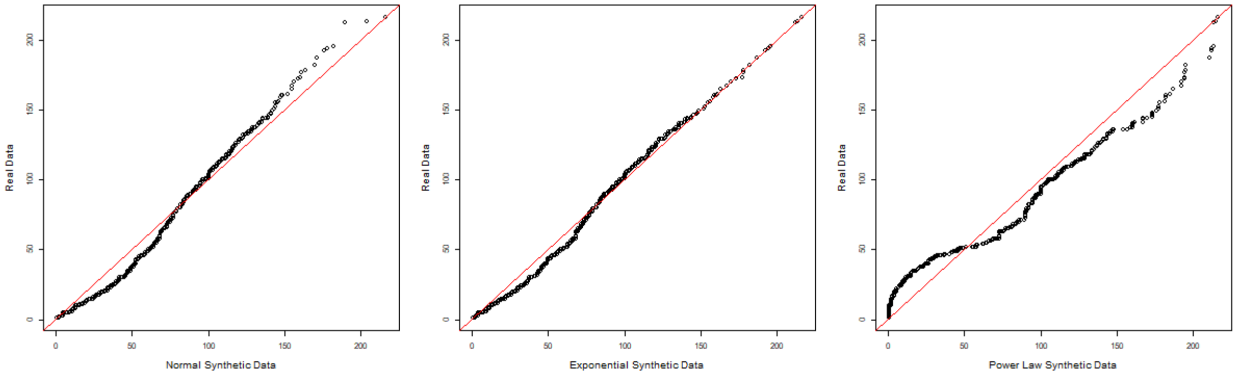

College 140 Cohort 1

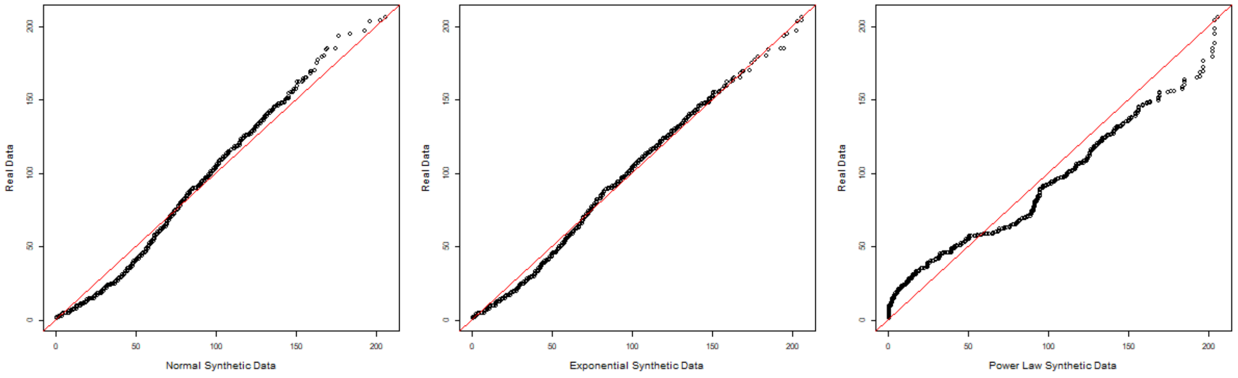

College 140 Cohort 2

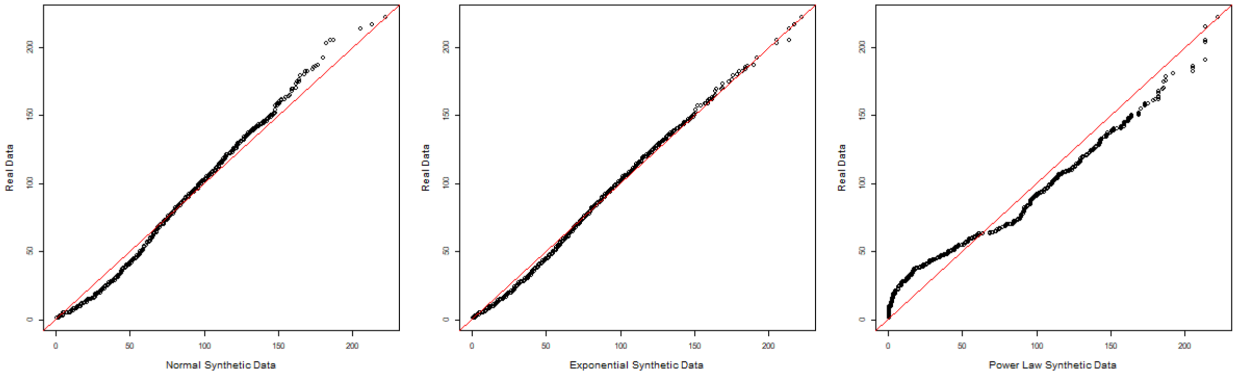

College 140 Cohort 3

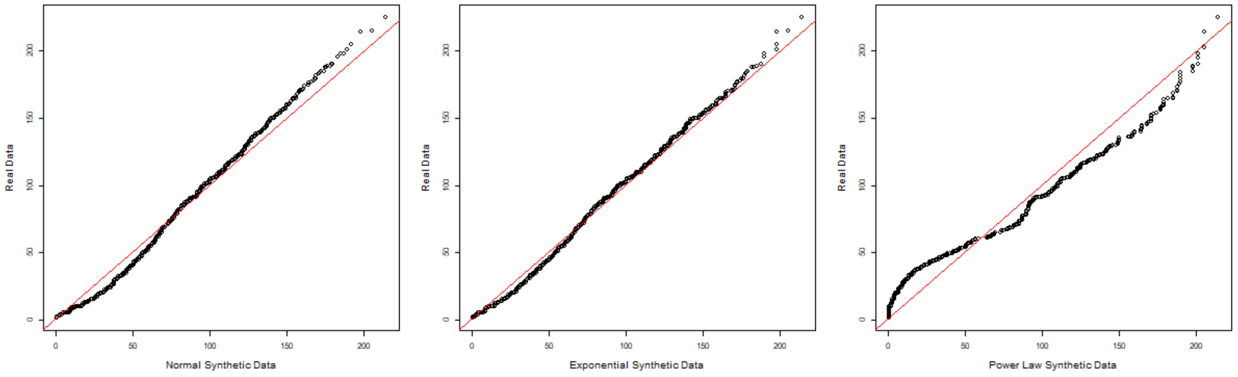

College 140 Cohort 4

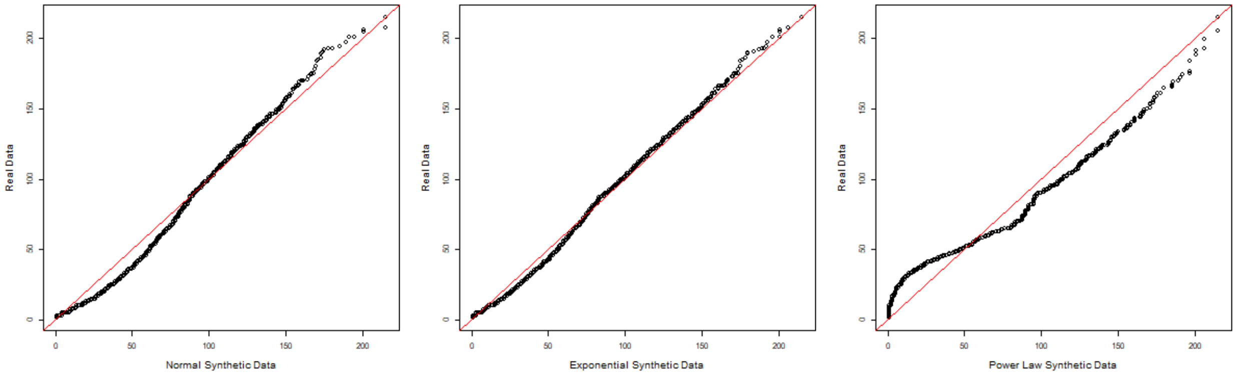

College 140 Cohort 5

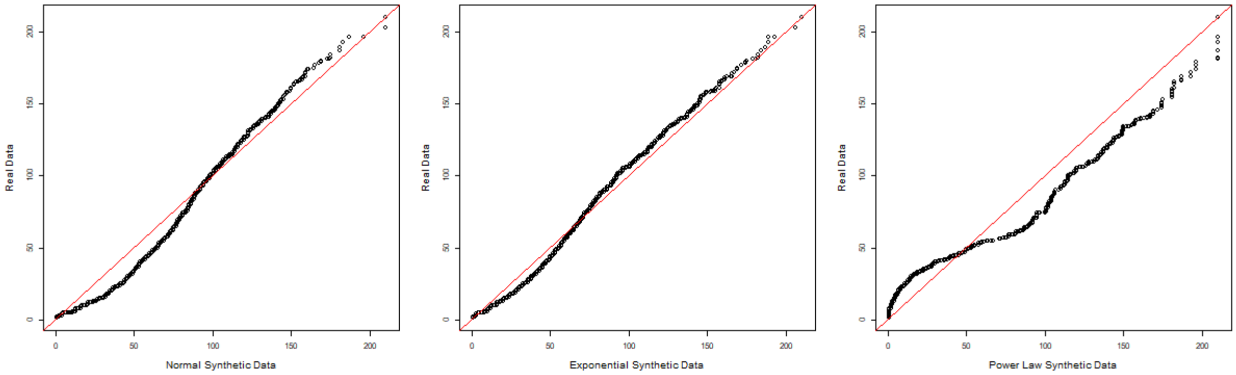

College 150 Cohort 1

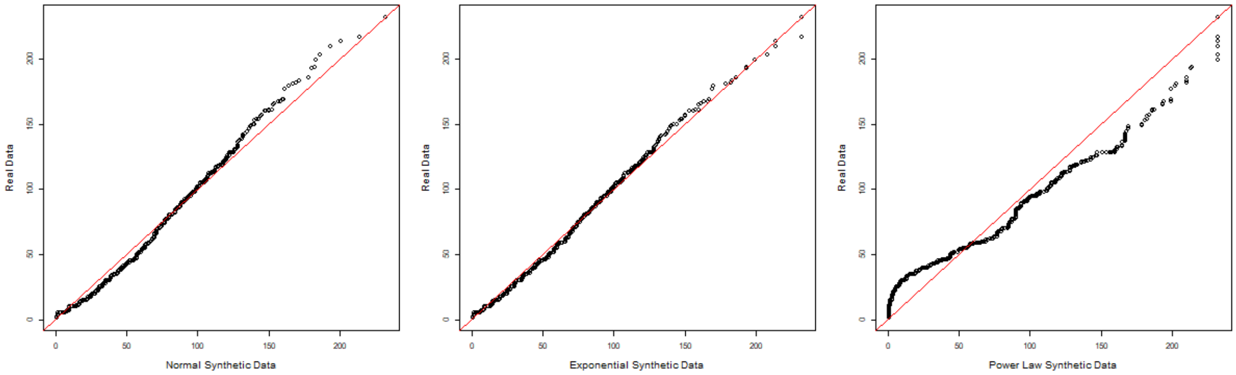

College 150 Cohort 2

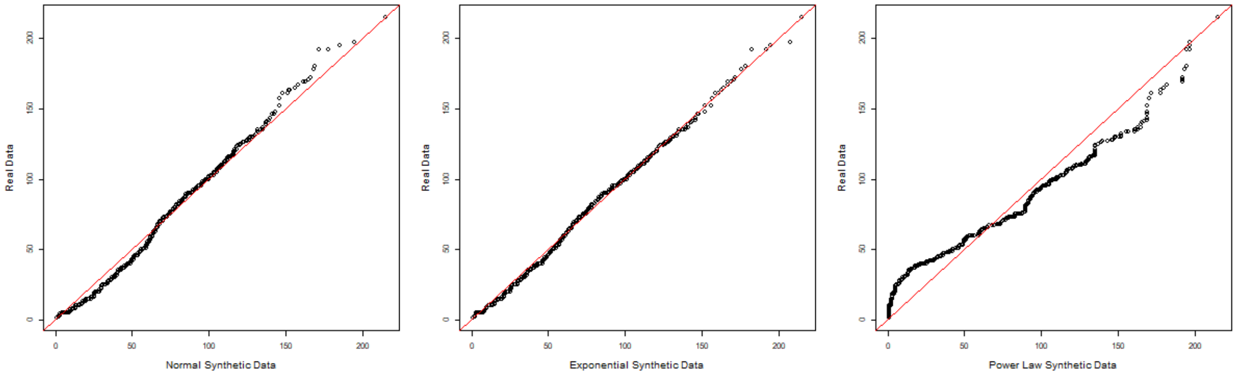

College 150 Cohort 3

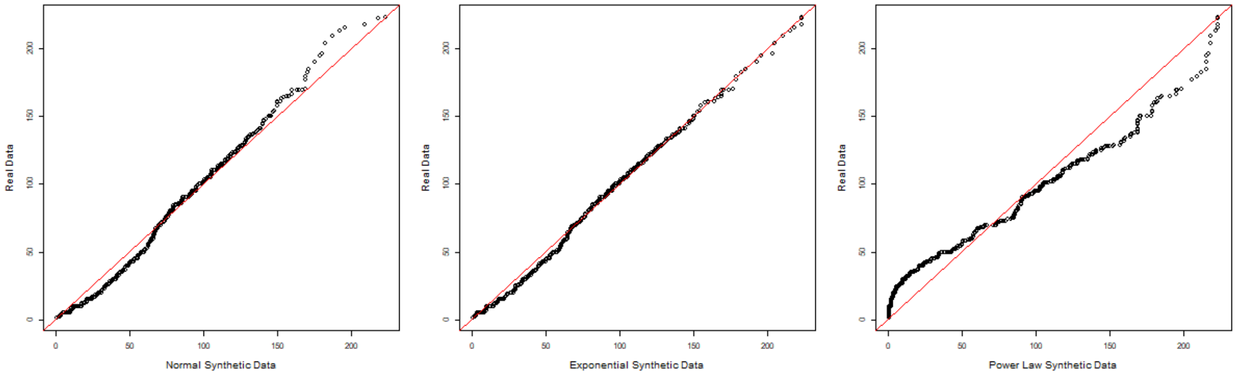

College 150 Cohort 4

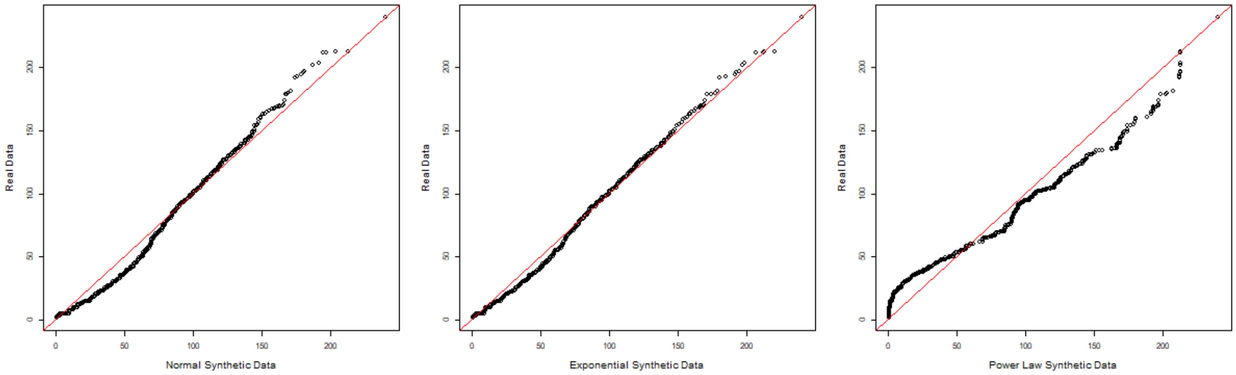

College 150 Cohort 5

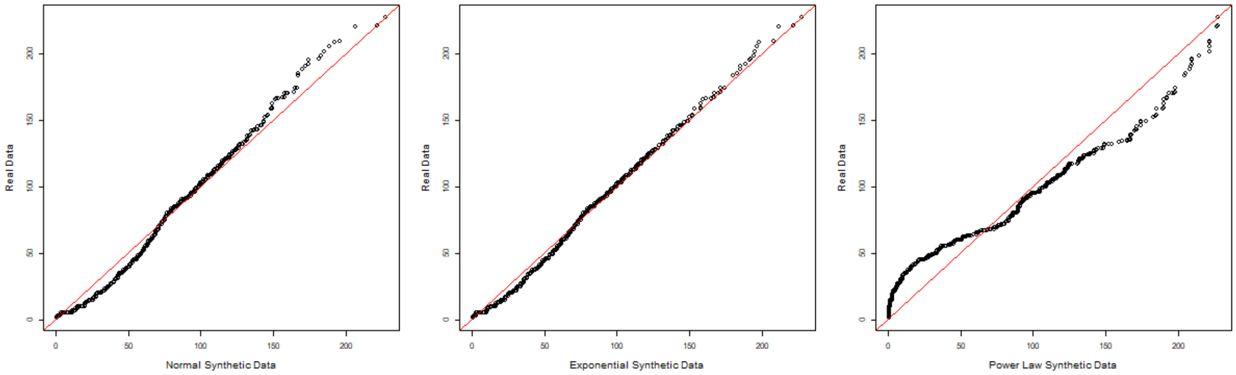

College 160 Cohort 1

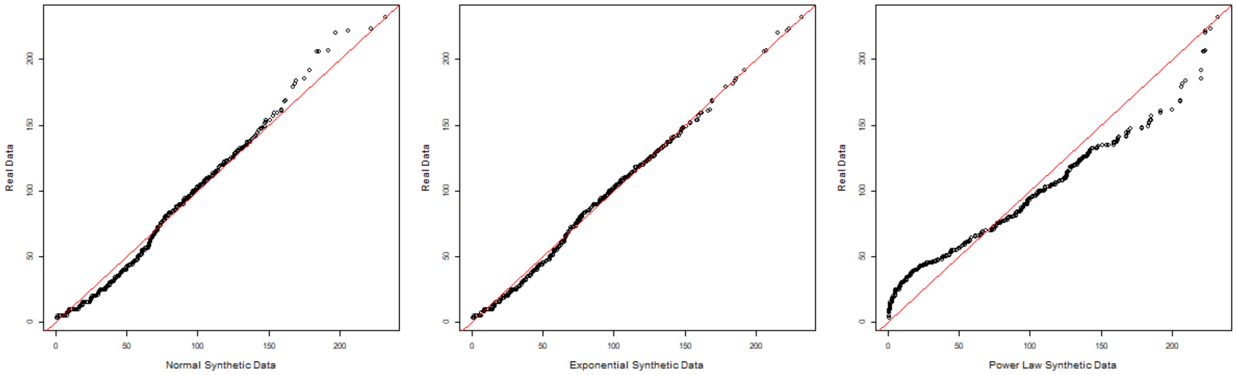

College 160 Cohort 2

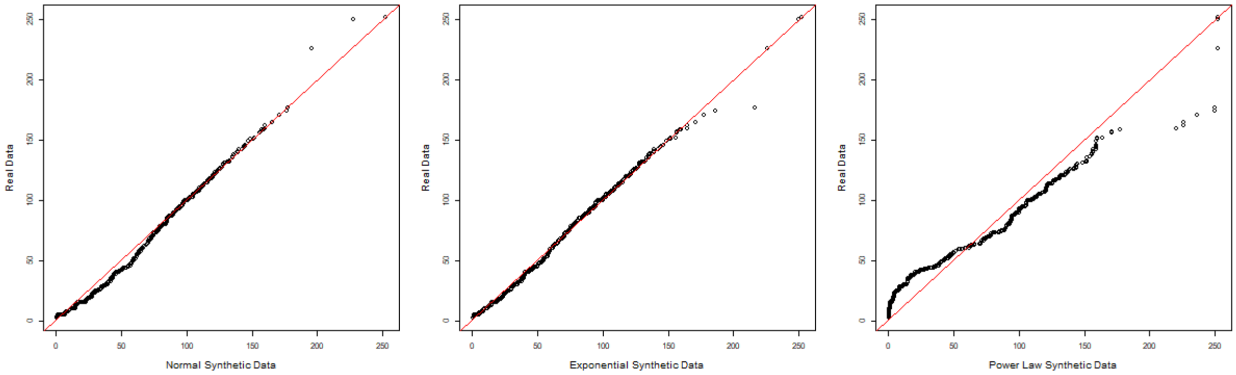

College 160 Cohort 3

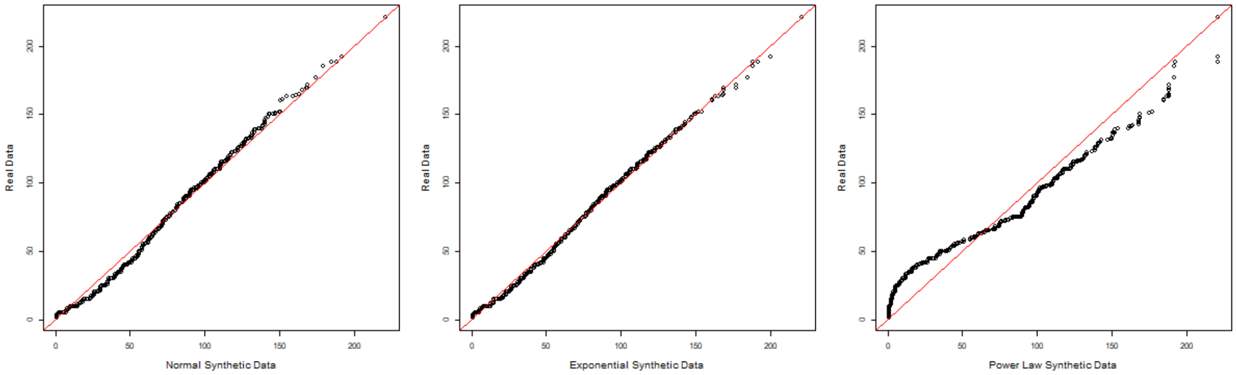

College 160 Cohort 4

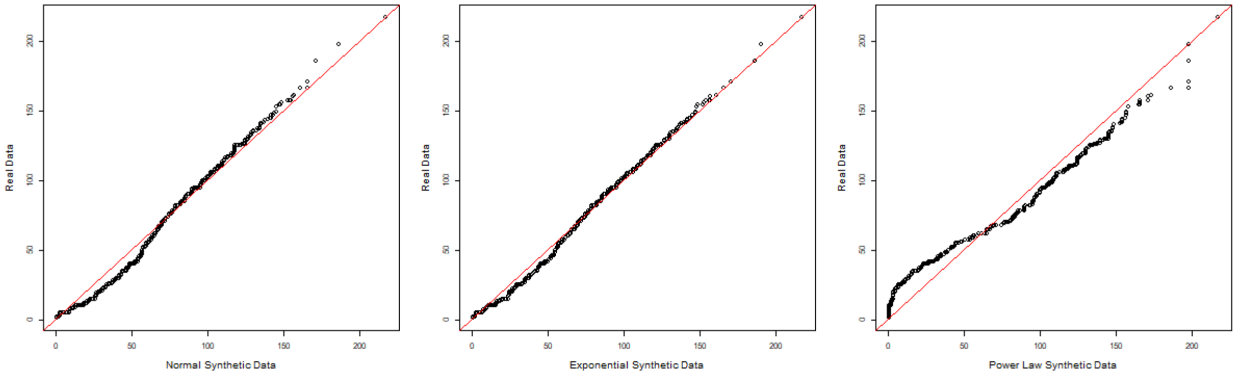

College 160 Cohort 5

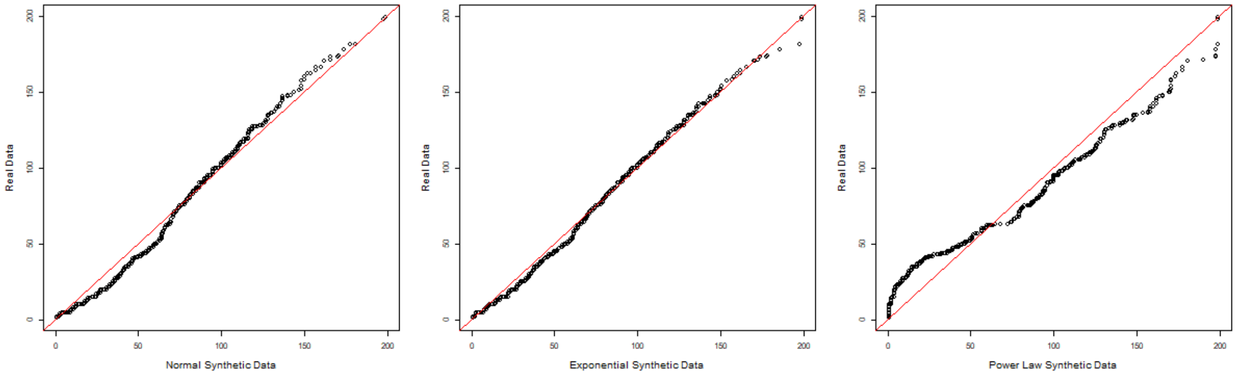

College 171 Cohort 1

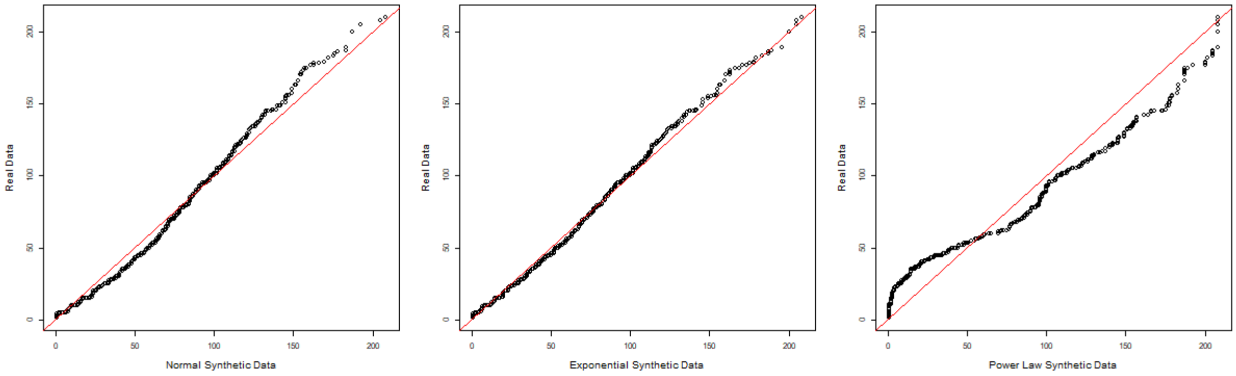

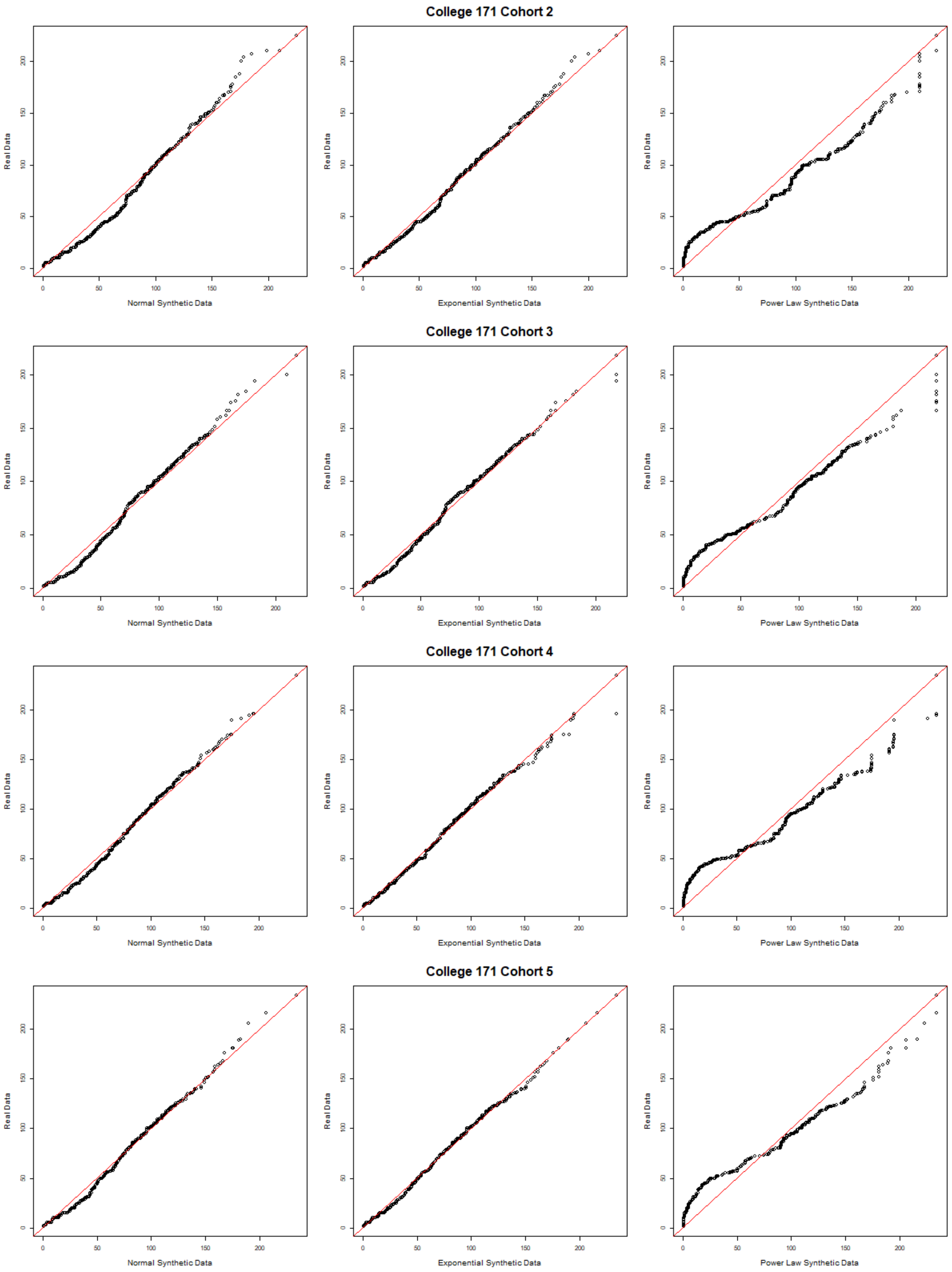
College 171 Cohort 2
College 171 Cohort 3
College 171 Cohort 4
College 171 Cohort 5
Real Data
Normal Synthetic Data
Exponential Synthetic Data
Power Law Synthetic Data

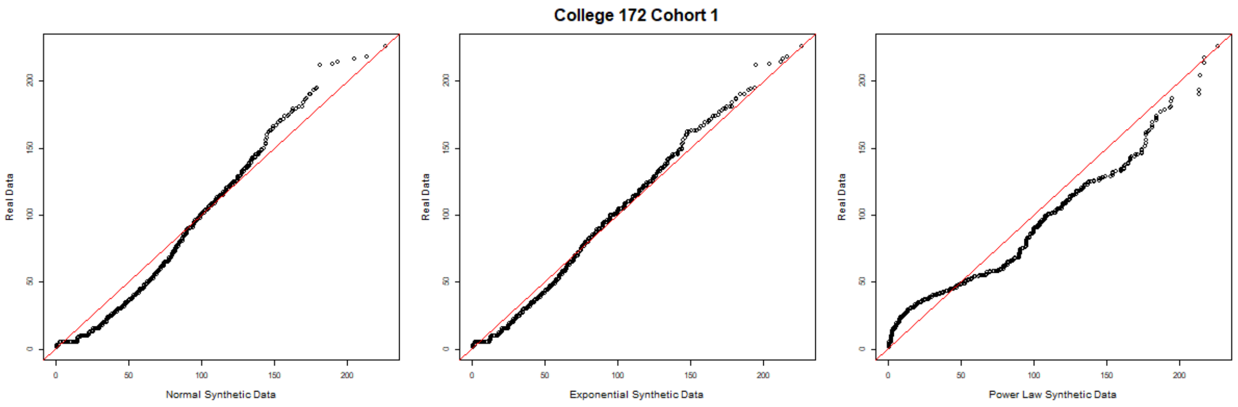
College 172 Cohort 1
Real Data
Normal Synthetic Data
Exponential Synthetic Data
Power Law Synthetic Data

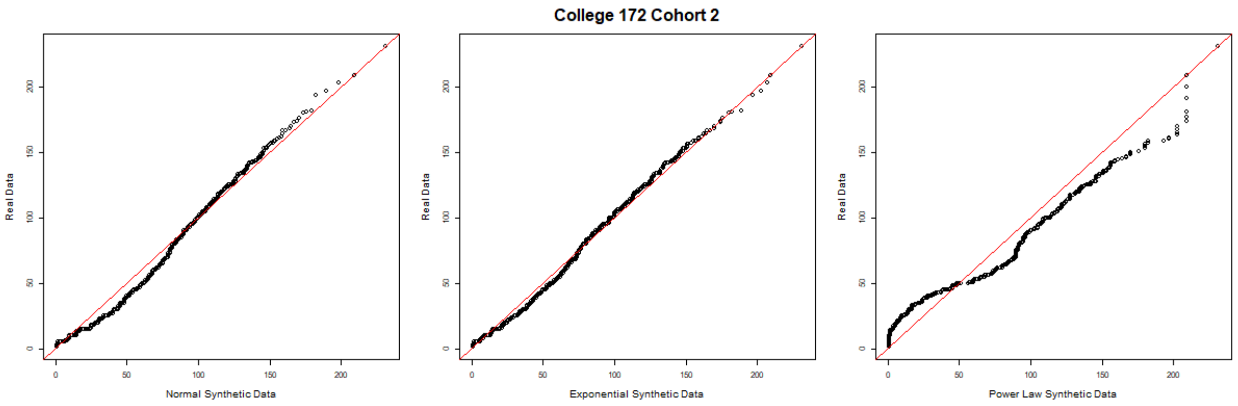
College 172 Cohort 2
Real Data
Normal Synthetic Data
Exponential Synthetic Data
Power Law Synthetic Data

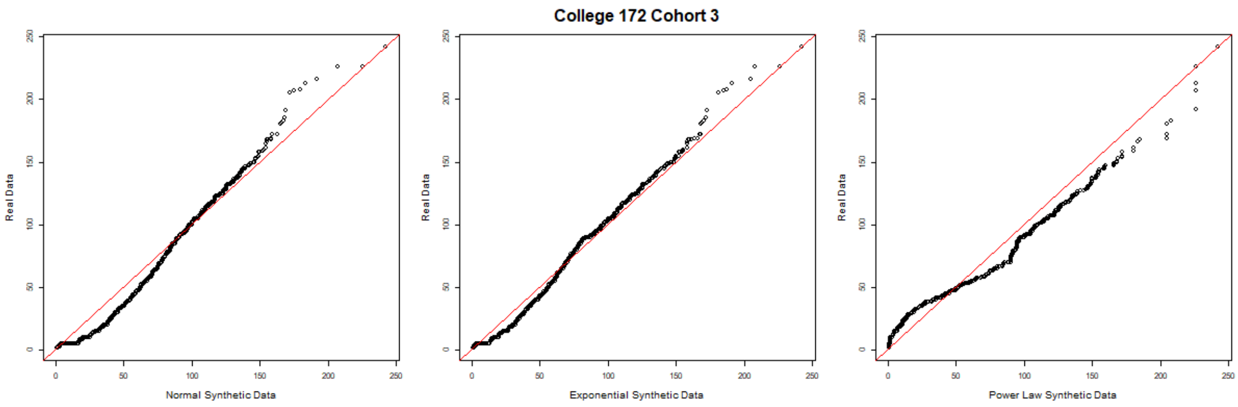
College 172 Cohort 3
Real Data
Normal Synthetic Data
Exponential Synthetic Data
Power Law Synthetic Data

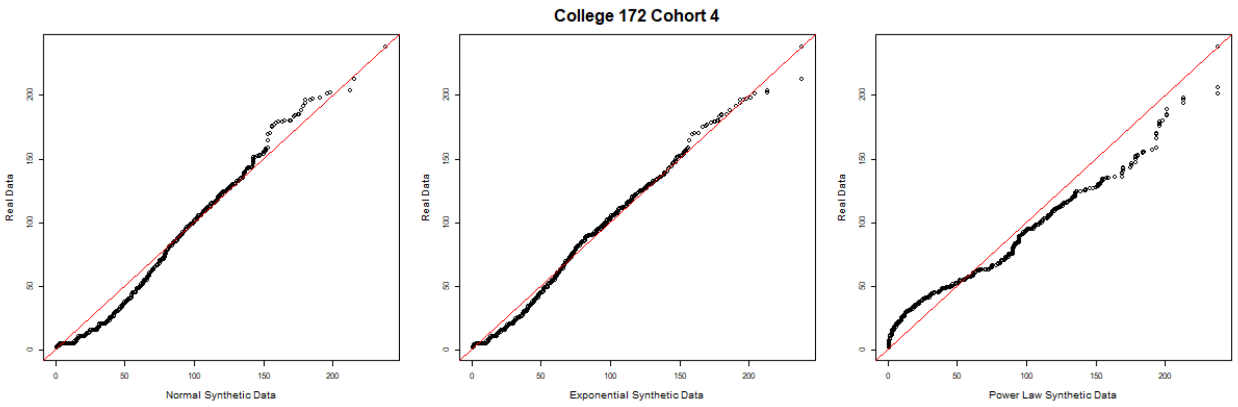
College 172 Cohort 4
Real Data
Normal Synthetic Data
Exponential Synthetic Data
Power Law Synthetic Data

College 172 Cohort 5

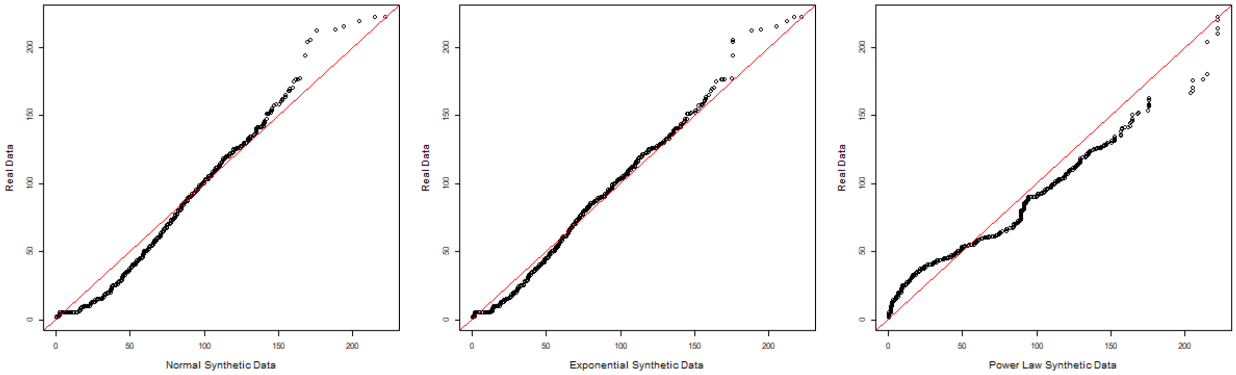

College 180 Cohort 1

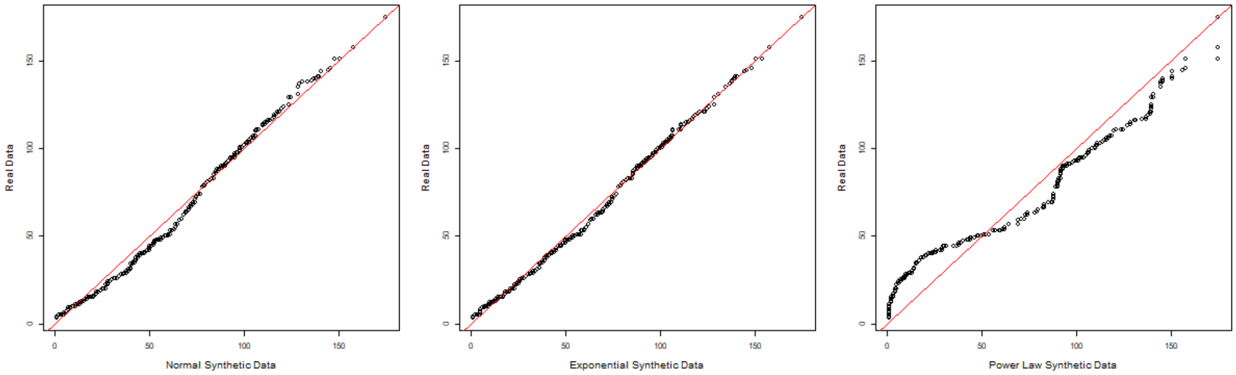

College 180 Cohort 2

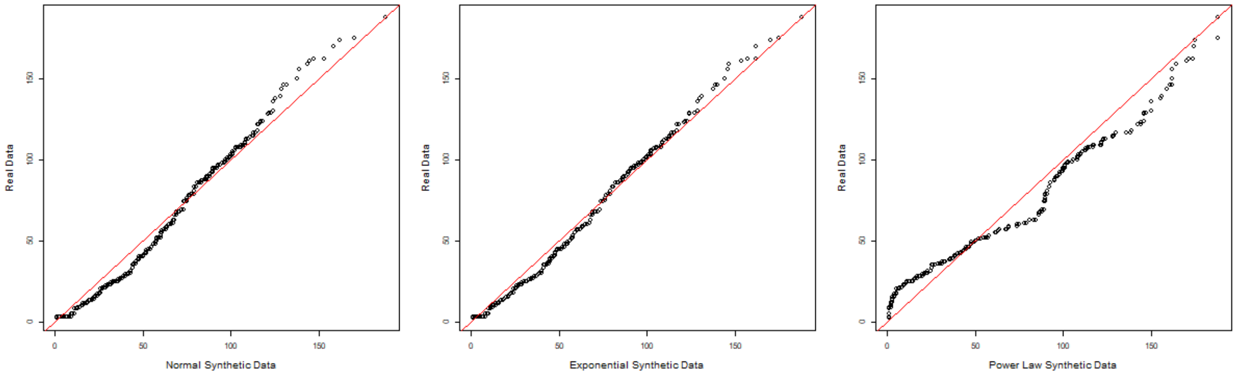

College 180 Cohort 3

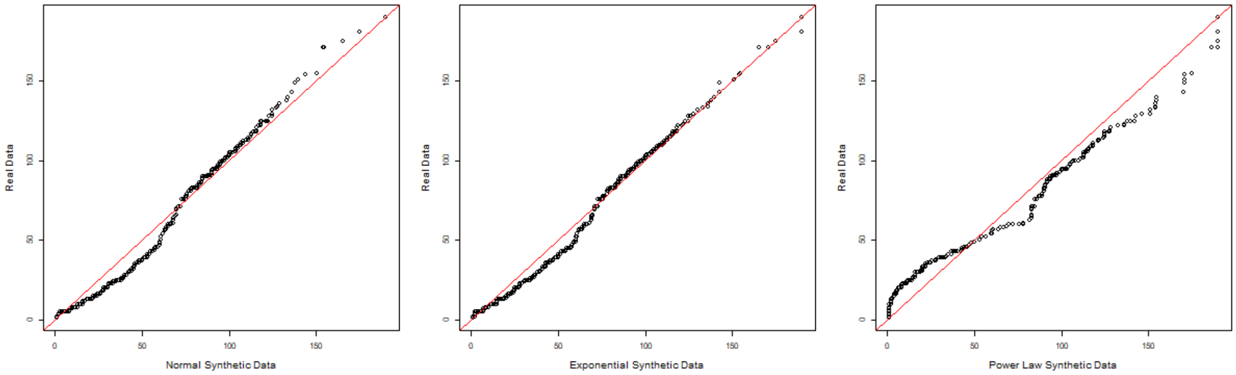

College 180 Cohort 4

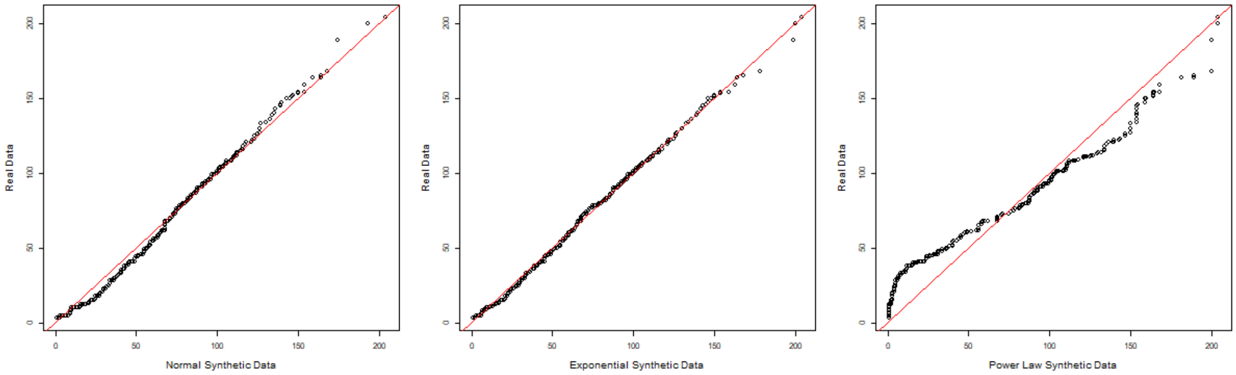

College 180 Cohort 5

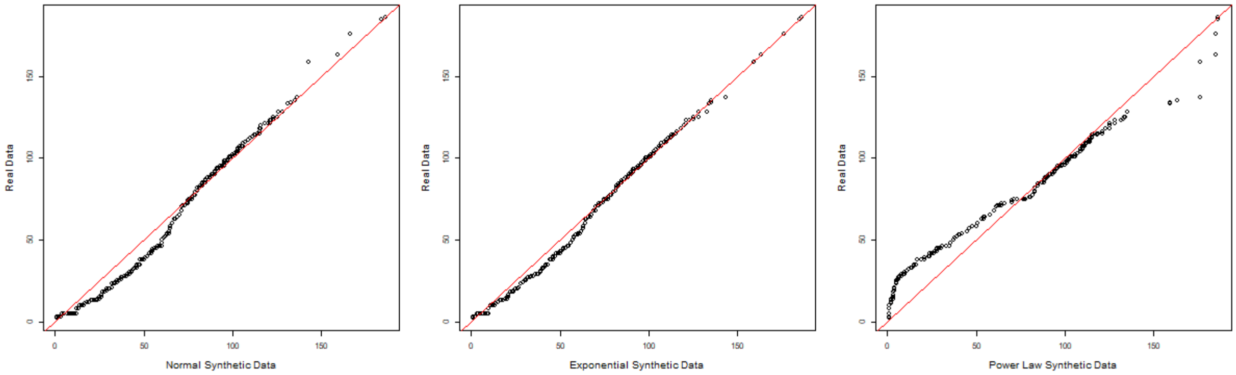

College 190 Cohort 1

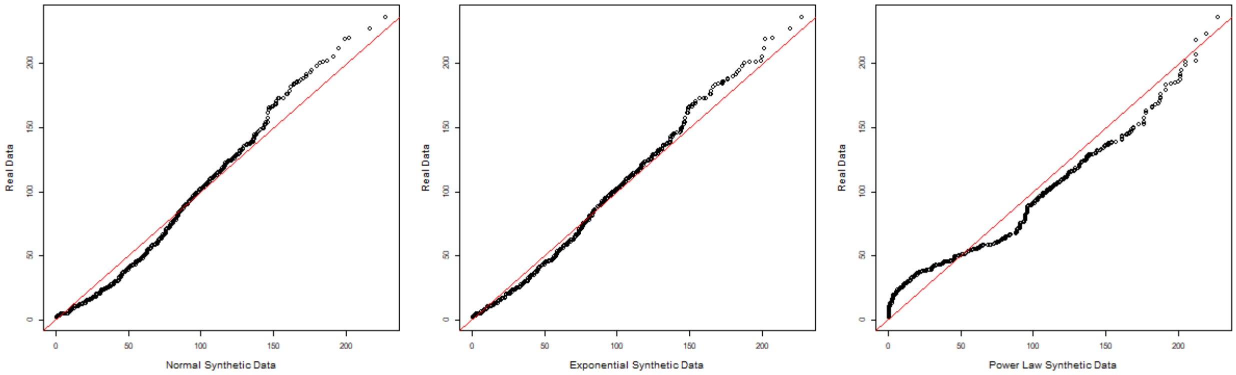

College 190 Cohort 2

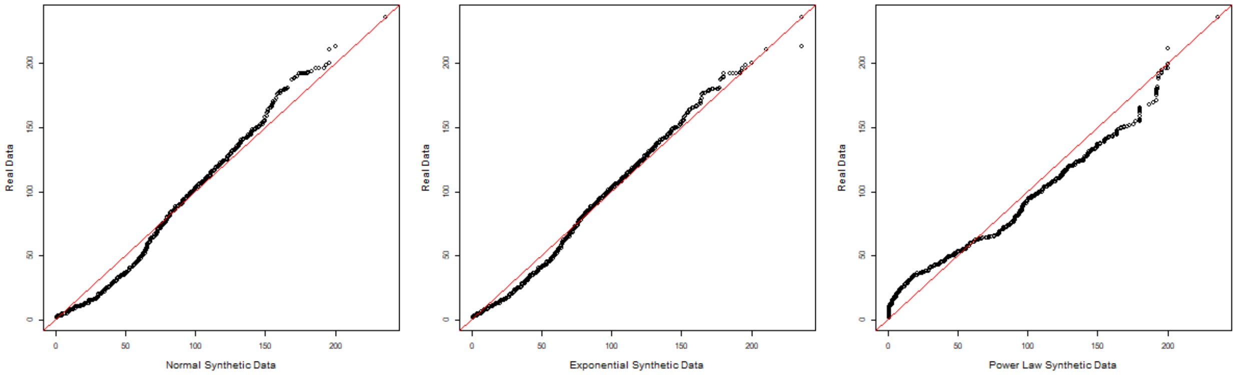

College 190 Cohort 3

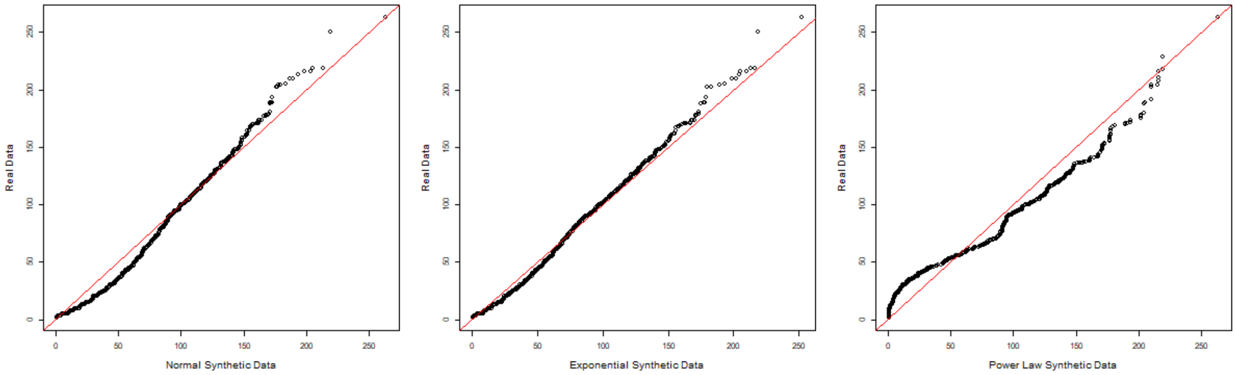

College 190 Cohort 4

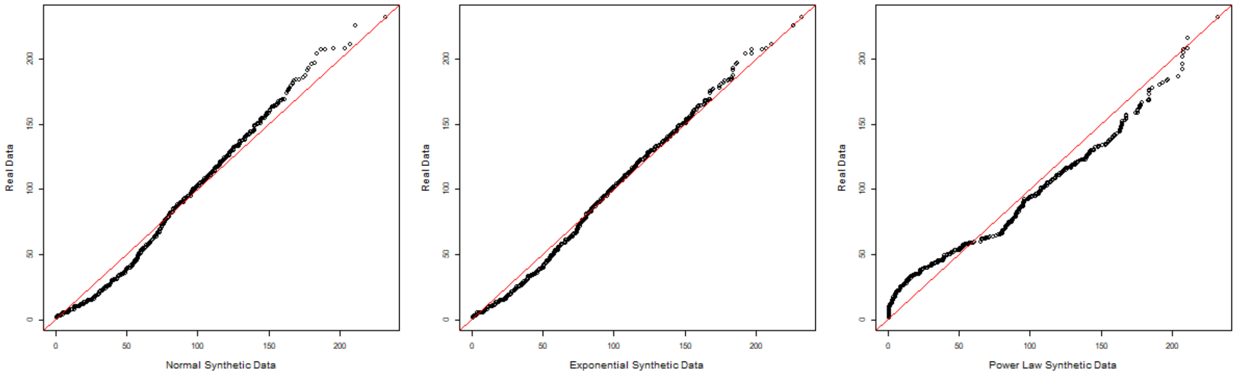

College 190 Cohort 5

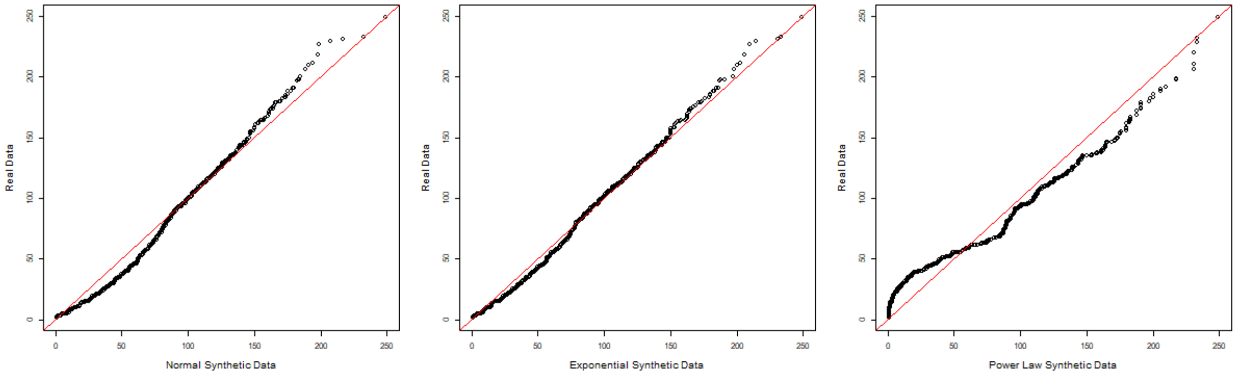

College 200 Cohort 1

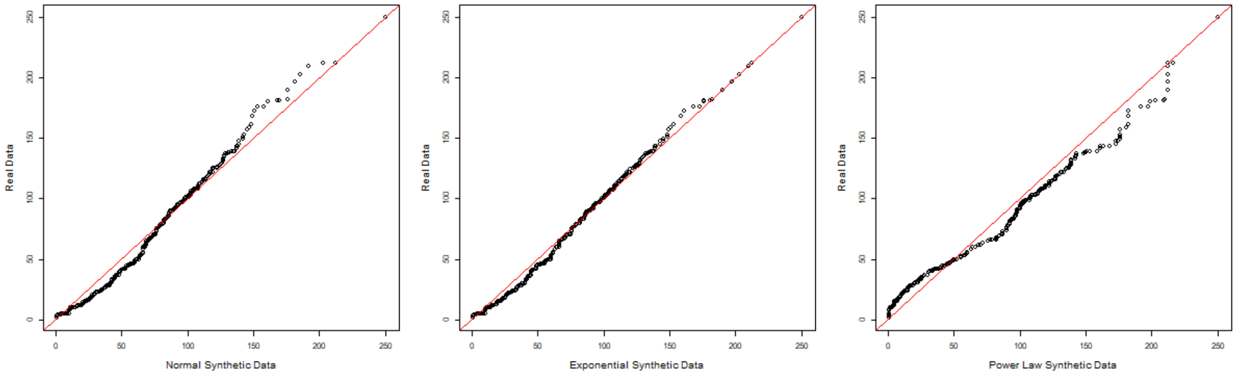

College 200 Cohort 2

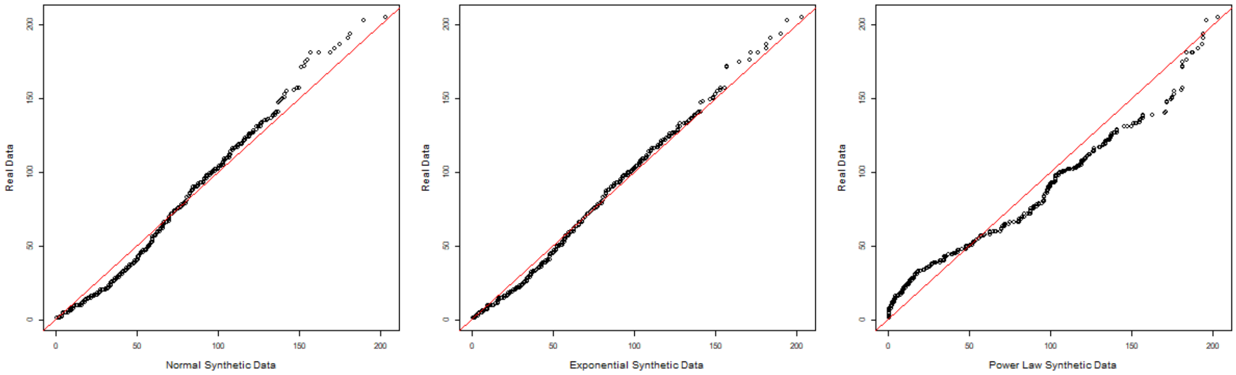

College 200 Cohort 3

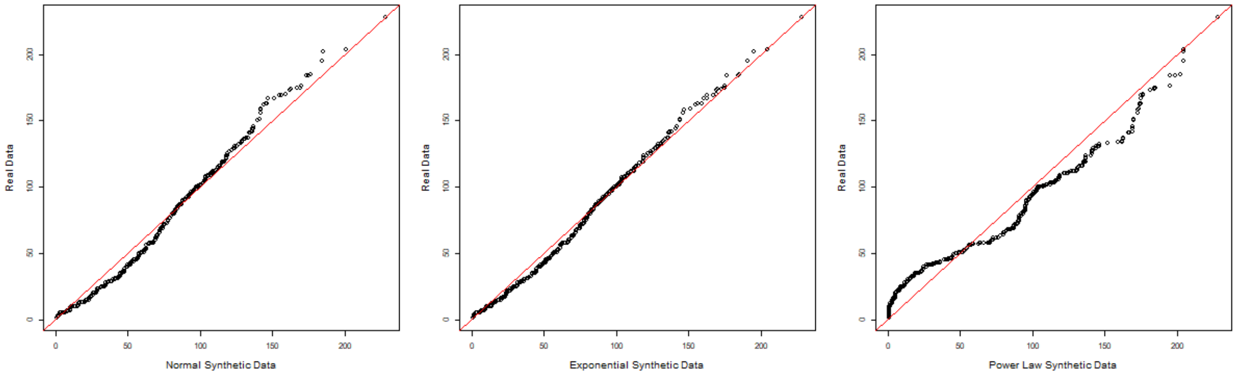

College 200 Cohort 4

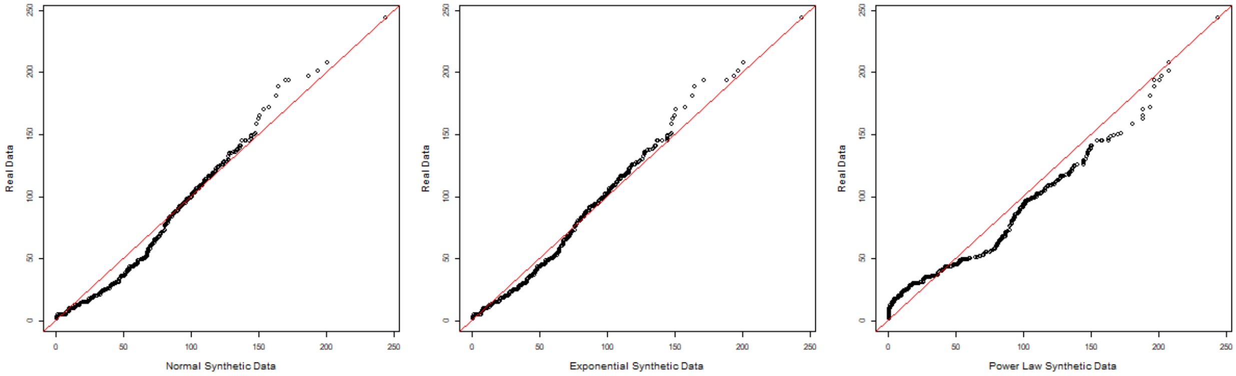

College 200 Cohort 5

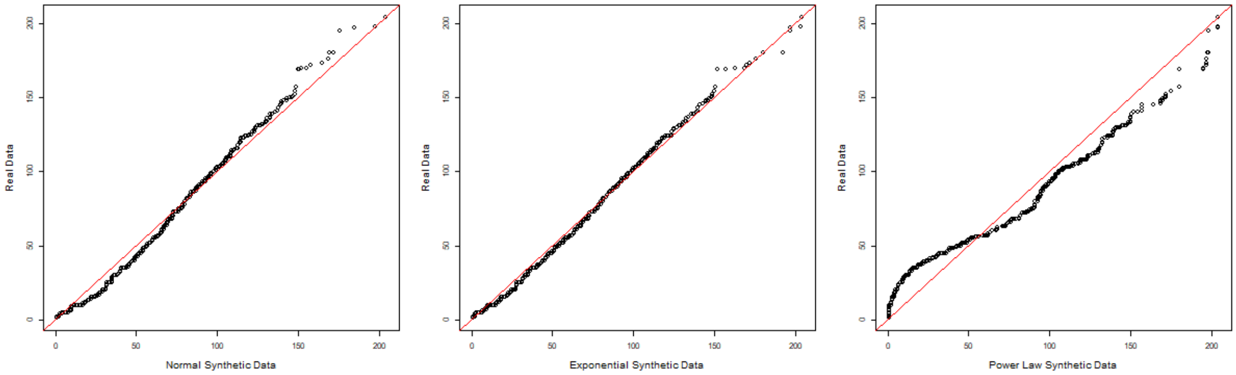

College 210 Cohort 1

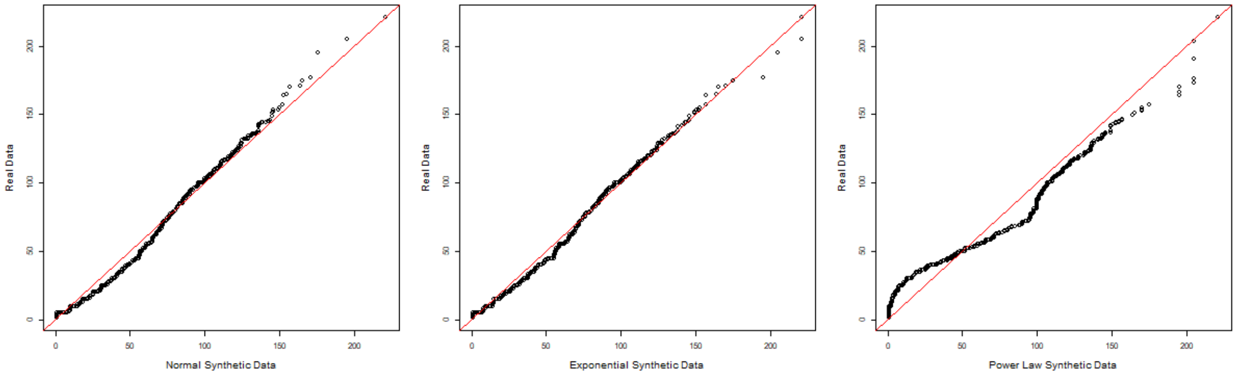

College 210 Cohort 2

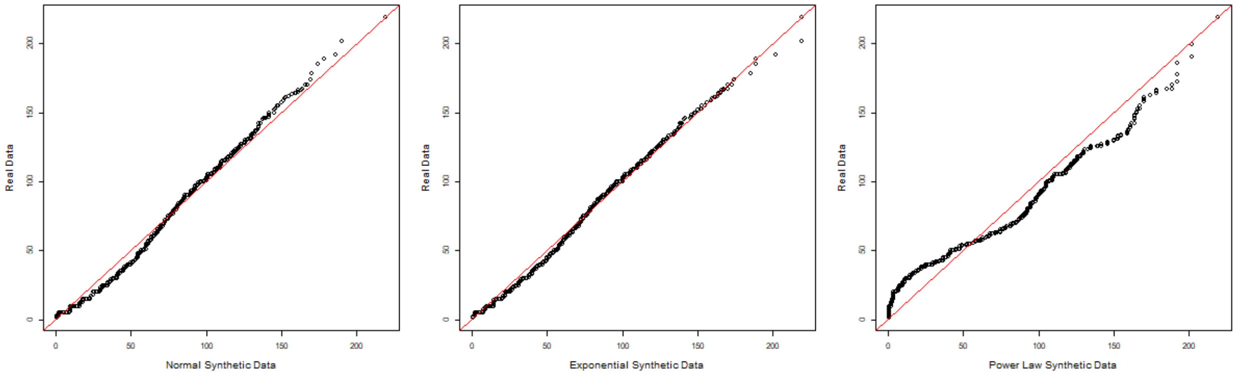

College 210 Cohort 3

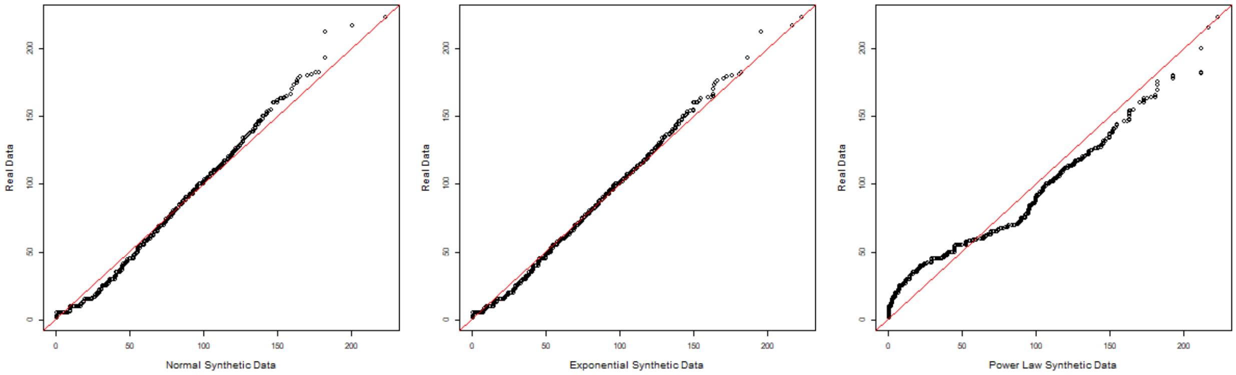

College 210 Cohort 4

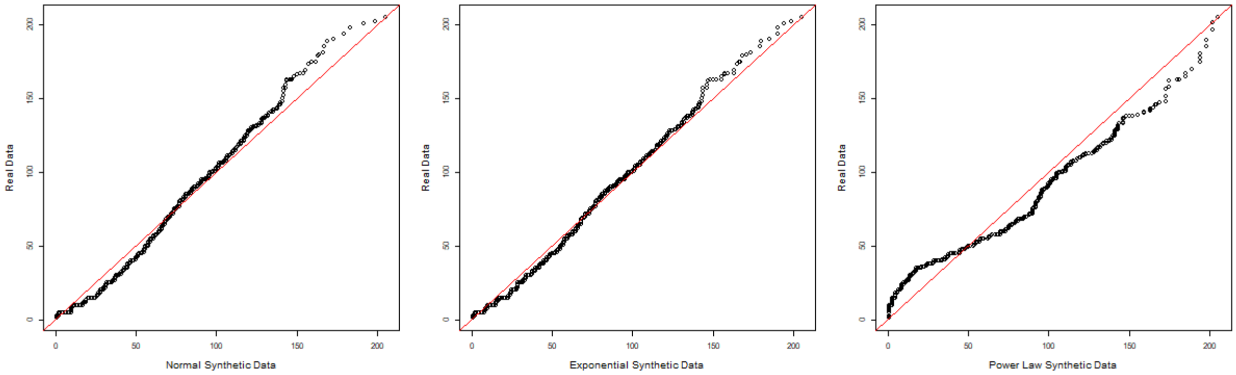

College 210 Cohort 5

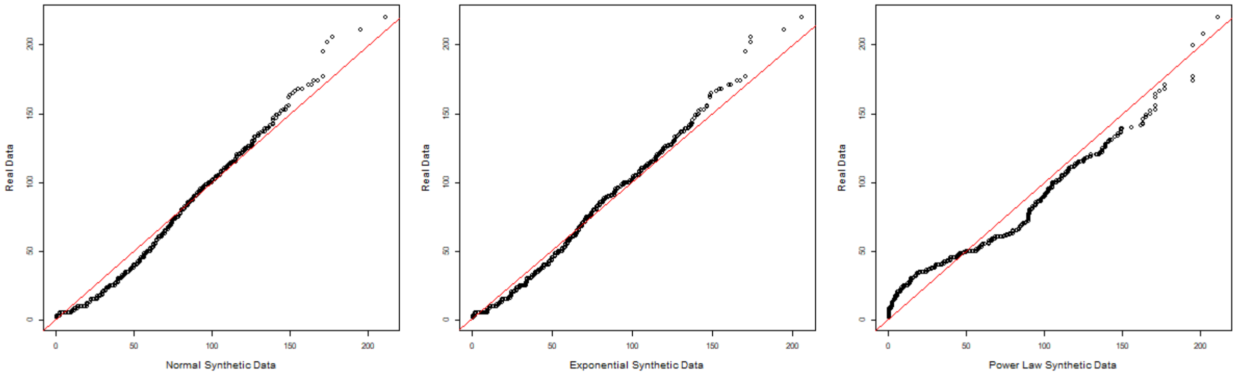

College 220 Cohort 1

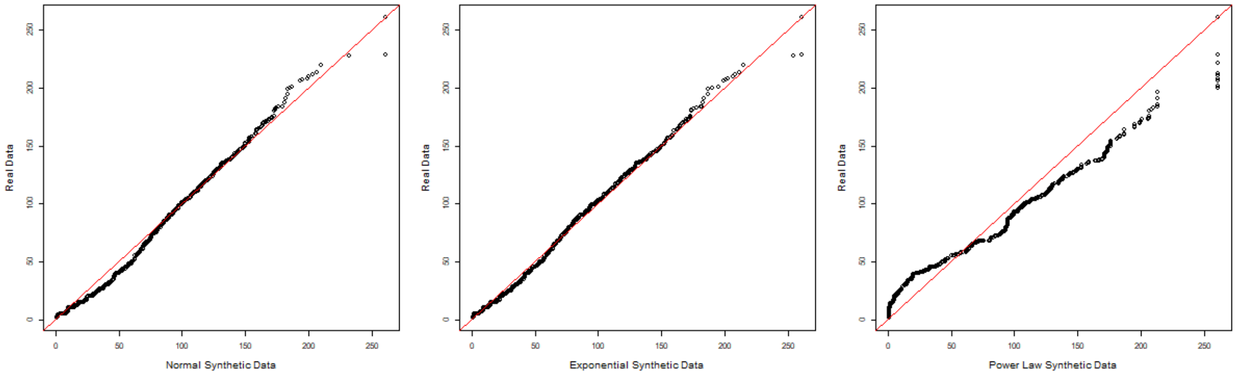

College 220 Cohort 2

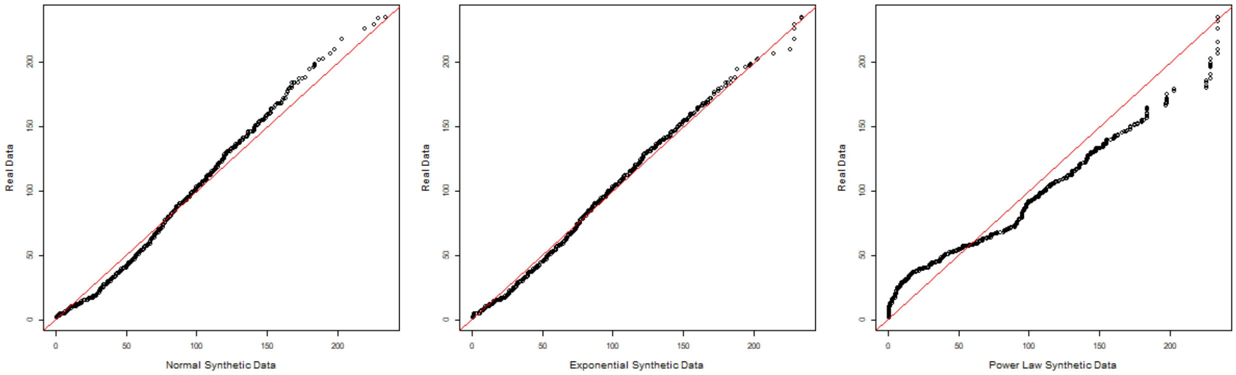

College 220 Cohort 3

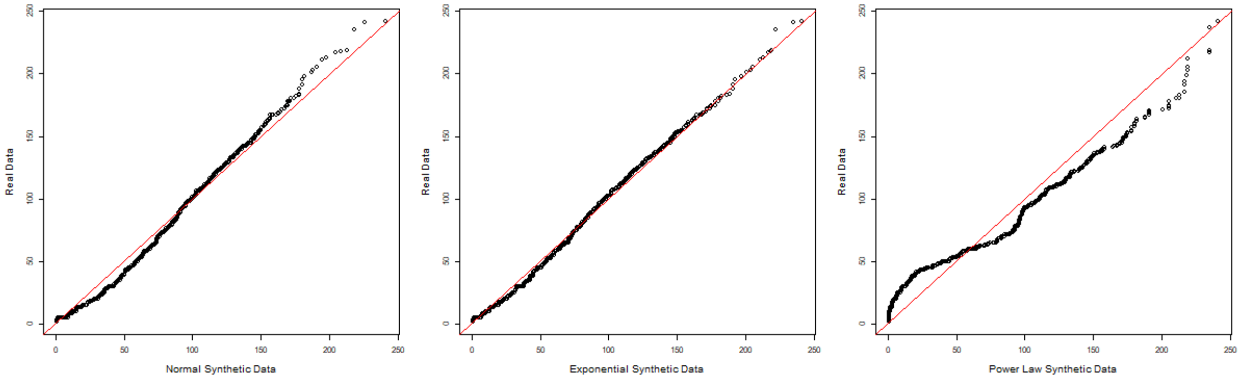

College 220 Cohort 4

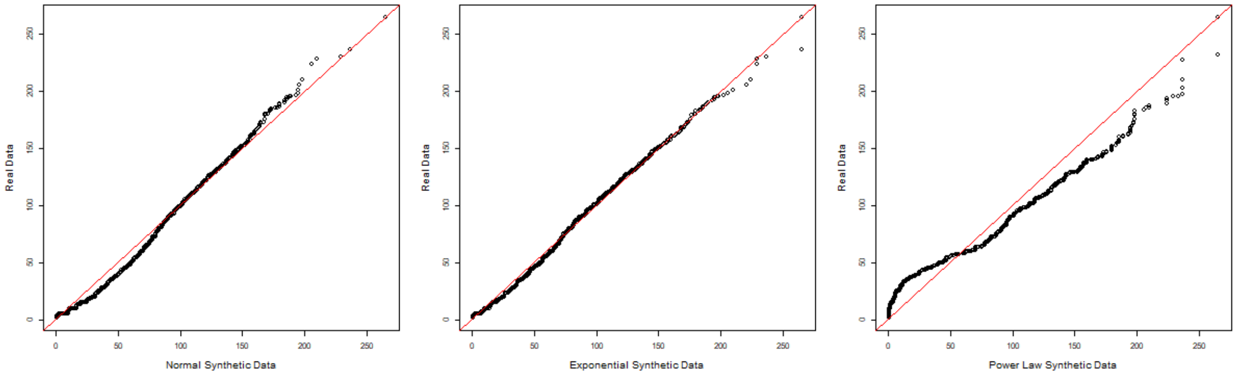

College 220 Cohort 5

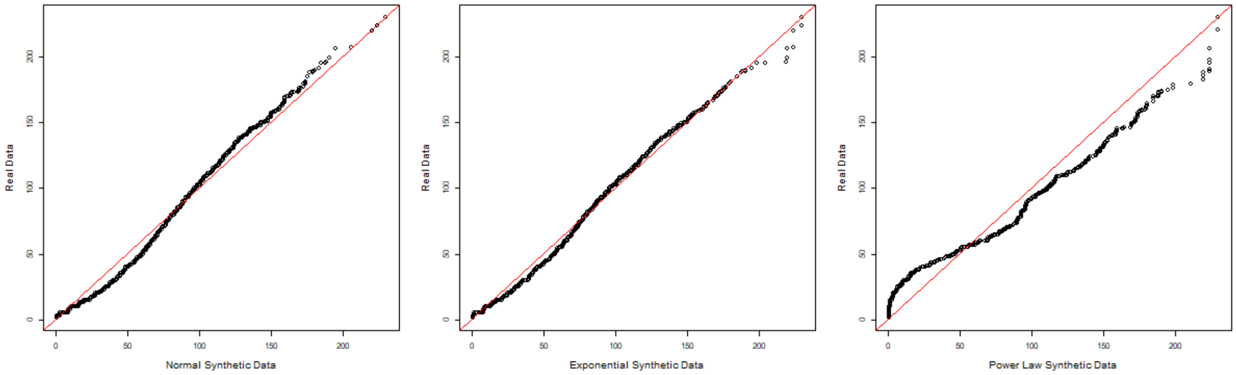

College 230 Cohort 1

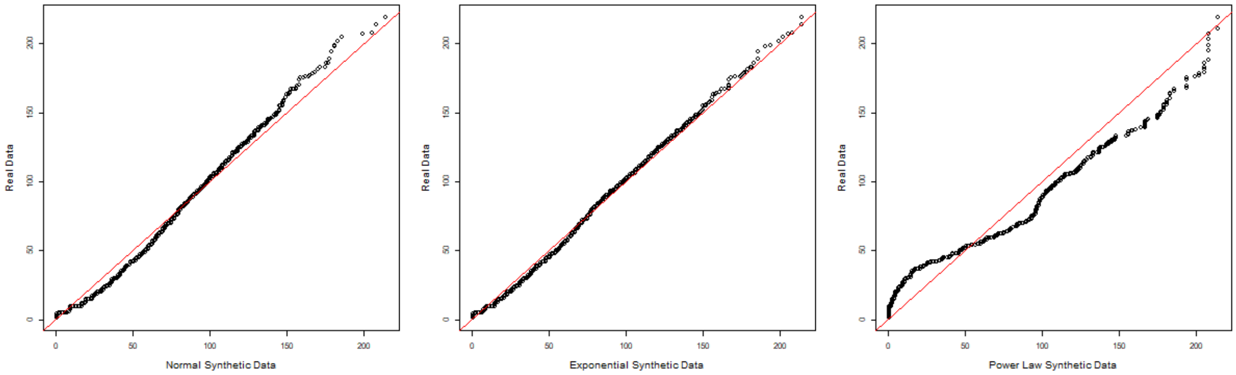

College 230 Cohort 2

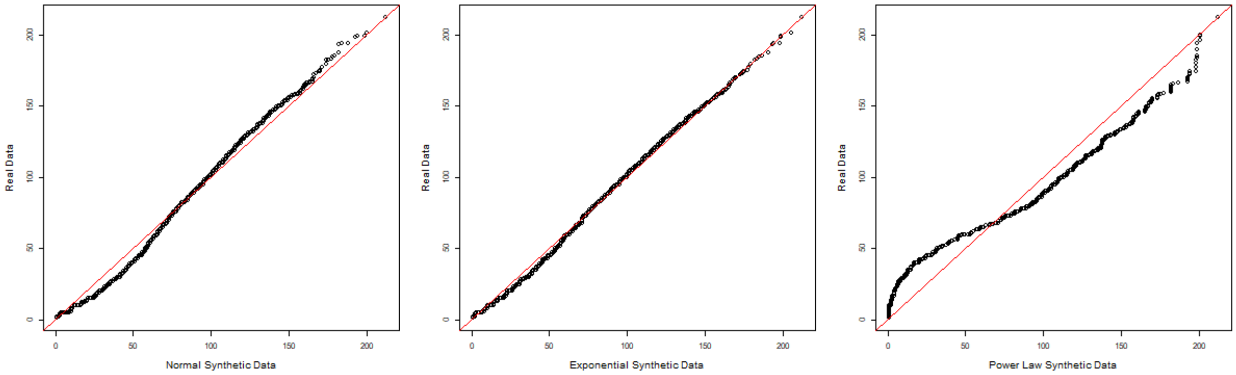

College 230 Cohort 3

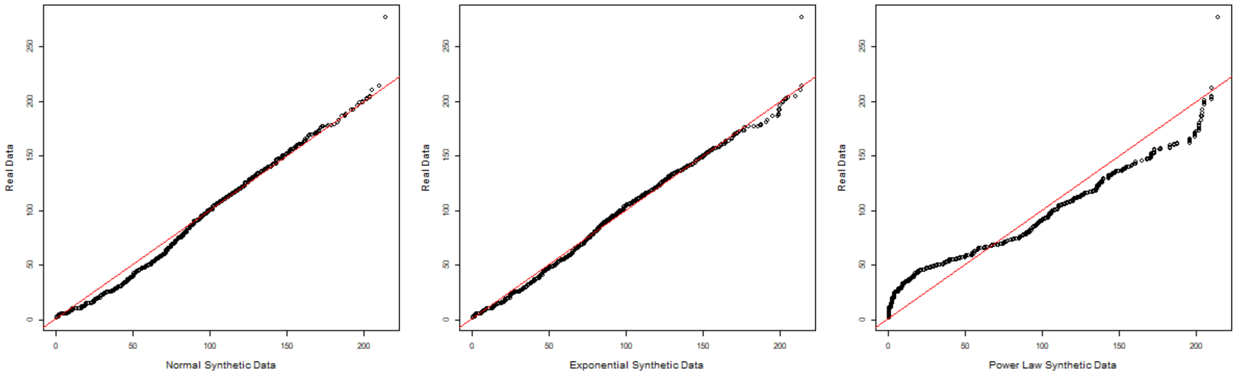

College 230 Cohort 4

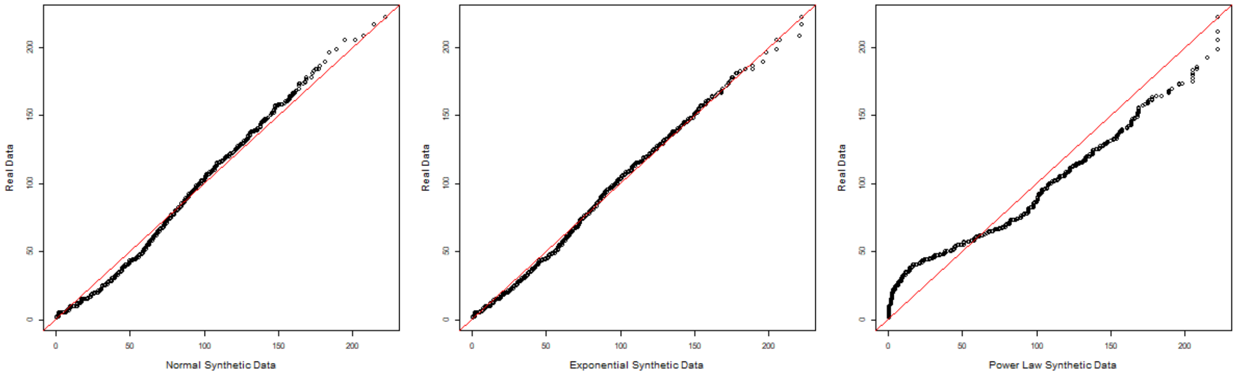

College 230 Cohort 5

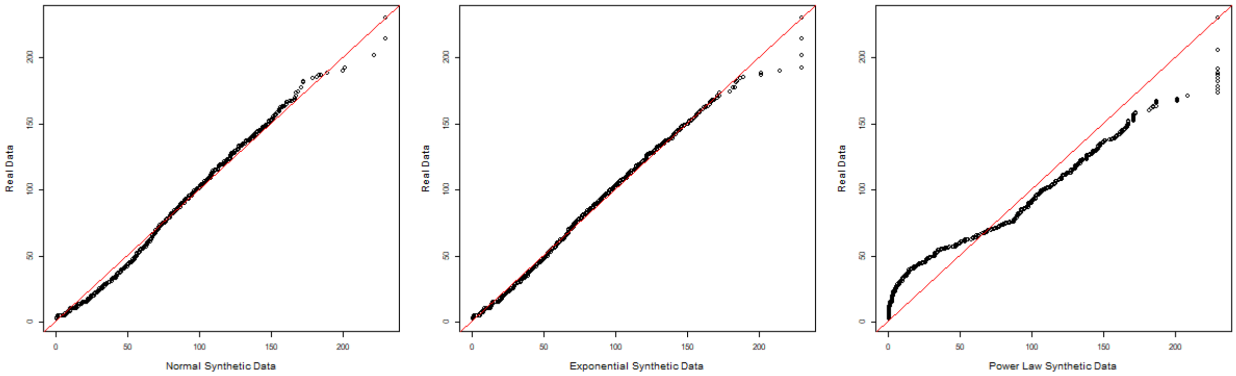

College 240 Cohort 1

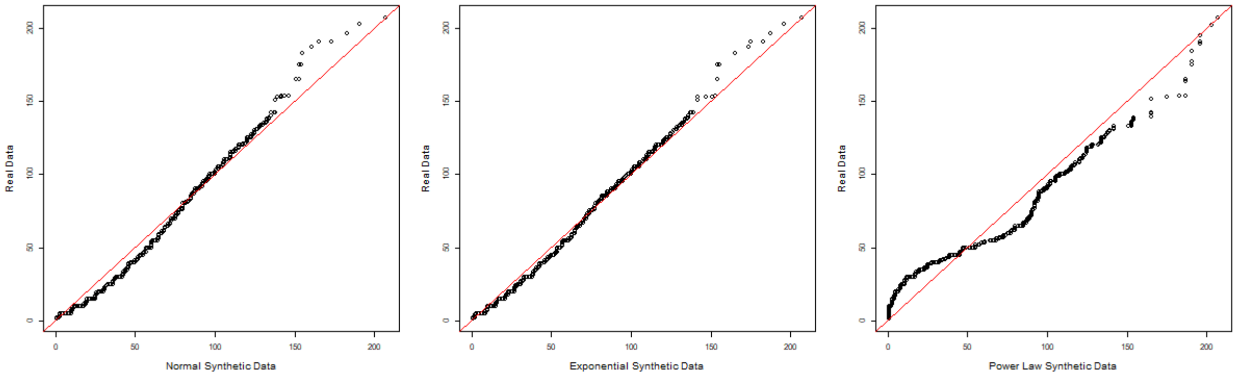

College 240 Cohort 2

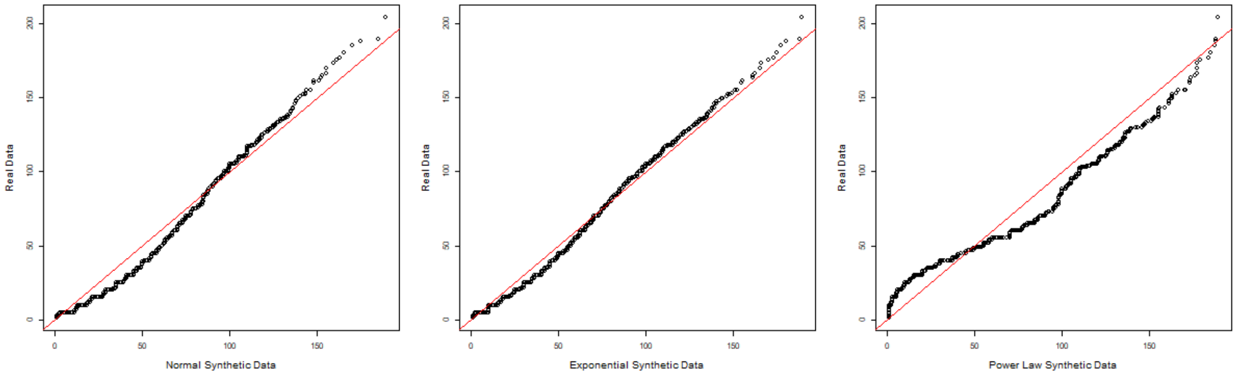

College 240 Cohort 3

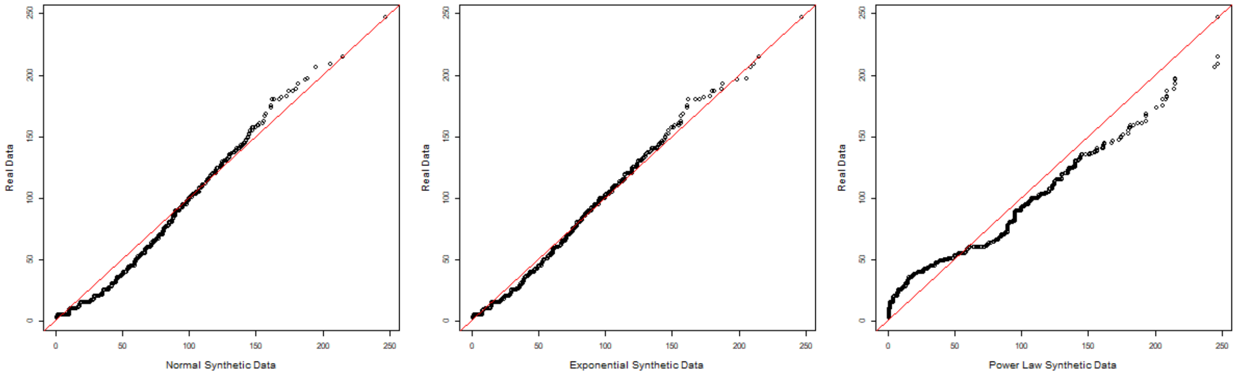

College 240 Cohort 4

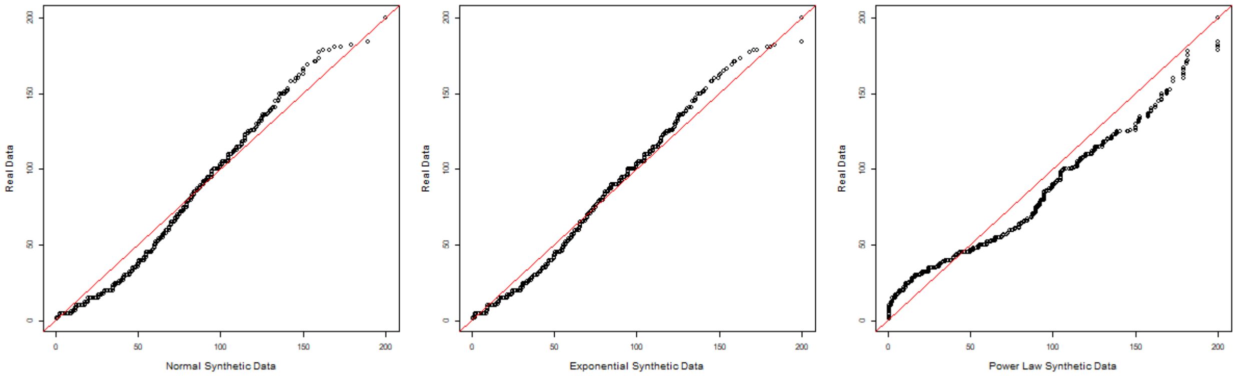

College 240 Cohort 5

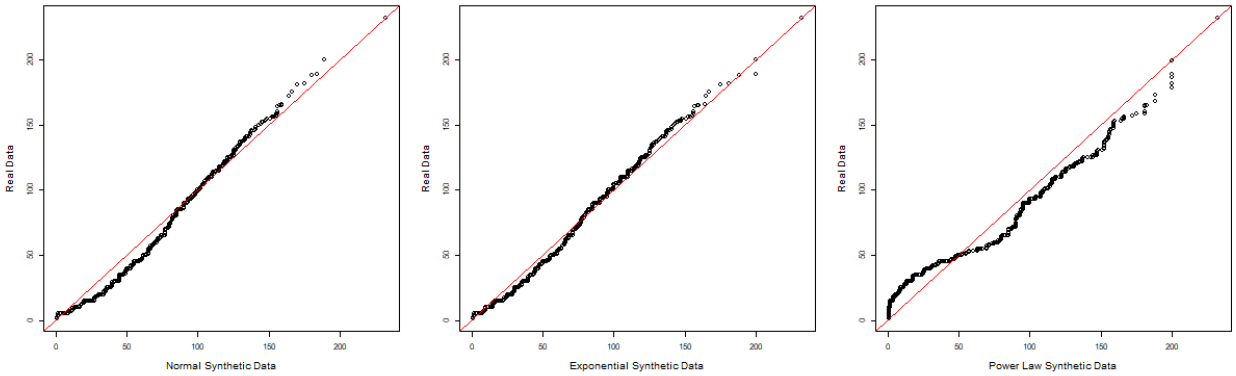

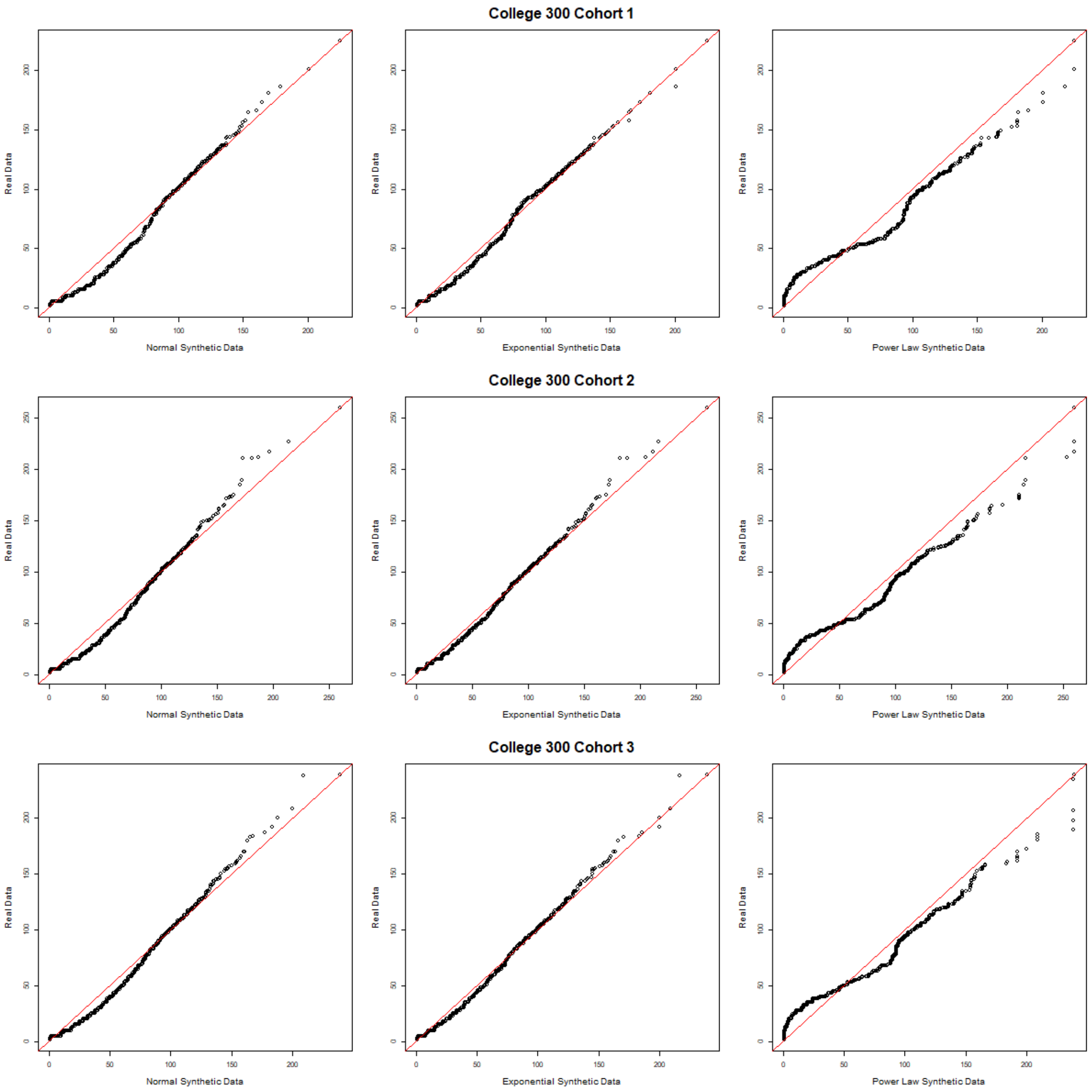
College 300 Cohort 1
College 300 Cohort 2
College 300 Cohort 3
Real Data
Normal Synthetic Data
Exponential Synthetic Data
Power Law Synthetic Data

College 300 Cohort 4

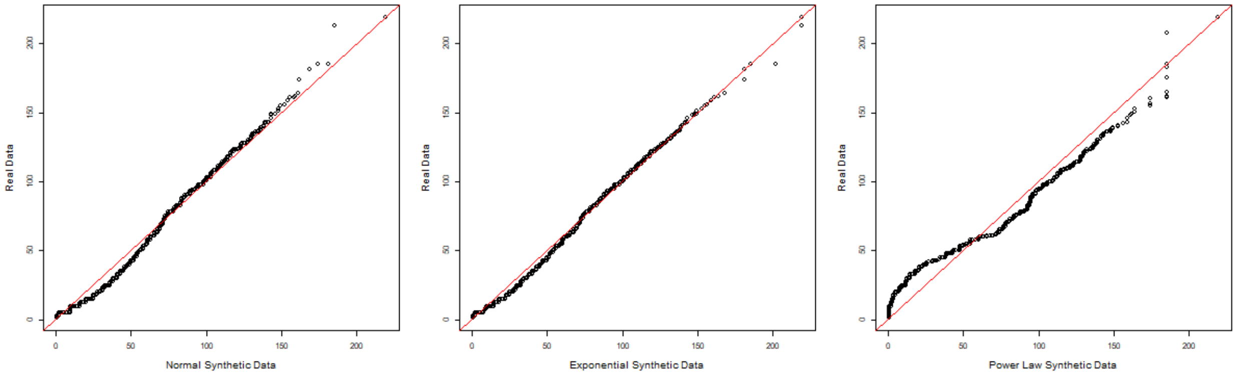

College 300 Cohort 5

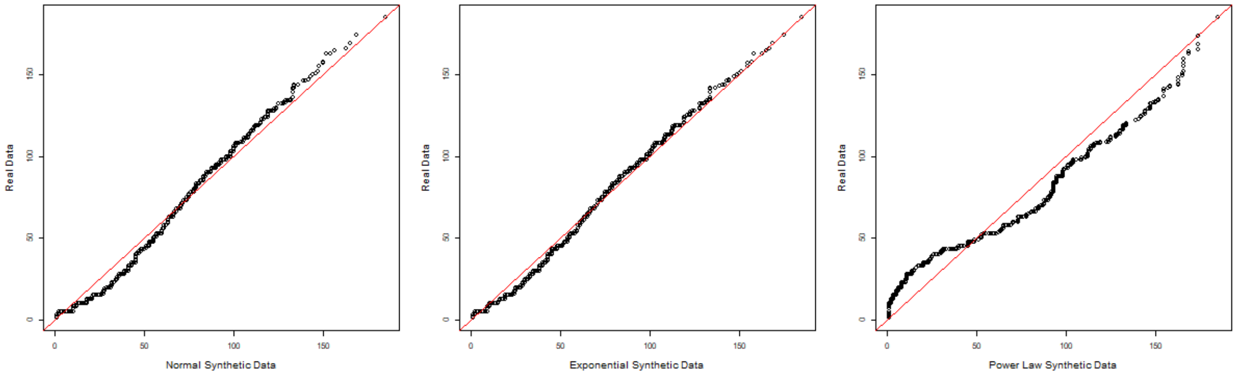

Fig. S3. Summary statistics by cohort. A cohort is the set of all students who started at a given college in a given academic year.

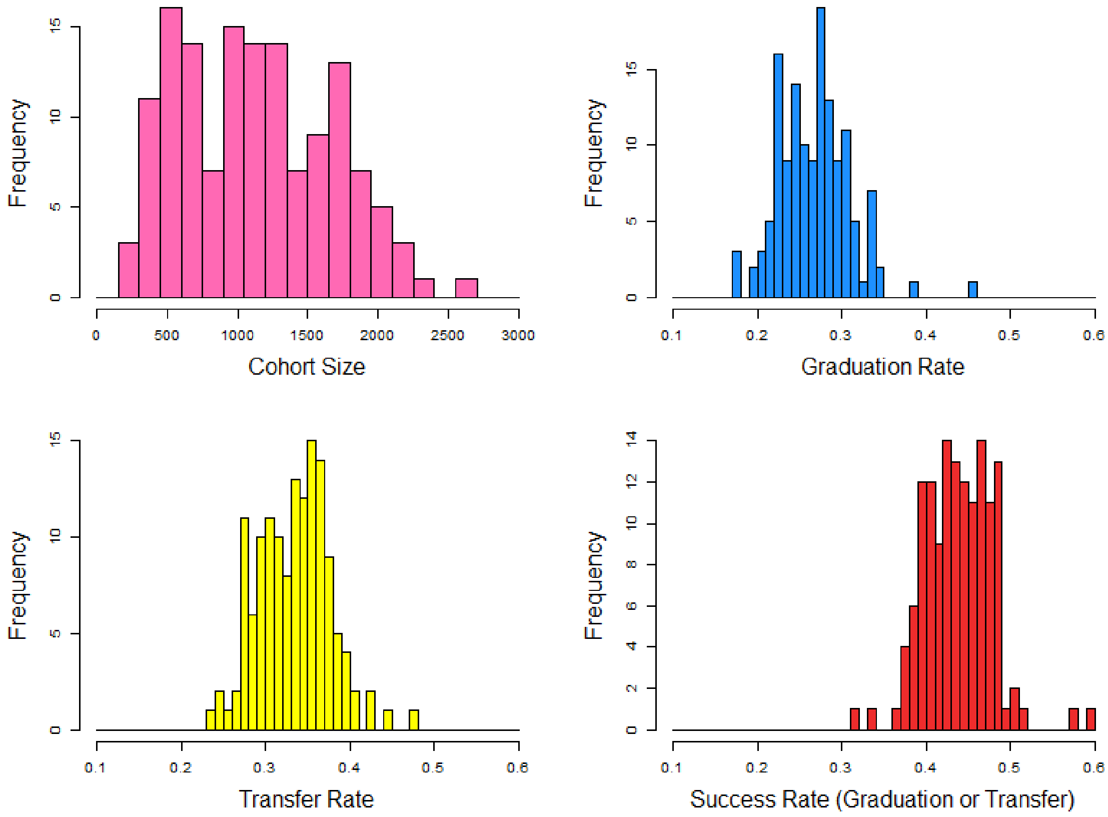

Fig. S4. Summary statistics by college.

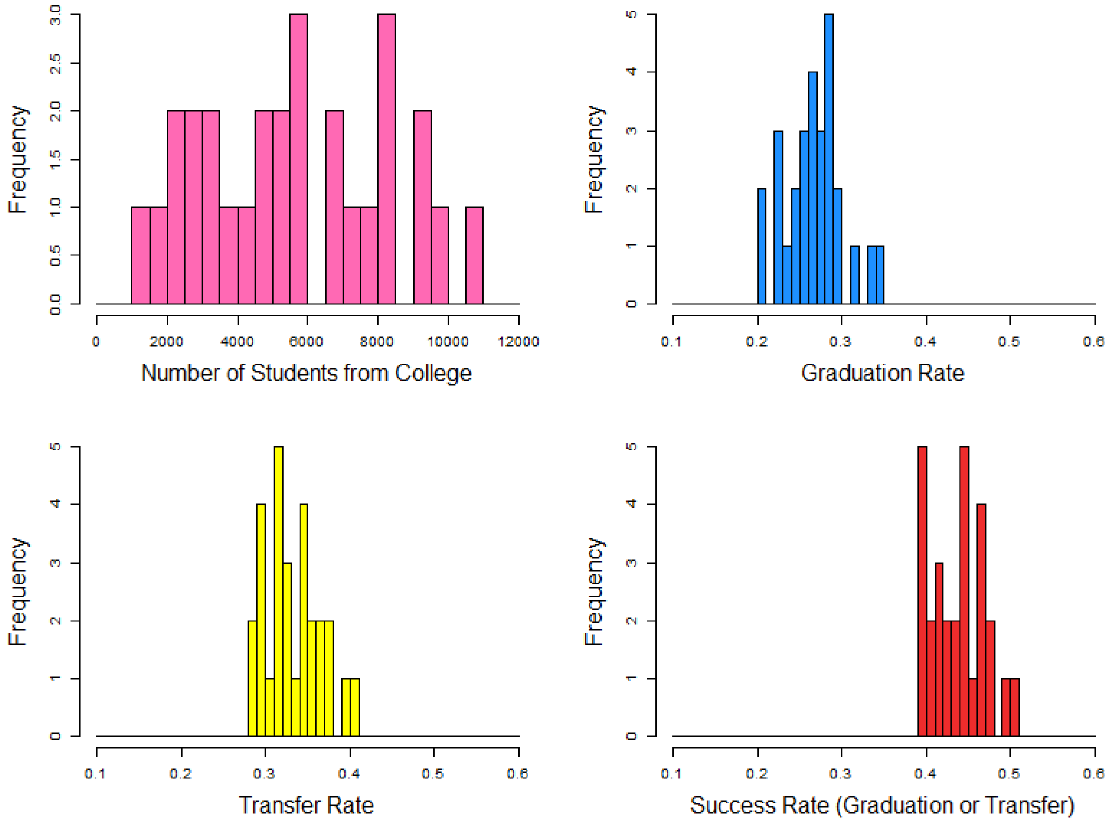

Fig. S5. Inferred probability mass function of either transferring or graduating (success point) at a given number of credits for the complete dataset of students. Most classes are 5 credits, so the probability of a success point at a multiple of 5 is higher.

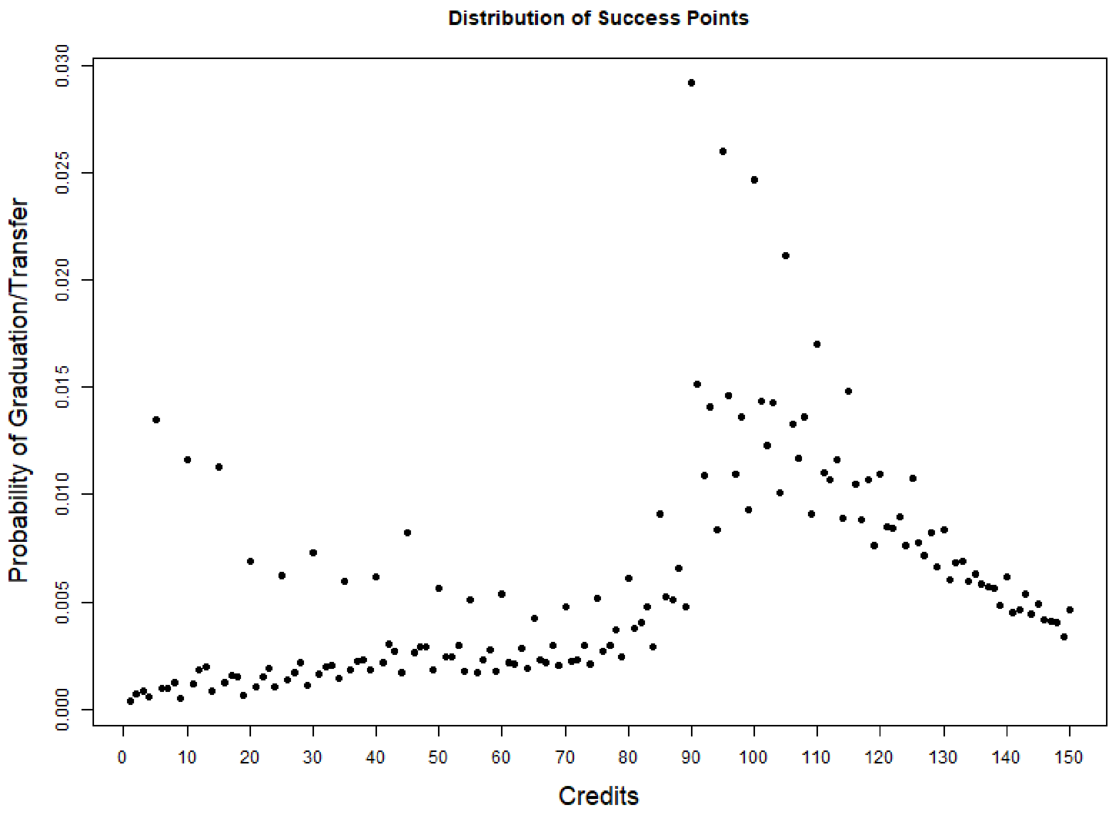

Fig. S6. Probability that a randomly chosen student will graduate/transfer at a given credit level (smoothed). Specifically, this is the smoothed inferred probability mass function (Figure S5) of either transferring or graduating (success point) at a given number of credits for the complete dataset of students.

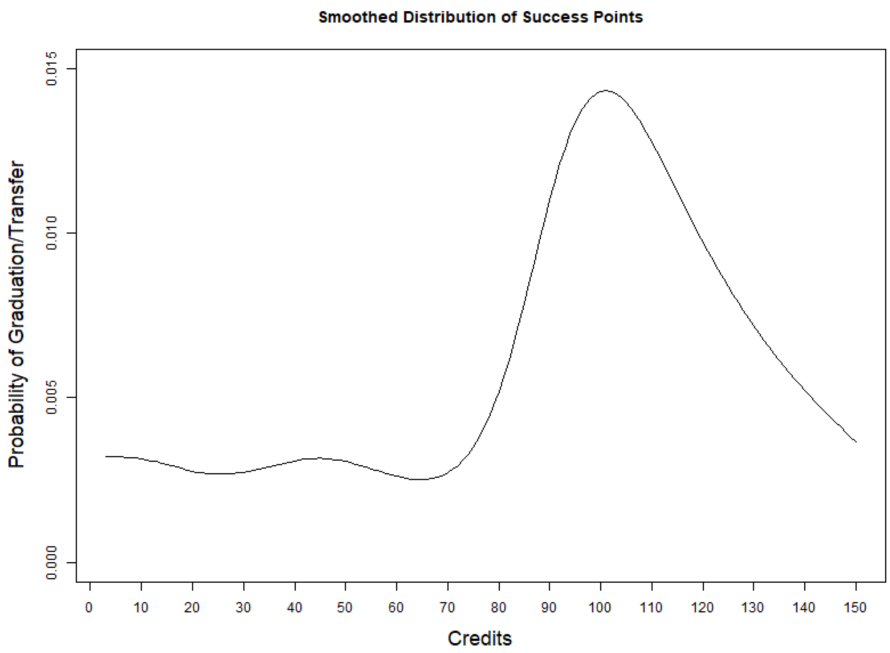

Fig. S7. Visual description of the creation of the data used in the QQ plots and in the reconstruction of dropout rates. Each value in the histogram in the bottom right was generated by taking the minimum of a randomly chosen point from the bottom left and from the top right distributions.

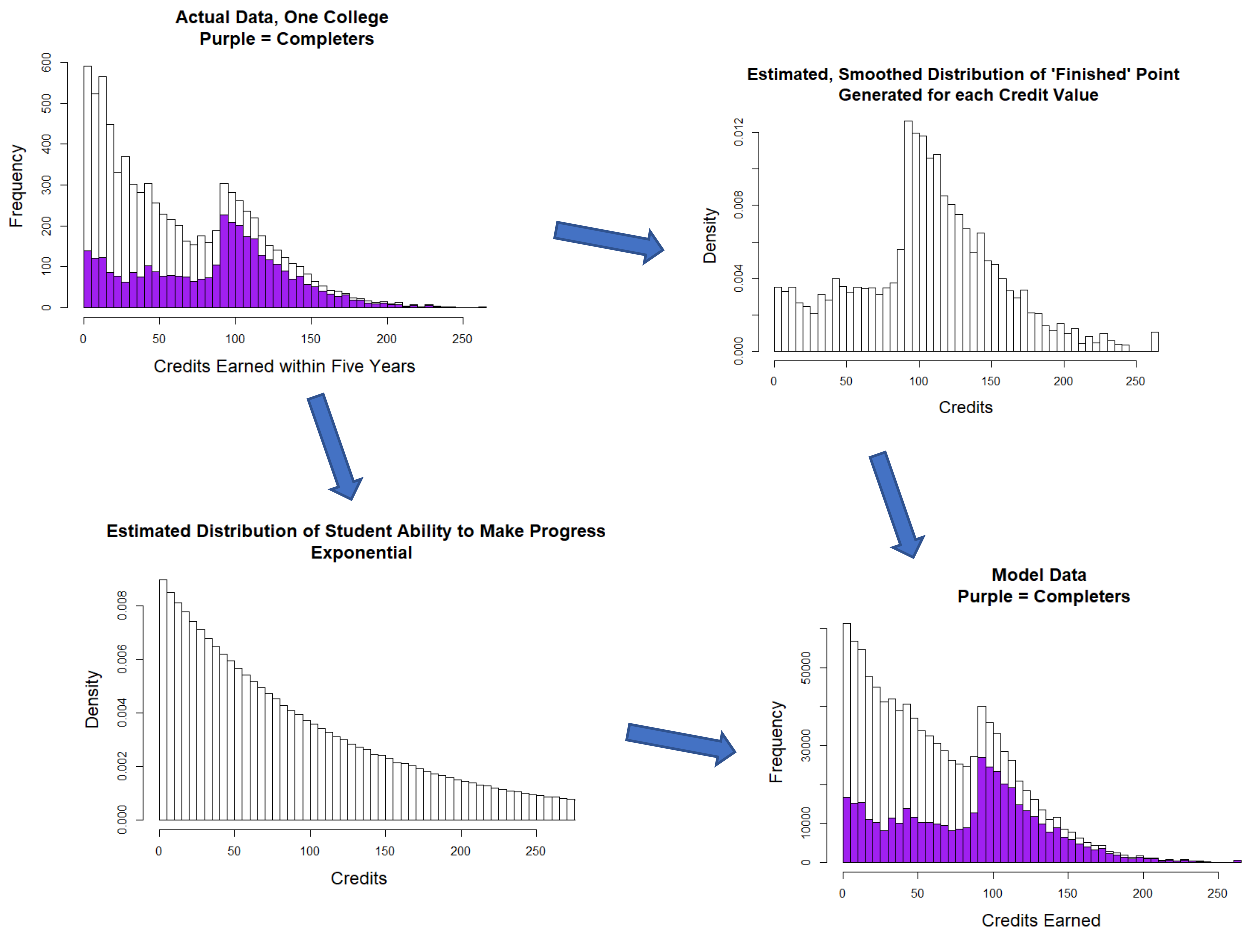

Table S1. Akaike Information Criterion (AIC) values for each college-year cohort and model. Smaller AIC's represent a better fit.

	Normal	Exponential	Power Law
College 10, Cohort 1	2750	2726	3069
College 10, Cohort 2	2957	2915	3222
College 10, Cohort 3	2796	2759	3026
College 10, Cohort 4	3188	3153	3515
College 10, Cohort 5	3567	3514	3865
College 20, Cohort 1	1868	1829	1971
College 20, Cohort 2	1686	1670	1907
College 20, Cohort 3	2221	2185	2320
College 20, Cohort 4	1707	1682	1828
College 20, Cohort 5	2282	2254	2525
College 30, Cohort 1	5403	5348	5936
College 30, Cohort 2	5902	5847	6507
College 30, Cohort 3	6392	6337	7063
College 30, Cohort 4	7941	7854	8675
College 30, Cohort 5	9550	9474	10583
College 40, Cohort 1	8361	8240	9045
College 40, Cohort 2	8566	8456	9367
College 40, Cohort 3	8665	8552	9418
College 40, Cohort 4	9486	9360	10367
College 40, Cohort 5	9780	9640	10786
College 50, Cohort 1	9875	9761	10832
College 50, Cohort 2	9979	9874	10981
College 50, Cohort 3	11470	11306	12442
College 50, Cohort 4	13335	13185	14653
College 50, Cohort 5	12024	11898	13295
College 62, Cohort 1	9613	9499	10634
College 62, Cohort 2	7526	7455	8302
College 62, Cohort 3	8098	8043	9052
College 62, Cohort 4	8238	8156	9079
College 62, Cohort 5	8572	8498	9467
College 63, Cohort 1	7753	7593	8290
College 63, Cohort 2	7826	7680	8520
College 63, Cohort 3	6214	6095	6527

College 63, Cohort 4	7700	7561	8363
College 63, Cohort 5	8127	7973	8738
College 64, Cohort 1	3668	3580	3833
College 64, Cohort 2	3751	3656	3972
College 64, Cohort 3	3614	3517	3772
College 64, Cohort 4	4291	4211	4651
College 64, Cohort 5	4141	4050	4336
College 70, Cohort 1	6735	6682	7509
College 70, Cohort 2	5488	5447	6146
College 70, Cohort 3	6359	6321	7040
College 70, Cohort 4	6328	6282	7010
College 70, Cohort 5	6345	6289	6997
College 90, Cohort 1	12277	12129	13492
College 90, Cohort 2	15080	14876	16383
College 90, Cohort 3	13030	12880	14161
College 90, Cohort 4	12539	12405	13688
College 90, Cohort 5	12679	12551	13835
College 100, Cohort 1	13209	13148	14927
College 100, Cohort 2	13423	13364	15123
College 100, Cohort 3	14043	13979	15741
College 100, Cohort 4	12666	12584	14055
College 100, Cohort 5	12977	12865	14376
College 111, Cohort 1	7633	7549	8475
College 111, Cohort 2	7547	7455	8248
College 111, Cohort 3	6631	6581	7322
College 111, Cohort 4	6828	6781	7611
College 111, Cohort 5	6523	6484	7265
College 112, Cohort 1	6122	6065	6772
College 112, Cohort 2	6245	6181	6839
College 112, Cohort 3	6574	6517	7247
College 112, Cohort 4	5463	5417	6005
College 112, Cohort 5	4691	4646	5156
College 121, Cohort 1	2946	2927	3311
College 121, Cohort 2	3075	3041	3339
College 121, Cohort 3	2874	2849	3145
College 121, Cohort 4	3410	3372	3691
College 121, Cohort 5	2520	2482	2656
College 130, Cohort 1	3004	2968	3249
College 130, Cohort 2	4136	4085	4507

College 130, Cohort 3	4341	4287	4684
College 130, Cohort 4	4341	4283	4745
College 130, Cohort 5	4747	4679	5098
College 140, Cohort 1	9103	8997	9915
College 140, Cohort 2	10174	10065	11062
College 140, Cohort 3	11803	11685	12927
College 140, Cohort 4	18412	18195	20231
College 140, Cohort 5	14895	14623	16104
College 150, Cohort 1	5325	5280	5913
College 150, Cohort 2	5070	5034	5613
College 150, Cohort 3	5799	5754	6342
College 150, Cohort 4	6266	6194	6840
College 150, Cohort 5	6267	6207	6831
College 160, Cohort 1	4092	4064	4569
College 160, Cohort 2	4113	4087	4612
College 160, Cohort 3	4804	4776	5394
College 160, Cohort 4	4304	4262	4705
College 160, Cohort 5	4737	4697	5213
College 171, Cohort 1	4613	4579	5180
College 171, Cohort 2	4547	4501	5100
College 171, Cohort 3	4000	3964	4363
College 171, Cohort 4	3669	3646	4104
College 171, Cohort 5	2655	2642	2970
College 172, Cohort 1	12262	12056	13145
College 172, Cohort 2	11257	11141	12515
College 172, Cohort 3	13179	12930	13806
College 172, Cohort 4	12038	11881	13005
College 172, Cohort 5	12086	11892	12890
College 180, Cohort 1	2104	2094	2391
College 180, Cohort 2	2348	2316	2498
College 180, Cohort 3	2408	2380	2621
College 180, Cohort 4	2252	2242	2518
College 180, Cohort 5	1518	1506	1645
College 190, Cohort 1	9970	9839	10815
College 190, Cohort 2	11231	11081	12077
College 190, Cohort 3	11343	11192	12285
College 190, Cohort 4	10197	10077	11007
College 190, Cohort 5	10460	10343	11404
College 200, Cohort 1	2306	2276	2461

College 200, Cohort 2	3202	3165	3453
College 200, Cohort 3	3537	3497	3858
College 200, Cohort 4	3682	3627	3986
College 200, Cohort 5	4062	4026	4468
College 210, Cohort 1	7032	6963	7776
College 210, Cohort 2	7914	7847	8768
College 210, Cohort 3	8112	8037	8928
College 210, Cohort 4	10750	10629	11761
College 210, Cohort 5	10390	10259	11262
College 220, Cohort 1	8835	8770	9883
College 220, Cohort 2	9142	9060	10145
College 220, Cohort 3	10811	10713	12001
College 220, Cohort 4	11044	10948	12292
College 220, Cohort 5	12373	12245	13659
College 230, Cohort 1	10038	9941	11158
College 230, Cohort 2	10276	10200	11450
College 230, Cohort 3	10172	10107	11371
College 230, Cohort 4	11159	11076	12511
College 230, Cohort 5	11360	11308	12849
College 240, Cohort 1	7249	7160	7916
College 240, Cohort 2	6390	6292	6921
College 240, Cohort 3	6401	6330	7046
College 240, Cohort 4	6974	6866	7552
College 240, Cohort 5	7048	6959	7696
College 300, Cohort 1	5721	5644	6233
College 300, Cohort 2	5952	5876	6491
College 300, Cohort 3	6725	6648	7328
College 300, Cohort 4	7260	7198	7984
College 300, Cohort 5	7463	7378	8185

Table S2. Summary statistics by student.

Number of students	156,712
Transferred	32.8%
Graduated	26.2%
Transferred or Graduated	43.2%
Credits earned (mean)	60.4
Credits earned (sd)	45.9
Percent underrepresented minority (not white/Asian)	28.7%
Male	47.4%
Age (mean)	21.4
Age (sd)	7.2

REFERENCES AND NOTES

1. U.S. Department of Education, *Digest of Education Statistics* (U.S. Department of Education, 2017); https://nces.ed.gov/programs/digest/d17/tables/dt17_219.57.asp.

2. D. Shapiro, A. Dundar, F. Huie, P. K. Wakhungu, A. Bhimdiwala, S. E. Wilson, Completing college: A national view of student completion rates—Fall 2012 Cohort (Signature Report No. 16, National Student Clearinghouse Research Center, 2018); https://nscresearchcenter.org/wp-content/uploads/SignatureReport16.pdf.

3. J. Scott-Clayton, P. M. Crosta, C. R. Belfield, Improving the targeting of treatment: Evidence from college remediation. *Educ. Eval. Policy Anal.* **36**, 371–393 (2014).

4. M. C. Long, D. Conger, P. Iatarola, Effects of high school course-taking on secondary and postsecondary success. *Am. Educ. Res. J.* **49**, 285–322 (2012).

5. S. F. Porchea, J. Allen, S. Robbins, R. P. Phelps, Predictors of long-term enrollment and degree outcomes for community college students: Integrating academic, psychosocial, socio-demographic, and situational. *J. Higher Educ.* **81**, 680–708 (2010).

6. E. T. Pascarella, P. T. Terenzini, *How College Affects Students, Volume 2*: *A Third Decade of Research* (Jossey-Bass, 2005).

7. J. Johnson, J. Rochkind, *With Their Whole Lives Ahead of Them*: *Myths and Realities About Why So Many Students Fail to Finish College* (2009); http://www.publicagenda.org/files/theirwholelivesaheadofthem.pdf.

8. S. Goldrick-Rab, Addressing community college completion rates by securing students' basic needs. *New Dir. Community Coll.* **2018**, 7–16 (2018).

9. J. Boaler, Open and closed mathematics: Student experiences and understandings. *J. Res. Math. Educ.* **29**, 41–62 (1998).

10. R. D. Cox, You've got to learn the rules: A classroom-level look at low pass rates in developmental math. *Community Coll. Rev.* **43**, 264–286 (2015).

11. L. Cuban, *Inside the Black Box of Classroom Practice*: *Change Without Reform in American Education* (Harvard Education Press, 2013).

12. W. N. Grubb, *From Black Box to Pandora's Box*: *Evaluating Remedial/Developmental Education* (2001).

13. M. J. Mayhew, A. N. Rockenbach, N. A. Bowman, T. A. Seifert, G. C. Wolniak, E. T. Pascarella, P. T. Terenzini, *How College Affects Students, Volume 3*: *21st Century Evidence that Higher Education Works* (Jossey-Bass, 2016).

14. R. Putnam, Social capital: Measurement and consequences. *Can. J. Policy Res.* **2**, 41–51 (2001).

15. J. S. Coleman, Social capital in the creation of human capital. *Am. J. Sociol.* **94**, S95–S120 (1988).

16. M. Granovetter, The strength of weak ties: A network theory revisited. *Sociol. Theory* **1**, 201–233 (1983).

17. G. S. Becker, *Human Capital*: *A Theoretical and Empirical Analysis with Special Reference to Education* (The University of Chicago Press, ed. 3, 1994).

18. G. Castex, E. K. Dechter, The changing roles of education and ability in wage determination. *J. Labor Econ.* **32**, 685–710 (2014).

19. D. J. Deming, The growing importance of social skills in the labor market. *Q. J. Econ.* **132**, 1593–1640 (2017).

20. A. Killewald, F. T. Pfeffer, J. N. Schachner, Wealth inequality and accumulation. *Annu. Rev. Sociol.* **43**, 379–404 (2017).

21. M. B. Mulder, S. Bowles, T. Hertz, A. Bell, J. Beise, G. Clark, I. Fazzio, M. Gurven, K. Hill, P. L. Hooper, W. Irons, H. Kaplan, D. Leonetti, B. Low, F. Marlowe, R. McElreath, S. Naidu, D. Nolin, P. Piraino, R. Quinlan, E. Schniter, R. Sear, M. Shenk, E. A. Smith, C. von Rueden, P. Wiessner, Intergenerational wealth transmission and the dynamics of inequality in small-scale societies. *Science* **326**, 682–688 (2009).

22. B. Milanovic, P. H. Lindert, J. G. Williamson, Pre-industrial inequality. *Econ. J.* **121**, 255–272 (2011).

23. Y. Tao, X. Wu, T. Zhou, W. Yan, Y. Huang, H. Yu, B. Mondal, V. M. Yakovenko, Exponential structure of income inequality: Evidence from 67 countries. *J. Econ. Interact. Coord.* **14**, 345–376 (2019).

24. M. Nirei, W. Souma, A two factor model of income distribution dynamics. *Rev. Income Wealth* **53**, 440–459 (2007).

25. S. Goldrick-Rab, J. Richardson, A. Hernandez, *Hungry and Homeless in College*: *Results From a National Study of Basic Needs Insecurity in Higher Education* (2017); http://wihopelab.com/publications/Hungry-and-Homeless-in-College-Report.pdf.

26. R. J. Herrnstein, C. Murray, *The Bell Curve*: *Intelligence and Class Structure in American Life* (The Free Press, 1994).

27. S. R. Sirin, Socioeconomic status and academic achievement: A meta-analytic review of research. *Rev. Educ. Res.* **75**, 417–453 (2005).

28. E. W. Dillon, J. A. Smith, Determinants of the match between student ability and college quality. *J. Labor Econ.* **35**, 45–66 (2017).

29. A. Lareau, *Unequal Childhoods* (University of California Press, ed. 2, 2011).

30. A. G. Wilson, The use of the concept of entropy in system modelling. *J. Oper. Res. Soc.* **21**, 247–265 (1970).

31. J. Harte, *Maximum Entropy and Ecology*: *A Theory of Abundance, Distribution, and Energetics* (Oxford Univ. Press, 2011).

32. E. W. Montroll, On the entropy function in sociotechnical systems. *Proc. Natl. Acad. Sci. U.S.A.* **78**, 7839–7843 (1981).

33. A. De Martino, D. De Martino, An introduction to the maximum entropy approach and its application to inference problems in biology. *Heliyon* **4**, e00596 (2018).

34. A. Drăgulescu, V. M. Yakovenko, Statistical mechanics of money. *Eur. Phys. J. B.* **17**, 723–729 (2000).

35. L. Muchnik, S. Aral, S. J. Taylor, Social influence bias: A randomized experiment. *Science* **341**, 647–651 (2013).

36. V. M. Yakovenko, J. B. Rosser Jr, Colloquium: Statistical mechanics of money, wealth, and income. *Rev. Mod. Phys.* **81**, 1703–1725 (2009).

37. D. Easley, J. Kleinberg, *Networks, Crowds, and Markets*: *Reasoning About a Highly Connected World* (Cambridge Univ. Press, 2010); https://www.cs.cornell.edu/home/kleinber/networks-book/.

38. S. Reardon, in *Whither Opportunity? Rising Inequality and the Uncertain Life Chances of Low-Income Children*, R. Murnane, G. Duncan, Eds. (Russell Sage Foundation Press, 2011).

39. A. S. Morris, J. S. Silk, L. Steinberg, S. S. Myers, L. R. Robinson, The role of the family context in the development of emotion regulation. *Soc. Dev.* **16**, 361–388 (2007).

40. F. T. Pfeffer, A. Killewald, Generations of advantage. Multigenerational correlations in family wealth. *Soc. Forces* **96**, 1411–1442 (2018).

41. D. Jenkins, T. Ellwein, J. Wachen, M. R. Kerrigan, S.-W. Cho, *Achieving the Dream Colleges in Pennsylvania and Washington State*: *Early Progress toward Building a Culture of Evidence* (2009); https://academiccommons.columbia.edu/doi/10.7916/D8445JKS.

42. W. S. Moore, E. Lardner, G. Malnarich, M. Davis, *Rethinking Pre-College Math*: *Pedagogy, Professional Responsibility, and Student Success* (2013); http://citeseerx.ist.psu.edu/viewdoc/download?doi=10.1.1.666.5369&rep=rep1&type=pdf).

43. C. L. Quarles, M. Davis, Is learning in developmental math associated with community college outcomes? *Community Coll. Rev.* **45**, 33–51 (2017).

44. D. Starch, E. C. Elliott, Reliability of grading work in mathematics. *School Rev.* **21**, 254–259 (1913).

45. D. Starch, E. C. Elliott, Reliability of the grading of high-school work in English. *School Rev.* **20**, 442–457 (1912).

46. H. M. Brimi, Reliability of grading high school work in English. *Pract. Assess. Res. Eval.* **16**, 1–12 (2011).

47. S. A. Myers, A. Sharma, P. Gupta, J. Lin, Information network or social network? The structure of the twitter follow graph. *Proc. 23rd Int. Conf. World Wide Web*, 493–498 (2014).

48. D. De Solla Price, A general theory of bibliometric and other cumulative advantage processes. *J. Am. Soc. Inf. Sci.* **27**, 292–306 (1976).

49. A. Drăgulescu, V. M. Yakovenko, Exponential and power-law probability distributions of wealth and income in the United Kingdom and the United States. *Phys. A Stat. Mech. Appl.* **299**, 213–221 (2001).

50. Z. Kolenovic, D. Linderman, M. M. Karp, Improving student outcomes via comprehensive supports : Three-year outcomes from CUNY's accelerated study in associate programs (ASAP). *Community Coll. Rev.* **41**, 271–291 (2013).

51. H. M. Levin, E. Garcia, Benefit-cost analysis of accelerated study in associate programs (ASAP) of the City University of New York (CUNY) (2013); https://www1.nyc.gov/assets/opportunity/pdf/Levin_ASAP_Benefit_Cost_Report_FINAL_05212013.pdf.

52. C. T. Clotfelter, H. F. Ladd, C. G. Muschkin, J. L. Vigdor, Success in community college: Do institutions differ? *Res. High. Educ.* **54**, 805–824 (2013).

53. R Core Team, *R*: *A Language and Environment for Statistical Computing* (2018); http://www.r-project.org/.

54. T. W. Yee, *VGAM*: *Vector Generalized Linear and Additive Models* (2018); https://cran.r-project.org/package=VGAM.

55. P. M. Crosta, Intensity and attachment: How the chaotic enrollment patterns of community college students relate to educational outcomes. *Community Coll. Rev.* **42**, 118–142 (2014).

56. D. Chotikapanich, *Modeling Income Distributions and Lorenz Curves* (Springer, 2008).

57. F. Dyson, A meeting with Enrico Fermi. *Nature* **427**, 297 (2004).

58. D. Slottje, Modeling income distributions and Lorenz curves. *J. Econ. Inequal.* **8**, 525–526 (2010).

59. J. M. Henle, N. J. Horton, S. J. Jakus, Modelling inequality with a single parameter, in *Modeling Income Distributions and Lorenz Curves*, D. Chotikapanich, Ed. (Springer, 2008), pp. 255–269.

60. M. Levy, Are rich people smarter ? *J. Econ. Theory* **110**, 42–64 (2003).

61. O. S. Klass, O. Biham, M. Levy, O. Malcai, S. Solomon, The Forbes 400 and the Pareto wealth distribution. *Econ. Lett.* **90**, 290–295 (2006).

62. J. B. Davies, S. Sandström, A. Shorrocks, E. N. Wolff, *The World Distribution of Household Wealth* (2008).

63. A. Sala, H. Zheng, B. Zhao, S. Gaito, Brief announcement: Revisiting the power-law degree distribution for social graph analysis. *Proc. 29th ACM SIGACT-SIGOPS Symp. Princ. Distrib. Comput.*, 400–401 (2010).

64. A.-L. Barabási, R. Albert, Emergence of scaling in random networks. *Science* **286**, 509–512 (1999).

65. H. Ebel, L.-I. Mielsch, S. Bornholdt, Scale-free topology of e-mail networks. *Phys. Rev. E* **66**, 035103 (2002).

66. R. I. M. Dunbar, Neocortex size as a constraint on group size in primates. *J. Hum. Evol.* **22**, 469–493 (1992).

67. B. Gonçalves, N. Perra, A. Vespignani, Modeling users' activity on twitter networks: Validation of Dunbar's number. *PLOS ONE* **6**, e22656 (2011).

68. H.-Y. Chan, X. Wang, Momentum through course-completion patterns among 2-year college students beginning in STEM: Variations and contributing factors. *Res. High. Educ.* **59**, 704–743 (2018).
